# Pharmacochemical Study of Multitarget Amino Acids’ Hybrids: Design, Synthesis, *In vitro,* and *In silico* Studies

**DOI:** 10.2174/0115734064279653240125081042

**Published:** 2024-02-09

**Authors:** Ioannis Fotopoulos, Eleni Pontiki, Dimitra Hadjipavlou-Litina

**Affiliations:** 1 Department of Pharmaceutical Chemistry, School of Pharmacy, Faculty of Health Sciences, Aristotle University of Thessaloniki, Thessaloniki, 54124, Greece

**Keywords:** Multitarget, amino acids, hybrids, inflammation, cyclooxygenase inhibitors, lipoxygenase inhibitors, Alzheimer’s disease, neuro-inflammation

## Abstract

**Introduction:**

Neuro-inflammation is a complex phenomenon resulting in several disorders. ALOX-5, COX-2, pro-inflammatory enzymes, and amino acid neurotransmitters are tightly correlated to neuro-inflammatory pathologies. Developing drugs that interfere with these targets will offer treatment for various diseases.

**Objective:**

Herein, we extend our previous research by synthesizing a series of multitarget hybrids of cinnamic acids with amino acids recognized as neurotransmitters.

**Methods:**

The synthesis was based on an *in silico* study of a library of cinnamic amide hybrids with glycine, γ- aminobutyric, and L - glutamic acids. Drug-likeness and ADMET properties were subjected to *in silico* analysis. Cinnamic acids were derived from the corresponding aldehydes by Knoevenagel condensation. The synthesis of the amides followed a two-step reaction with 1-hydroxybenzotriazole monohydrate and 1-ethyl-3-(3-dimethylaminopropyl) carbodiimide hydrochloride in dry dichloromethane and the corresponding amino acid ester hydrochloride salt in the presence of N,N,-diisopropyl-Nethylamine.

**Results:**

The structure of the synthesized compounds was confirmed spectrophotometrically. The new compounds, such as lipoxygenase, cyclooxygenase-2, lipid peroxidation inhibitors, and anti-inflammatories, were tested *in vitro*. The compounds exhibited LOX inhibition with IC_50_ values in the low µM region).

**Conclusion:**

Compounds **18a**, **23b**, and **11c** are strong lipid peroxidation inhibitors (99%, 78%, and 92%). Compound **28c** inhibits SLOX-1 with IC_50_ =8.5 µM whereas **11a** and **22a** highly inhibit COX-2 (IC_50_ 6 and 5 µM Hybrids **14c** and **17c** inhibit both enzymes. Compound **29c** showed the highest anti-inflammatory activity (75%). The *in silico* ADMET properties of **14c** and **11a** support their drug-likeness.

## 1 INTRODUCTION

**Scheme 1:**
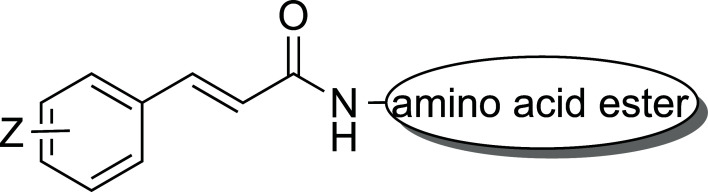
General structure of the synthesized hybrids.

Several neurodegenerative disorders are related to neuro-inflammation, defined as an inflammatory response within the brain or spinal cord. Producing cytokines, chemokines, reactive oxygen species, and secondary messengers mediates this inflammation. The brain is a metabolically active organ since it largely depends on the metabolism of PUFAs (polyunsaturated fatty acids). Recent reports concluded that ALOX-5, COX-2, pro-inflammatory enzymes, and amino acid neurotransmitters are tightly correlated to neuro-inflammatory pathologies such as Alzheimer’s and Parkinson’s. Several reports indicate the role of the ALOX-5 and COX-2 enzymes in developing these diseases [1-3]. ALOX-5 is considered a pro-inflammatory enzyme [4] metabolizing arachidonic acid to a plethora of products, *i.e*., leukotrienes, lipoxins, *etc*., exerting its central role in inflammation through these final products. Although ALOX-5 is constantly expressed in the central nervous system [5], its translation is upregulated in Alzheimer’s disease, as reports are linking its activity with higher Αβ amyloid production [6] and loss of dopaminergic neuron activity [7]. COX-2 is an inducible isoenzyme that converts arachidonic acid to several pro-inflammatory compounds, prostanoids, prostaglandins, and thromboxanes. Even though COX-2 is constantly expressed in the brain [8], its overexpression resulted in higher Αβ amyloid production [9], which is mainly due to higher PGE2 production [10]. The overproduction of PGE2 is also considered the main reason for the onset of Parkinson’s disease, mainly due to the induction of the release of the excitatory neurotransmitter glutamic acid [11]. The mutual regulation between excitatory and inhibitory mechanisms governs the brain’s functionality. Some of the most important neurotransmitters are the amino acids glycine, γ- aminobutyric acid (GABA), and L - glutamic acid. Although Glycine and GABA [12] are the main sedative neurotransmitters, glycine exerts both inhibitory and sedative activities [13]. It is known that glutamic acid mediates mostly its excitatory neurotransmission [14], acting on its receptors - collectively known as iGluRs - comprising AMPA (a-amino-3-hydroxy-5-methyl-4- isoxazolepropionic acid), kainate, NMDA (N-methyl-D-aspartic acid) and δ receptors. NMDARs rely on the concurrent binding of glutamic acid and glycine/D-serine for their activation. Thus, glycine exerts its excitatory activity mainly by this route. However, several reports indicate that δ receptors are considered “glutamate-free” regarding their activation [15-17]. The uncontrolled disruption of the sedative or activation of the excitatory neurotransmission pathways leads to the massive calcium influx, activating the pro-inflammatory enzymes ALOX-5 and COX-2. Reactive oxygen species (ROS), although vital for cell function and regulation, are considered pro-inflammatory messengers mainly due to their adverse effects on cell vitality. Several neuroinflammatory diseases are directly correlated with the unregulated production of ROS. Cinnamic acids present a plethora of biological activities, *e.g*., anti-inflammatory [18, 19] and neuroprotective [20]. Phenolic cinnamic acids, such as ferulic and caffeic acids, and their derivatives were studied as neuroprotective agents, showing promising results [20]. Herein, in continuation to our previous work on multitarget cinnamic hybrids [21, 22], we will describe the modeling and the *in silico* drug-likeness studies, the theoretically calculated ADMET properties of amino acids’ hybrids of cinnamic acids (Scheme **1**), the synthesis and their *in vitro* pharmacochemical evaluation as LOX, COX-2, lipid peroxidation inhibitors and as anti-inflammatories using the albumin denaturation assay. The new hybrids' two chemical entities/ pharmacophores are several substituted cinnamic acids and amino acids (glycine, γ- aminobutyric acid (GABA), and L - glutamic acid). These hybrids are expected to present better pharmacochemical profiles and pharmacokinetic properties since molecular hybridization is often implemented to yield compounds more effective than their parent molecule [23].

## 2 MATERIALS AND METHODS

### 2.1 General Information

All chemicals were used as supplied. Starting materials were purchased from commercial sources (Merck KGaA, Darmstadt, Germany; Fluorochem Ltd, Derbyshire, United Kingdom; Alfa Aesar, Kandel, Germany) or synthesized following the methodologies described. All biochemical reagents were purchased from commercial sources. Soybean lipoxygenase, sodium linoleate, and NDGA were obtained from Sigma Chemical, Co. (St Louis, MO, USA), whereas COX-2 ovine was by Cayman (USA). The solvents used were of reagent grade. Column chromatography was performed using Merck 230 to 400 mesh silica gel. Thin-layer chromatography was performed on Merck 0.2 mm aluminum-based silica gel 60 F254 plates and visualized using ultraviolet light. Melting points were determined using a Mel-Temp II (Laboratory Devices, USA) apparatus and were uncorrected. Infrared (IR) spectra were recorded on a Perkin-Elmer spectrum BX FT-IR spectrometer as potassium bromide disks. NMR spectra were recorded on a Bruker Avance 400 spectrometer (400 MHz for ^1^H and 100 MHz for ^13^C) or an Agilent 500/54 (DD2) spectrometer (500 MHz for ^1^H and 125 MHz for ^13^C) or a Bruker Avance III spectrometer (250 MHz for ^1^H and 63 MHz for ^13^C) and are stated accordingly. The chemical shifts are reported in δ (ppm) and are relative to the central peak of the solvent (which is stated in each case). The following abbreviations are used: s=singlet, d=doublet, dd=doublet of doublets, t=triplet, dt = doublet of triplets, q=quartet, m=multiplet, br=broad. LC-MS (ESI - MS) spectra were recorded on a Shimadzu LCMS-2010 EV instrument. HRMS (ESI-MS) spectra were recorded on an Agilent QTOF Mass Spectrometer G6540B with dual AJS ESI-MS. The optical activity experiments were carried out on an A. Kruss Optronic in a 50 mm cuvette.

### 2.2 Docking Studies

A compound in-house library of 200 cinnamic amino acids hybrids was compiled based on our laboratory background and submitted to docking-based virtual screening on soybean lipoxygenase-1 (PDB ID: 3PZW) and cyclooxygenase-2 (PDB code: 1CX2) resulting in the identification of potential inhibitors. The above enzymes were selected to be by the enzymes used in the *in vitro* assays.

#### 2.2.1 Docking Studies on SLOX-1

For the docking studies, the soybean lipoxygenase-1 (PDB ID: 3PZW) was selected and visualized using USCF Chimera [24]. Water molecules were removed, and the missing residues were added using Modeller [25]. The hydrogen atoms and the AMBER99SBILDN charges were added. The iron atom charge was set to +2.0 with no restraint applied. Ligand’s three-dimensional coordinates were generated and minimized with OpenBabel [26] applying the MMFF94 force field [27] to generate ligand topologies, and parameters ACPYPE (AnteChamber PYthon Parser interfacE) [28] was used, operating AnteChamber [29]. GROMACS 4.6 [30] was used as the molecular dynamics simulation toolkit to conduct the energy minimization process using the AMBER99SB-ILDN force field [31]. Docking was performed using AutoDock Vina 1.1.2 [32] by applying a 100, 70, 70 Å (in the x,y, and z axes, respectively) grid box. The docking input files were generated, and the results were analyzed using UCSF Chimera. Docking calculations were carried out with an exhaustiveness value of 10 and a maximum output of 20 docking modes.

#### 2.2.2 Docking Studies on COX-2

The human cyclooxygenase-2 (PDB code: 1CX2) was used for the docking studies due to the high homology with the ovine COX-2 used in the *in vitro* experiments. Alignment of the primary sequences of the two proteins was accomplished using UniProt (www.uniprot.org), and the results revealed 86.4% homology. The same procedure described above was used to conduct molecular docking studies on COX-2. The grid box was 25 Å in the x, y, and z axes, centered in the catalytic site. The docking calculations were carried out with an exhaustiveness value of 10 and a maximum output of 20 poses.

### 2.3 Drug-likeness and ADMET Properties

Considering their drug-likeness, the designed compounds were subjected to *in silico* studies of their ADMET properties. The online platforms Molinspiration (www.molinspiration.com) [33] (accessed on 14/06/2021), SwissADME (http://www.swissadme.ch/) (accessed on 27/07/2021), PreADMET (https://preadmet.bmdrc.kr/adme/) (accessed on 15/05/2021), Molsoft (https://molsoft.com/mprop/) (accessed on 17/08/2021), LiverTox Workspace (https://livertox.univie.ac.at/) (accessed on 14/09/2021) GLORYx (https://nerdd.univie.ac.at/gloryx/) (accessed on 06/05/2021) and CypRules (https://cyprules.cmdm.tw/) (accessed on 06/05/2021) were used.

## 3 EXPERIMENTAL

### 3.1 Chemistry

#### 3.1.1 General Procedure for the Synthesis of 4-(4-bromobenzyloxy)-benzaldehyde (1).

**Scheme 2:**
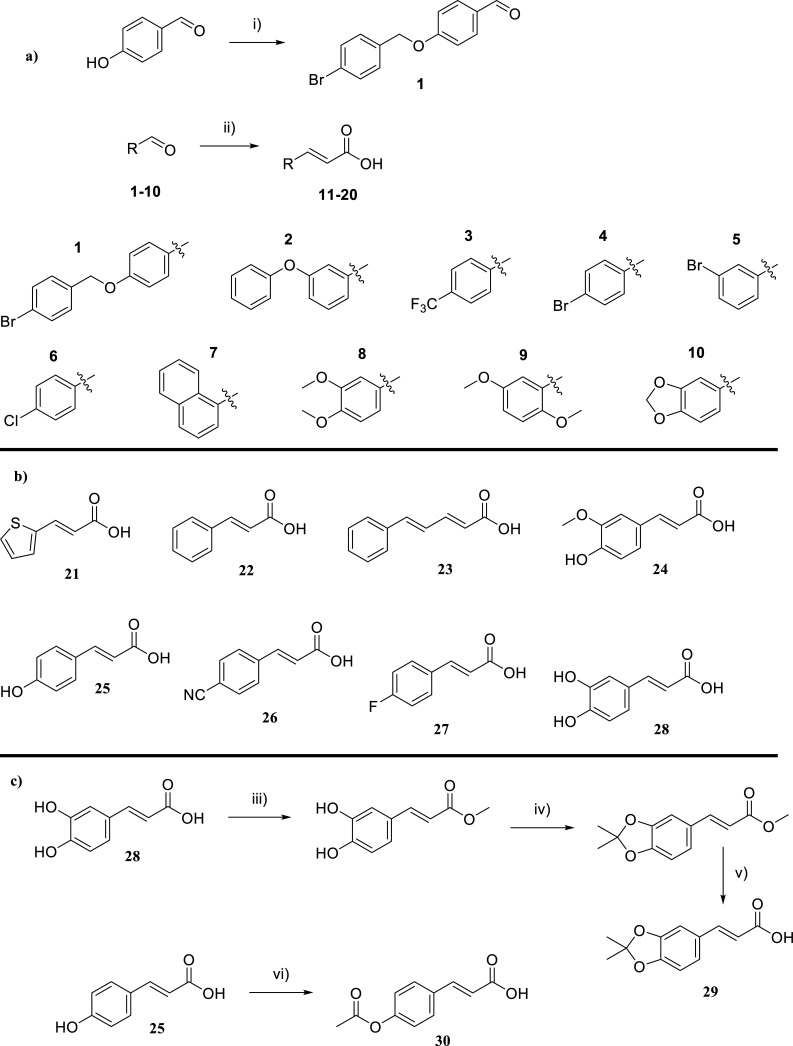
**a**) Reagents and conditions: i) 4-hydroxybenzaldehyde (4.1 mmol, 1.0 equiv.), 4-bromobenzylbromide (4.1 mmol, 1.0 equiv.), K_2_CO_3_ (8.2 mmol, 2.0 equiv.)), acetone (10 mL), reflux, 1h ii) appropriate aldehyde (5.0 mmol, 1.0 equiv.), malonic acid (7.5 mmol., 1.25 equiv.), pyridine (10 mL), piperidine (1.25 mmol, 0.25 eq), reflux; **b**) Structures of the commercially available (compounds **21**, **22**, **23**, **24**, **25**, **28**) or previously synthesized cinnamic acids (compounds **26**, **27**) that were used without further purification [33]. **c**) iii) trans-caffeic acid (5.55 mmol), MeOH (20 mL), H_2_SO_4_ (2-3 drops), reflux, 24 h. iv) methyl (E)-3-(3,4-dihydroxyphenyl)acrylate (3.09 mmol, 1.0 equiv.), DMP (12.98 mmol, 4.2 equiv.), pTSA (1.7 mmol, 0.06 equiv.), acetone (10 mL), toluene (36 mL), Dean-Stark apparatus, 2 h v) methyl (E)-3-(2,2-dimethylbenzo[d][1,3]dioxol-5-yl)acrylate (2.66 mmol, 1.0 equiv.), LiOH monohydrate (6.29 mmol, 2.37 equiv.), MeOH:H_2_O (1:1 *v/v*, 20 mL), r.t.,48h. vi) p-coumaric acid (6.09 mmol, 1.0 equiv.), acetic anhydride (7.61 mmol, 1.25 equiv.), DMAP (0.609 mmol, 0.1 equiv.), pyridine (10 mL), 0°C, 2 h.

A modified procedure was followed [34] (Scheme **2a**). In a round-bottomed flask containing acetone (10 mL), 4-hydroxybenzaldehyde (500 mg, 4.1 mmol, 1.0 eq.), 4-bromobenzylbromide (1.02 g, 4.1 mmol, 1.0 eq.) and K_2_CO_3_ (1.13 g, 8.2 mmol, 2.0 eq.) were consequently added and the mixture was refluxed for 1 h. TLC monitored the reaction. The solvent was evaporated. The residue was diluted with ethyl acetate and washed with water (2 x 15 mL) and brine (1 x 15 mL). The organic layer was dried (Na_2_SO_4_), and the solvent was removed to dryness to yield quantitatively a white solid. The final product was subsequently used for the next step (Scheme **2**).

#### 3.1.2 General Procedure for the Synthesis of Cinnamic Acids 11-20

In a round-bottomed flask were consecutively added pyridine (10 mL), the appropriate aldehyde (1 equiv.), malonic acid (1.5 equiv.), piperidine (0.25 equiv.), and the mixture was refluxed. The reaction was monitored (TLC). Upon completion, the mixture was cooled to 0°C and then acidified with 2 M HCl (aq). The precipitated solid was filtered off, washed with water, dried, and recrystallized from the appropriate solvent. In case of lack of precipitation, the reaction mixture was poured into a separating funnel and extracted with ethyl acetate (3 x 15 mL). The organic layers were collected, washed with brine (2 x 15 mL), dried over Na_2_SO_4_, filtered, and evaporated to yield the desired product (see Scheme **2** procedure a).

#### 3.1.3 General Procedure for Synthesizing Cinnamic acid 29 (see Scheme 2 Procedure c)

##### 3.1.3.1 Step 1

In a round-bottomed flask containing methanol (20 mL), trans-caffeic acid is added (1.0 g, 5.56 mmol, 1.0 equiv.) along with 4 drops of concentrated H_2_SO_4_ [35]. The mixture was refluxed overnight. Upon completion of the reaction (TLC monitoring), the solvent evaporated. Water (15 mL) and saturated NaHCO_3_ solution (15 mL) were added, and the mixture was extracted with ethyl acetate. The organic layer was collected, dried over Na_2_SO_4_ and evaporated to yield methyl caffeic as a white solid.

##### 3.1.3.2 Step 2

Methyl caffeate (600 mg, 3.09 mmol, 1.0 equiv.) is added to a round-bottomed flask containing 46 mL of toluene: acetone mixture (36 mL:10 mL). Then, 2,2-dimethoxypropane (DMP) (1.35 g, 1.6 mL, 12.98 mmol 4.2 equiv.) and p-toluenesulfonic acid (PTSA) (29 mg, 1.7 mmol, 0.06 equiv.) were added and the mixture is refluxed in a Dean-Stark apparatus [36]. The solvents were evaporated after 2 hours, and the slur was subjected to column chromatography using petroleum spirit and ethyl acetate (6:1) as eluent to yield the corresponding acetonide as an oil.

##### 3.1.3.3 Step 3

In a solution of the acetonide of step 2 (622 mg, 2.66 mmol, 1.0 equiv.) in a methanol/ water mixture (20 mL, 1:1 ratio), lithium hydroxide monohydrate is added (264 mg, 6.29 mmol, 2.37 equiv.) The mixture was stirred in r.t. for 48 h and monitored chromatographically [37]. The solvent was evaporated. The pH is adjusted to 5-6 using a 10% w/v citric acid solution. The aqueous solution was extracted with ethyl acetate. The organic layer was collected, dried over Na_2_SO_4,_ and evaporated to yield the corresponding acid **29** as a yellow solid.

#### 3.1.4 General Procedure for Synthesizing Cinnamic Acid 30 (see Scheme 2 Procedure c)

In a round-bottomed flask containing pyridine (10 ml) were consequently added in 0°C, 4-hydroxycinnamic acid (1.0 gr, 6.09 mmol, 1. 0 equiv.), acetic anhydride (777 mg, 0.72 ml, 7.61 mmol, and 1. 25 equiv.) and DMAP (74 mg, 0.609 mmol, and 0.1 equiv.) [38]. The mixture was stirred at 0°C for 2 h. The reaction was monitored through TLC, and the mixture was poured onto ice. The aqueous layer was acidified with HCl 1M solution until pH=1 and was extracted with ethyl acetate (2 x 15 mL). The organic layer was collected, dried over Na_2_SO_4,_ and evaporated to yield cinnamic acid **30** as a white solid.

#### 3.1.5 Analytical and Spectroscopic Data for the Cinnamic Acids 11-20 are Given in the Supplementary Material

Acids **11-20** have already been documented in the literature, and their structure was confirmed *via* ^1^H NMR and LC-MS analysis compared to the corresponding bibliographic data [39-46].

#### 3.1.6 General Procedure for the Synthesis of the Amino Acid Cinnamates (11a-27a, 29a-30a, 11b-27b, 29b-30b, 11c-27c, 29c-30c)

**Scheme 3:**
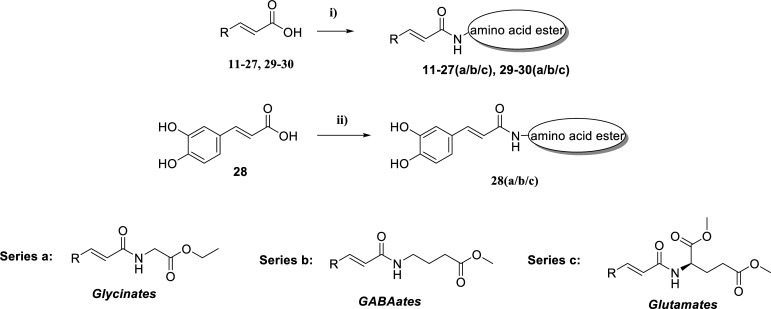
Reagents and conditions: i) 1^st^ step: corresponding cinnamic acid (1 equiv.), HOBt hydrate (1.5 equiv.), EDCI*HCl (1.5 equiv.), dry DCM (10 mL), inert atmosphere, room temperature (r.t.), varying completion times; 2^nd^ step: respected amino acid ester hydrochloride (1 equiv.) (glycine ethyl ester hydrochloride for compounds of series a, methyl gamma amino butyrate hydrochloride for compounds of series b, dimethyl L-glutamate hydrochloride for compounds of series c), DIPEA (1 equiv.), inert atmosphere, r.t. varying completion times; ii) 1st step: trans-caffeic acid (1.0 equiv.), HOBt hydrate (1.0 equiv.), EDCI HCl (1.0 equiv.), DMF (2 mL), 0°C, 10 min. 2^nd^ step: amino acid ester hydrochloride (1.0 equiv.), DIPEA (1.0 equiv.), 0°C to r.t., overnight.

The synthesis of the final products is given in Scheme **3**. In a round-bottomed flask were consequently added, dry DCM (10 mL), the appropriate acid (1.0 equiv.), HOBt*H_2_O (1.5 equiv.) and EDCI*HCl (1.5 equiv.). The mixture was stirred at room temperature under an inert atmosphere (N_2_ or Ar balloon) until the reaction was completed (TLC monitoring) [47]. The appropriate amino acid ester hydrochloride salt was added (1.0 equiv.), and an equimolar amount of DIPEA was added to the intermediate product. The reaction mixture was poured into a separating funnel and washed with water (3 x 15 mL); the organic layer was dried over Na_2_SO_4_, filtered, and evaporated to dryness. The crude product was subjected to column chromatography or was recrystallized from the appropriate solvent, as described in the given analytical, detailed data of each compound (see supplementary material). Analytical and spectroscopic data are also given in the supplemental material section. For the compounds already described in the literature, the ^1^H-NMR and HRMS spectra were taken and compared to their bibliographic data [48-54].

#### 3.1.7 General Procedure for the Synthesis of the Cinnamates 28a, 28b, 28c

The synthesis of the final products is given in Scheme **3**. In a round-bottomed flask containing DMF (2 mL), trans-caffeic acid (100 mg, 0.556 mmol, 1.0 equiv.), HOBt*H_2_O (85 mg, 0.556 mmol, 1.0 equiv.) and EDCI HCl (106 mg, 0.556 mmol, 1.0 equiv.) were added. The mixture was stirred at 0°C for 10 minutes. Consequently, a solution of the amino acid ester salt (78 mg, 0.556 mmol, 1.0 equiv.) and DIPEA (0.121 mL, 0.556 mol, 1.0 equiv.) in DCM (3 mL) was added. The mixture was stirred for 1 hour at 0°C and then overnight at rt until the reaction was completed (TLC monitoring) [55]. The mixture was poured into a separating funnel containing 10 mL of a 5% w/v NaHCO_3_ solution, and the aqueous phase was extracted with ethyl acetate (2 x 50 mL). The organic layers were collected, washed with brine (1 x 50 mL), dried over Na_2_SO_4_, and evaporated. The crude product was subjected to column chromatography using petroleum spirit - ethyl acetate mixtures as eluents or was recrystallized from the appropriate solvent, as described in the detailed analytical data of each compound (see supplementary material).

### 3.2 Biological Assays

The *in vitro* assays were performed at a concentration of 100 µM (a 10 mM stock solution in 0.1% DMSO in an appropriate buffer was used, from which several dilutions were made to determine IC_50_ values), at least in triplicate. The standard deviation of the absorbance was less than 10% of the mean.

IC_50_ determinations, OriginPro 8 was used to determine IC_50_ values from the sigmoidal line fitted to a log (concentration) graph against the average percentage inhibition from two independent experiments with at least 6 different concentrations. The SEM was the calculated standard error in the IC_50_ value of the fitted line.

Statistical comparisons were made using the Student T-test. A statistically significant difference was defined as *p* < 0.05.

#### 3.2.1 Inhibition of Linoleic Acid Peroxidation

Our group evaluated the *in vitro* study as reported previously [56]. Ten 16 mM sodium linoleate solution microliters were added to the UV cuvette containing 93 µL of a 0.05 M phosphate buffer, pH 7.4, and 10 µL of the tested compounds (final concentration 100 µM). The oxidation reaction started at 37o C under air by adding 50 µL of the free radical initiator AAPH (40 mM). Oxidation was carried out and monitored at 234 nm. DMSO under the same conditions was used as a negative control. Trolox was used as the appropriate standard (positive control). The results are given in Table **S1** in the supplementary material.

For compounds **12**, **18**, **21**, **22** and **23** our biological values are included taken from our bibliographic data [23, 57, 58].

#### 3.2.2 Soybean Lipoxygenase Inhibition Study

Our group evaluated an *in vitro* study as reported previously [42]. The tested hybrids were incubated at room temperature with sodium linoleate (100 µM) and 200 µL of enzyme solution (1/9 X 10^-4^ *w/v* in saline). Sodium linoleate is converted to 13-hydroperoxylinoleic acid, absorbed at 234 nm. Nor-dihydroguaeretic acid NDGA (IC_50_ = 0.45 µM) was used as a standard (positive control). Different concentrations were used for the determination of IC_50_. A blank determination was used as a negative control. The results are given in Table **S1** in the supplementary material.

#### 3.2.3 Cyclooxygenase -2 Inhibition Study

The *in vitro* study of cyclooxygenase (COX) activity [59] was determined by using arachidonic acid (AA) as the substrate and N,N,N’,N’-tetramethylphenylenediamine (TMPD) as the co-substrate. The reaction mixture (1 mL) contained 0.75 mM heme, 128 mM TMPD, 80 mM AA, and 1.5 mg enzyme in 0.1 M Tris/HCl (pH 8.5). The oxidation of the substrate was measured at room temperature by monitoring the absorbance increases at 611 nm. The absorption due to the spontaneous oxidation of TMPD was subtracted from the initial rate of oxidation observed in the presence of AA. The inhibition of the compounds was determined after preincubation for 6 min with the enzyme in the presence of heme and TMPD, and the reaction was started by adding AA. A blank determination was used first to serve as a negative control. Indomethacin was used as a reference COX-2 inhibitor (positive control). The results are given in Table **S1** in the supplementary material.

#### 3.2.4 Albumin Denaturation Studies

The synthesized compounds were evaluated for the inhibition of albumin denaturation [60]. Test tubes containing 1 mL of albumin solution (1% *w/v* in phosphate buffer pH=7.4), buffer, and 20 μL of the tested compounds dissolved in DMSO are incubated at 37°C for 15 minutes (final concentration 100 µM). The incubation follows at 60°C for another 15 minutes to induce albumin denaturation. The samples are cooled to room temperature. Their absorbance was measured at 660 nm. DMSO was used as a blank solution and acetylsalicylic acid as a reference compound. The results are given in Table **S1** in the supplementary material.

## 4 RESULTS AND DISCUSSION

### 4.1 Chemistry

Our synthetic work was based on a library of cinnamic amide hybrids with glycine, γ- aminobutyric acid (GABA), and L - glutamic acid subjected to docking studies. The most convenient method for the preparation of cinnamic acid derivatives (**11-20**) was proved to be the reaction of the appropriate benzaldehyde with malonic acid in pyridine solution to give the corresponding cinnamic acids in very good yields (70% to 100%) (Scheme **2**). The synthesis of (E)-3-(2,2-dimethylbenzo[d][1,3]dioxol-5-yl)acrylic acid (**29**) was performed *via* a three-step procedure entailing i) the reaction of trans-caffeic acid with methanol to yield the corresponding methyl ester quantitatively, ii) the reaction of the ester with 2,2-dimethoxypropane to give the corresponding acetonide in high yield (86%) and finally iii) the hydrolysis of the ester moiety with lithium hydroxide to give acid 29 in moderate yield (69%) (Scheme **2**).

The synthesis of (E)-3-(4-acetoxyphenyl) acrylic acid (**30**) proceeded quantitatively *via* a one-step procedure entailing the reaction of trans-p-coumaric acid with acetic anhydride to yield the final product (Scheme **2**).

The synthesis of the amides followed a two-step reaction with i) 1-hydroxybenzotriazole monohydrate (HOBt*H_2_O) and 1-ethyl-3-(3-dimethylaminopropyl) carbodiimide hydrochloride (EDCI*HCl) in dry dichloromethane (DCM) and ii) the corresponding amino acid ester hydrochloride salt in the presence of N,N-diisopropyl-N-ethylamine (DIPEA) (Scheme **3**). This synthetic route proved extremely useful since it does not use hazardous chemicals or extreme workup. The yields depended upon the amino acid ester used. Thus, we noticed that the GABA methyl ester derivatives showed higher yields (46 to 100%), followed by the Glycinates (47 to 99%) and the Glutamates (44 to 99%).

The structure of the synthesized compounds was confirmed spectrophotometrically: Infrared Spectroscopy (IR), Nuclear Magnetic Resonance (^1^H and ^13^C), High-Resolution Mass Spectrometry (HRMS), and optical activity measurements. The IR spectra of compounds revealed an absorption band at 1680-1660 cm^-1^, characteristic of the carbonyl group of the amide group of the hybrid and the ester group of the amino acid.

Regarding the compounds’ spectroscopic characteristics, in the ^1^H-NMR spectra, all the characteristic peaks were observed. The vinyl protons of the cinnamoyl moiety were recorded at around 7.60 and 6.50 ppm with a *J*-coupling constant of 15.6 Hz, indicative of the trans conformation. The amide proton was recorded as a broad singlet peak at around 6.00 ppm in CDCl_3_ or a triplet peak at about 8.50 ppm in DMSO-d_6_. In the ^13^C-NMR spectra, the amide and ester carbons were recorded at approximately 170 and 165 ppm, respectively. In the HRMS spectra, [M+H]^+^ and [M+Na]^+^ were recorded as the main ions, as described in the spectral properties of the compounds. The optical activity was calculated in methanol solution for all the newly synthesized glutamate hybrids. For compound **11c**, the optical activity could not be taken due to limited solubility in methanol. The physicochemical properties of the novel derivatives are given in the experimental section (see Supplementary material).

### 4.2 Drug-likeness *In silico*

Nowadays, the definition of a drug's “ideal” profile is analyzed chemometrically. The synthesized hybrids were analyzed *in silico,* and their physicochemical properties were determined to define their drug-likeness. To conduct these calculations, the online tool Molinspiration was used [33]. The results are given in Table **S2** in the supplementary material.

Lipinski’s rule suggests poor absorption or permeation is related to more than 5 H-bond donors and 10 H-bond acceptors. Furthermore, molecular weight (MW) values> 500 and calculated log *P* value > 5 lead to poor absorption/ permeability. It is concluded that none of the synthesized compounds violate Lipinski’s rule of five. Thus, all are considered as pharmacochemical entities.

A Central Nervous System (CNS) - active drug has to be BBB-permeant (blood-brain barrier) and devoid of P_gp_ (P glycoprotein) substrate activity (responsible for the main efflux from the brain). The designed molecules were subjected to *in silico* determination of their BBB penetration and activity as P_gp_ substrates using the online tools SwissADME, PreADMET, and Molsoft [61-63]. The results (see Table **S3** in the supplementary material) show that only few derivatives are considered as BBB permeant (compounds **11a, 12a, 13a, 14a, 15a, 16a, 17a, 22a, 23a, 27a, 29a, 11b, 12b, 13b, 14b, 15b, 16b, 17b, 22b, 23b, 27b, 29b**) while none of them acts as substrate for the P-glycoprotein efflux.

Several compounds may act as P-glycoprotein inhibitors (compounds **11a**, **13a**, **11b**, **12b**, **13b**, **17b**, **29b**, **11c**, **13c**, **14c**, **15c**, **17c**, **18c**, **19c**, **23c**, **30c**) (see Table **S2** in the supplementary material). Thus, they might affect the activity of several co-administered CNS-active agents. Although their permeability is considered negative, their hybrid structure (containing an amino acid scaffold) could permit their penetration actively - *via* different routes, *e.g*., with the help of alpha-amino acid transporters. We cannot predict alternative penetrations due to the lack of applicable specific computational platforms.

To study the compounds’ bioavailability, their gastrointestinal absorption and binding to plasma proteins were predicted using the PreADMET tool (see Table **S4** in the supplementary material) [62]. The *in silico* results show that all the designed molecules are highly absorbed after *per os* administration and exhibit medium to high binding to plasma proteins (above 50%).

Metabolism mainly affects drugs’ efficacy. The contemporary approach is to implement computational tools to predict a designed drug's metabolism safely and fast without *in vitro/ in vivo* tests and financial costs. P_450_ is the main enzyme family implicated in drug metabolism, accounting for approximately 40% [64] of the human body's overall metabolism and drug-drug interactions [65]. Thus, the synthesized compounds were subjected to an *in silico* study using the online tool GLORYx [66, 67], which predicts both Phase I and Phase II metabolism in humans (see Table **S5** in the supplementary material). In Table **S5**, the corresponding metabolites (in order of possibility 1^st^, 2^nd^, 3^rd^) of the compounds listed in the first column, are given within the columns.

The interaction of a drug with a P_450_ isoenzyme is almost inevitable. Studying and predicting the possible drug-P_450_ interaction is crucial in the drug design. This interaction will affect the activity of other co-administered drugs metabolized by P_450_ isoenzymes. Thus, the designed molecules were examined for the probability of acting as CYP1A2, CYP2C19, CYP2C9, CYP2D6, and CYP3A4 inhibitors using the online tool CypRules [68]. From the obtained data (see Table **S6** in the supplementary material), none of the designed hybrids showed any P450 inhibitory activity.

Contemporary drug development practice is asking *a priori* the evaluation of adverse effects/ toxicity using *in silico* tools. Since many drugs are metabolized in the liver, hepatotoxicity is the main adverse effect, which is of high importance in early-stage drug development. To predict the possible hepatotoxicity of the synthesized molecules, we analyzed them using the online tool LiverTox Workspace [69], which prioritizes the evaluated molecules' interactions with certain transporters associated with liver toxicity (see Tables **S7** and **S8** in the supplementary material). From the obtained data, it seems that only compounds **11b**, **17b**, **19b**, and **20b** might cause hyperbilirubinemia, while compounds **12a**, **24a**, **27a**, **29a**, **30a**, and **12b** are not considered to cause cholestasis. Compound **21b** showed no interaction with any of the studied transporters, while compound **11c** interacted with the highest number of transporters - 7.

### 4.3 Biological Assays

The hybrids' ability to act as multitarget agents was studied in the present investigation. Considering the multifactorial character of inflammation, the new compounds were designed and evaluated *in vitro* as i) soybean lipoxygenase (LOX), ii) cyclooxygenase-2 (COX-2), and iii) lipid peroxidation inhibitors. Reactive Oxygen Species (ROS) are highly reactive molecules derived mainly from the mitochondrial electron transport chain and other pathways. Among them are the respiratory burst taking place in activated phagocytes, ionizing radiation’s damaging effect on components of cell membranes, and as by-products of several cellular enzymes, including NADPH oxidases (NOX), xanthine oxidase (XO), and uncoupled endothelial nitric oxide synthase (eNOS) [37].

ROS are related to metabolic disorders such as insulin resistance, diabetes mellitus, obesity, and chronic inflammation. Some ROS are characterized as highly toxic. Their extreme reactivity and the tendency to induce chain reactions lead to pathological processes. Earlier studies have shown a significant enhancement of lipid peroxidation in the brain of Alzheimer’s patients. Thus, antioxidants inhibiting brain lipid peroxidation could aid their treatment and prove promising in preventing and/or treating ROS-related diseases. Epidemiological studies revealed the link between reactive oxygen species and inflammation.

We used the water-soluble 2, 2’-azobis(2-amidinopropane) hydrochloride (AAPH) to generate *in vitro* peroxyl radicals through spontaneous thermal decomposition [23], which resembles cellular lipid peroxidation due to the activity of the undertaken radicals. The anti-lipid peroxidation activities were compared to a well-known antioxidant, *i.e*., Trolox (Table **S1** in the supplementary material). Glutamate derivatives seem more potent than lipid peroxidation inhibitors, followed by glycinates and GABA derivatives. Among the glycinate hybrids, compound **18a** was the most potent inhibitor (99%), while among the GABA hybrids, compound **23b** was the most active (78%). From the glutamate derivatives, compound **11c** proved to be the most potent (92%). In general, the anti-lipid peroxidation ranged from 8-99%. Except for acids **11**, **12**, **17**, **19**, **21**, **22**, **24**, **25**, and **28**, which present high antioxidant activities (on the high % level), the other cinnamic acids present low or medium activity. Lipophilicity as theoretically calculated values (logP) does not seem to influence the lipid peroxidation inhibition.

A perusal of the % anti-lipid peroxidation within the subgroups shows that conjugated double bonds in the cinnamic scaffold, as in compound 23a (85%), increase the antioxidant activity for the glycine hybrids. In contrast, as in derivative **22a**, the absence lowers it (5%). Among the glycinate hybrids, p-coumaric and caffeic analogs are more potent than the corresponding simple cinnamic acid **22a** (5%), **25a** (39%), and **28a** (47%). Methylation of the phenolic OH groups enhances the activity, *e.g*., derivatives **24a** (82%), 18a (99%), and **19a (89%).** The presence of the dioxole ring in the hybrids increases their antioxidant activity compared to the activity of the hybrids derived from the simple cinnamoyl scaffold (compounds **22a** (5%), **20a** (70%), and **29a** (80%)). The replacement of the phenyl group by a condensed ring as a naphthyl ring or by a heteroaromatic as thienyl decreases or vanishes activity (compounds **22a** (5%) and 1**7a** (n.a.)).

Regarding the GABA hybrids, the presence of hydroxyl groups on the phenyl ring (coumaric/ caffeic) and their methylation leads to higher inhibition of lipid peroxidation. Thus, the insertion of a hydroxyl group in **22b** (24%) gives **25b** with an increase in activity (66%), whereas the results from the caffeic hybrid with the two hydroxyl groups give **28b** (58%). Methylation of the two hydroxyl groups leads to **18b** (75%). The benzo[d][1,3]dioxole ring derivatives presented lower activity - compounds **29b** (n.a.), **20b** (9%). Herein also, the presence of a second conjugated double bond positively influences the activity (compounds **22b** (24%) *versus* **23b** (78%).

For the *L*-glutamates, coumaric and caffeic hybrids **25c** and **28c** exhibit higher activity compared to the cinnamic hybrid (compounds **22c** (39%) *versus* **25c** (78%) and **28c** (87%). The presence of a dioxole-fused ring is followed by lower activity [derivatives **20c** (20%) and **29c** (27%)] since the aromatic hydroxyl groups have been abstracted.

All the novel hybrids were studied for their ability to inhibit soybean LOX. The role of ALOX-5 in the development of neurodegenerative diseases has already been described [5-7]. Developing novel and potent ALOX-5 inhibitors is very important for treating neurodegenerative diseases. LOX inhibitors bearing an antioxidant profile could be expected to offer protection in inflammatory conditions and lead to potentially effective drugs.

Due to the limited work on sufficiently purified human ALOX-5 isoenzyme, most of the published research was performed on the soybean homolog. Several reports showed a qualitative correlation between these two enzymes [70, 71]. This study used the soybean isoenzyme LOX-1, which exerts maximal activity at pH 9.0 [72]. The results are depicted in Table **S1** at the supplementary material and expressed as % inhibition at 100 μΜ concentration or IC_50_ values. Nor-dihydroguaretic acid (NDGA) was used as a reference compound. IC_50_ values could not be determined for all compounds. The compounds presented low to moderate inhibitory activity. The most potent derivative seems to be **28c,** the hybrid of caffeic acid with glutamate and IC_50_ = 8.5 μΜ. The anti-LOX activity of compound 20c could be correlated with the antioxidant ability due to the presence of the phenolic hydroxyl groups, which are not sterically hindered. Thus, this molecule could act as an antioxidant. In descending order, the most potent hybrids were **28c** (IC_50_ = 8.5 μΜ), **18c** (IC_50_ = 57.5 μΜ), **15a** (IC_50_ = 57.5 μΜ), **12c** (IC_50_ = 60 μΜ), **18a** (IC_50_ = 62.5 μΜ) and **25c** (IC_50_ = 63 μΜ). In terms of structure, the hybrids mentioned above were derived from glycinate and glutamate groups. Among the potent LOX inhibitors, the GABA hybrids are less important except for only two analogs: **20b** (67.5 μΜ) and **24b** (100 μΜ).

The role of COX-2 in neurodegenerative diseases has been reported [8-11], and COX-2 inhibition is considered of high importance as a therapeutic tool. The designed derivatives were tested for their COX-2 inhibition activity [73] on the ovine COX-2 enzyme. The results are shown in Table **S1** at the supplementary material and expressed as % inhibition at 100 μM or IC_50_ values. Indomethacin was used as a reference compound. The most potent derivatives were hybrids **22a** (5 μΜ) and **11a** (6 μΜ). Both are glycinate hybrids, whereas 11a has a larger substituent (bromobenzyloxy group) than the simple cinnamic acid phenyl ring **22**. It should be noticed that the latter presents only 4% COX-2 inhibition, whereas hybrid **22a** is changed to a highly potent molecule through hybridization. Similar IC_50_ values are taken for **16a, 17c, 18b, 20c**, and **21a, 23b.** The attempt to correlate lipophilicity and/or molecular volume with the inhibitory activity did not yield fruitful results. The two most active compounds present almost identical IC_40_ values, suggesting that both might bind in the same manner at the protein active site.

The experimental results indicate that some tested hybrids present dual inhibiting properties on both enzymes. Among them are **12a, 15c, 17c, 20c, 23c, 25b** and **28a.** The most potent combination is given by glutamates **17c** and **23c.** It is known that dual LOX/COX inhibitors are considered potential new drugs to treat inflammatory diseases as well as neurodegeneration disorders. They act by blocking the formation of both prostaglandins and leukotrienes but do not affect lipoxin formation. Such combined inhibition avoids some disadvantages of selective COX-2 inhibitors affecting the gastrointestinal mucosa. Frequent inhibition of either cyclooxygenase or lipoxygenase enzyme switches the metabolism of arachidonic acid from one to another with serious disorders. Thus, a need to develop novel, effective, safe anti-inflammatory agents that can inhibit cyclooxygenase and lipoxygenase pathways has emerged [74].

Protein denaturation has correlated well with the inflammatory response, leading to various inflammatory diseases [75, 76]. It is known [77] that tissue injury during life might refer to the denaturation of cells' protein constituents or intercellular substances. Hence, the ability of a substance to inhibit the denaturation of protein signifies apparent potential for anti-inflammatory activity. Many anti-inflammatory drugs can inhibit the heat-coagulation of serum albumin [78]. Thus, the inhibition of albumin heat-coagulation activity of the designed molecules was examined *in vitro* [60] as a measure of their anti-inflammatory activity. The experimental results were compared to the standard drug, acetylsalicylic acid. The results showed that only derivatives **28b, 28c, 29a,** and **29c** exhibited inhibitory activity, with compound **29c** being the most potent (75%), while acetylsalicylic acid showed 31.2% inhibition. The results are given in Table **S1** in the supplementary material.

### 4.4 Molecular Docking Studies

#### 4.4.1 Binding Mode of Hybrid 28c in Soybean Lipoxygenase 1

The docking studies of the most active derivative **28c** on soybean lipoxygenase-1 (PDB ID: 3PZW) with IC_50_ = 8.5 µM are by the biological protocol. Lipoxygenases are dioxygenases containing a ‘non-heme’ iron per molecule, catalyzing the oxygenation of free and esterified polyunsaturated fatty acids containing a (1Z, 4Z)-penta-1,4-diene system to the corresponding hydroperoxy derivatives. Lately, it has been investigated that lipoxygenases are present apart from the substrate-binding site (iron-binding site) and potential allosteric binding sites [79, 80]. Thus, compound **28c** has been studied for its binding mode to the active site and the whole protein to encompass all the allosteric sites. Compound **28c** binds allosterically, thus not entering the enzyme’s active cavity. Previous publications confirm these findings [81-83].

Compound **28c** presented an AutoDockVina binding score of -7.9 kcal/mol on SLOX-1. It seems that **28c** exerts hydrophobic interactions with Phe143, Val520, and Lys526. Alternate hydrogen bonds are also developed between a) Arg141, Ile142, and Arg182 with the p-OH group of caffeic acid and b) Asp243 with the m-OH group of caffeic acid. Finally, the binding was reinforced with salt bridges with residues Lys526 and Arg767. The binding mode of compound **28c** is depicted in Fig. (**S1**) (see supplementary material).

#### 4.4.2 Binding Modes of Hybrids 11a and 22a in COX-2

Compounds **11a** and **22a** presented binding scores of -8.4 kcal/mol and -7.6 kcal/mol on ovine COX-2, respectively. Compound **11a** interacts hydrophobically with residues Val318, Leu321, Trp354, Pro483 and Val492. Hydrogen bonds are developed between a) His58 and the compound’s NH and b) Arg482 with the compound’s oxygen of the amide’s group. Furthermore, the complex is stabilized by salt bridges with residue His58. Compound **22a** interacts hydrophobically with residues Leu321, Leu353, Tyr354, Trp356, Val492 and Ala496, developing salt bridges with residues His58 and Arg482. The binding modes of compounds **11a** and **22a** are depicted in Figs. (**S2** and **S3**), respectively (see supplementary material).

## CONCLUSION

A series of amino acid hybrids with cinnamic acids were designed following modeling and *in silico* drug-likeness studies. The final products were synthesized in median to high yields. Compounds **11a** and **22a** are hydrophobically bound to COX-2, followed by salt bridges and hydrogen bonds, while compound **28c** is bound to SLOX-1 by several hydrophobic interactions and hydrogen bonds. A thorough *in silico* study of the molecules’ pharmacokinetic properties showed that all are considered active following *per os* administration since none violates Lipinski’s rule of five and the GI tract readily absorbs all. A few molecules could easily penetrate the blood-brain barrier, while their hybrid structure could help the other hybrids to cross it *via* alternative routes. None of the designed molecules were substrates for the P-glycoprotein.

The metabolism of the designed molecules is diverse, depending on substituting the cinnamoyl core. None of the designed molecules acts as a P_450_ isoenzyme inhibitor. Compound **21b** exerted the safest profile regarding interaction with selected transporters, while compound **24a** was not found to induce liver injury, cholestasis, or hyperbilirubinemia.

All the novel hybrids were evaluated for their ability to inhibit LOX and COX-2 implicated in inflammation. The *in vitro* results were by the docking simulations that were performed. The compounds exhibited LOX inhibition with IC_50_ values in the low μM region. Compounds **18a, 23b**, and **11c** exhibited the highest antioxidant activity (99%, 78%, and 92%, respectively. Compound **28c** is a selective SLOX-1 inhibitor with an IC_50_ value of 8.5 μM. Compounds **11a** and **22a** act as selective COX-2 inhibitors with IC_50_ values of 6 and 5 μM, respectively. Compounds **11a** and **22a** can be characterized as the most drug-like candidates since the combination of their high COX-2 inhibitory activity, their physicochemical properties, and their structural characteristics can ensure i) their transfer across the BBB and ii) their effectiveness and low toxicity risk.

Compound **28c** can also be considered a drug-like molecule due to its high anti-LOX activity combined with no violation of Lipinski’s rule of five. Although the theoretical BBB penetration calculation is unfavorable, its hybrid amino acid structure could facilitate its penetration *via* another mode (*e.g*., active transport through the amino acid transporters). However, hybrids **15c** and **17c** presented an interesting dual inhibition mode of action against both enzymes and have been identified as novel potent pleiotropic agents. Compound **29c** showed the highest anti-inflammatory activity since it inhibited albumin denaturation by 75%.

Compound **28c** can be considered a lead hybrid for selective SLOX-1 inhibition, whereas compounds **11a** and **22a** are lead structures for COX-2 inhibition. Further investigation is in progress regarding the influence of the above hybrids on neuro-inflammation.

## Data Availability

Not applicable.

## LIST OF ABBREVIATIONS

AAPH: 2,2'-Azobis(2-amidinopropane) dihydrochloride
ACPYPE: AnteChamber PYthon Parser interface
ADMET: Administration, Distribution, Metabolism, and Toxicity
ALOX-5: Lipoxygenase - 5
AMPA: a-amino-3-hydroxy-5-methyl-4-isoxazolepropionic acid
COX-2: Cyclooxygenase - 2
DIPEA: N,N-Diisopropylethylamine
DMAP: 4-Dimethylaminopyridine
DMP: 2,2-dimethoxypropane
EDCI*HCl: 1-Ethyl-3-(3-dimethylaminopropyl) carbodiimide hydrochlroide
GABA: Gamma Amino Butyric Acid
HOBt*H_2_O: 1-Hydroxybenzotriazole Hydrate
iGluR: Ionotropic Glutamate Receptor
NDGA: Nordihydroguaiaretic Acid
NMDA: N-methyl-D-aspartic Acid
PGE2: Prostaglandin E2
Ptsa: p-toluenesulfonic Acid
PUFAs: Polyunsaturated Fatty Acids
ROS: Reactive Oxygen Species
TMPD: N,N,N,N-tetramethylphenylenediamine
