# Supplementary material for: Pharmacochemical Study of Multitarget Amino Acids’ Hybrids: Design, Synthesis, In vitro, and In silico Studies
Source: Med Chem. 2024 Feb 9;20(7):709–20. doi: 10.2174/0115734064279653240125081042 (PMC11348465; doi:10.2174/0115734064279653240125081042)
Supplement: Supplementary file 1 [file MC-20-709_SD1.pdf]

Supplementary Materials

Pharmacochemical Study of Multitarget Amino Acids' Hybrids. Design, Synthesis, *In vitro* and *In silico* Studies

Ioannis Fotopoulos[1], Eleni Pontiki[1] and Dimitra Hadjipavlou – Litina[1*]

[1]*Department of Pharmaceutical Chemistry, School of Pharmacy, Faculty of Health Sciences, Aristotle University of Thessaloniki, Thessaloniki, 54124, Greece*

1. TABLES OF MAIN TEXT

Table 1 - % Anti-lipid peroxidation (AAPH-induced), in-vitro inhibition of soybean lipoxygenase, ovine cyclooxygenase-2 (COX)-2, and Albumin denaturation inhibitory activity.

Compound	Structure	logP [33]	AAPH inhibition (%, at 100 μM)	LOX inhibition (% or IC_{50} values μM)	COX-2 inhibition (% or IC_{50} values)	Albumin denaturation (%, at 100 μM)
**11**	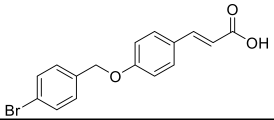	4.37	84 [39]	100 μM	17%	n.t.
**11a**	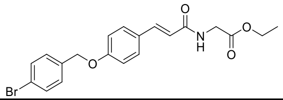	4.47	38	n.a.	6 μM	n.a.
**11b**	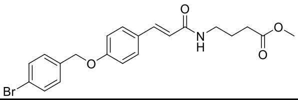	4.33	19	30%	n.a.	n.a.
**11c**	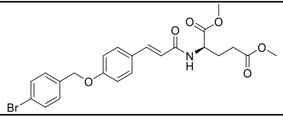	4.17	92	n.a.	n.a.	n.a.
**12**	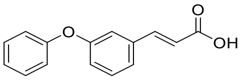	3.64	84 [23]	66 μM [23]	39%	n.t.
**12a**	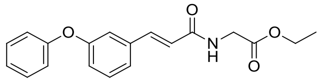	3.74	48	36%	55 μM	n.a.
**12b**	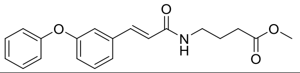	3.60	n.a.	n.a.	n.a.	n.a.
**12c**	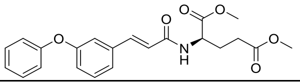	3.44	65	60 μM	n.a.	n.a.
**13**	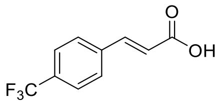	3.44	37	42%	32%	n.t.
**13a**	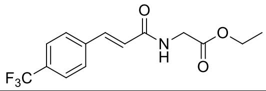	2.81	8	31%	n.a.	n.a.
**13b**	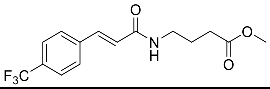	2.90	n.a.	n.a.	n.a.	n.a.

1573-4064/24 © 2024 Bentham Science Publishers

**13c**	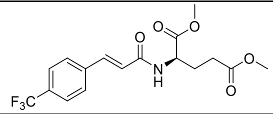	2.76	37	80 μM	n.a.	n.a.
**14**	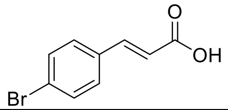	2.61	13	n.a.	100 μM	n.t.
**14a**	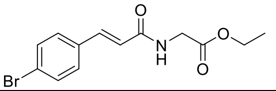	2.72	69	n.a.	65.5 μM	n.a.
**14b**	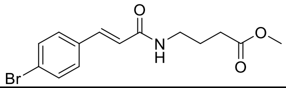	2.82	11	12%	n.a.	n.a.
**14c**	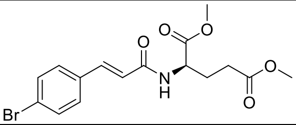	2.67	38	75 μM	n.a.	n.a.
**15**	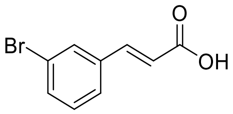	2.52	n.a.	n.a.	n.a.	n.t.
**15a**	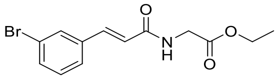	2.69	26	57.5 μM	n.a.	n.a.
**15b**	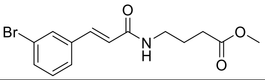	2.79	27	n.a.	43%	n.a.
**15c**	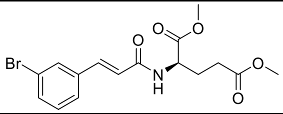	2.65	n.a.	75 μM	72.5μM	n.a.
**16**	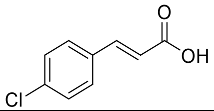	2.50	n.a.	n.a.	46%	n.t.
**16a**	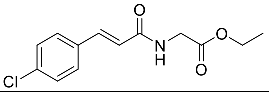	2.59	n.a.	n.a.	63 μM	n.a.
**16b**	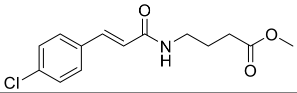	2.69	8	n.a.	92.5 μM	n.a.
**16c**	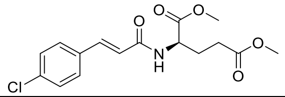	2.54	26	n.a.	n.a.	n.a.
**17**	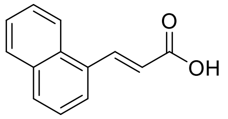	4.17	78	27.5 μM	n.a.	n.t.
**17a**	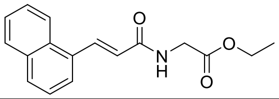	2.89	n.a.	23%	n.a.	n.a.
**17b**	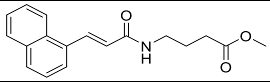	2.99	n.a.	n.a.	n.a.	n.a.
**17c**	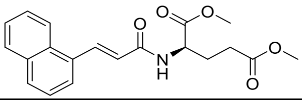	2.85	57	68 μM	62 μM	n.a.

**18**	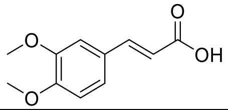	2.69	86 [23]	n.a. [23]	19%	n.t.
**18a**	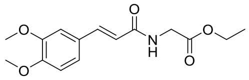	1.56	99	62.5 μM	14%	n.a.
**18b**	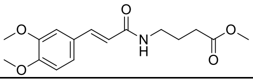	1.66	75	40%	61.5μM	n.a.
**18c**	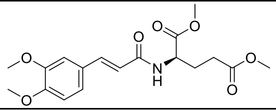	1.51	74	57.5 μM	n.a.	n.a.
**19**	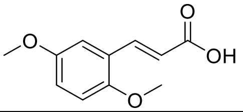	1.36	88	60 μM	28%	n.t.
**19a**	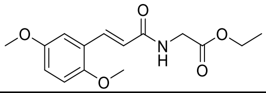	1.77	89	28%	n.a.	n.a.
**19b**	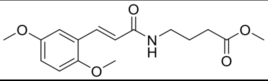	1.87	70	n.a.	20%	n.a.
**19c**	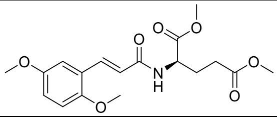	1.73	64	7%	n.a.	n.a.
**20**	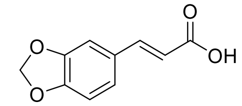	1.58	31	27%	n.a.	n.t.
**20a**	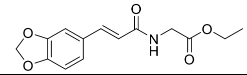	1.80	70	6%	75 μM	n.a.
**20b**	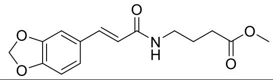	1.90	9	67.5μM	n.a.	n.a.
**20c**	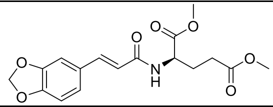	1.75	20	19%	61μM	n.a.
**21**	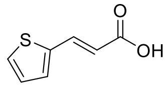	1.27	60 [57]	98 μM [57]	n.a.	n.t.
**21a**	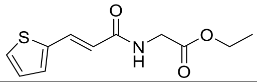	1.63	15	20%	55 μM	n.a.
**21b**	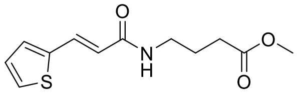	1.73	73	0%	38%	n.a.
**21c**	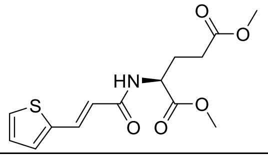	1.58	41	100 μM	n.a.	n.a.
**22**	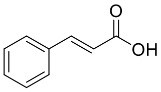	1.43	78 [23]	56μM [23]	4%	n.t.
**22a**	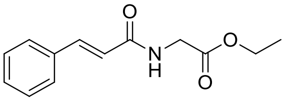	1.91	5	0%	5μM	n.a.

**22b**	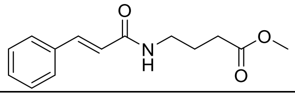	2.01	24	n.a.	61 μM	n.a.
**22c**	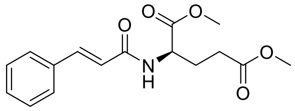	1.86	39	10%	n.a.	n.a.
**23**	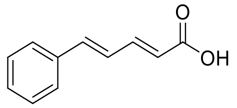	1.71	30 [57]	24% [58]	9%	n.t.
**23a**	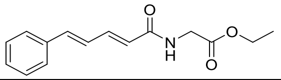	2.43	85	n.a.	35 μM	n.a.
**23b**	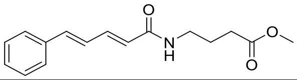	2.53	78	n.a.	55 μM	n.a.
**23c**	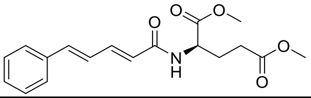	2.38	25	75 μM	47%	n.a.
**24**	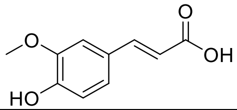	2.23	86	100 μM	14%	n.t.
**24a**	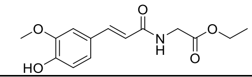	1.25	82	100 μM	n.a.	n.a.
**24b**	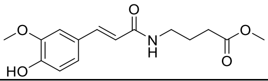	1.35	34	100 μM	n.a.	n.a.
**24c**	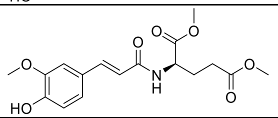	1.20	12	75μM	9%	n.a.
**25**	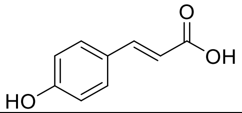	1.05	76	10 μM	62μM	n.t.
**25a**	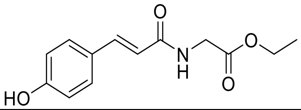	1.43	39	n.a.	n.a.	n.a.
**25b**	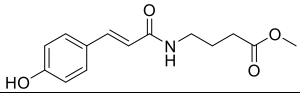	1.53	66	10%	61.5μM	n.a.
**25c**	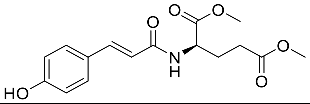	1.39	78	63 μM	n.a.	n.a.
**26**	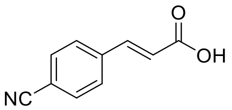	1.24	20 [57]	27%	65μM	n.t.
**26a**	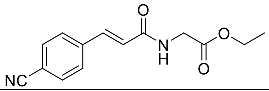	1.66	37	n.a.	77.5 μM	n.a.
**26b**	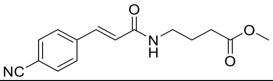	1.76	3	n.a.	32.5 μM	n.a.
**26c**	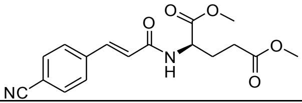	1.62	n.a.	10%	n.a.	n.a.

**27**	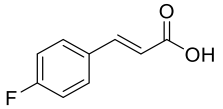	1.47	n.a.	n.a.	46%	n.t.
**27a**	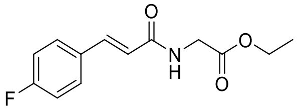	2.07	78	n.a.	n.a.	n.a.
**27b**	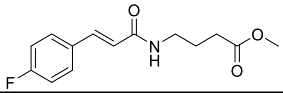	1.80	22	n.a.	75 μM	n.a.
**27c**	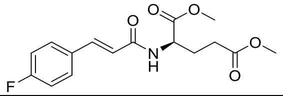	2.03	10	8%	72.5 μM	n.a.
**28**	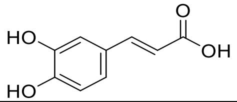	1.10	80	10 μM	6%	n.t.
**28a**	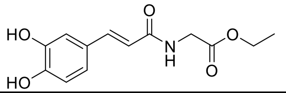	0.94	47	70 μM	17%	n.a.
**28b**	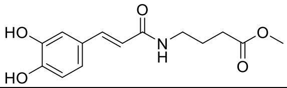	1.04	58	n.a.	100 μM	44
**28c**	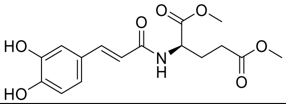	0.90	87	8,5 μM	19%	31
**29**	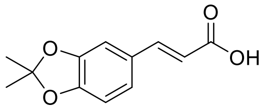	1.77	56	46	n.a.	n.t.
**29a**	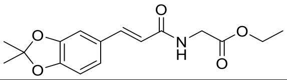	2.61	80	n.a.	38%	25
**29b**	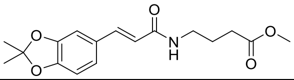	2.71	n.a.	n.a.	4%	n.a.
**29c**	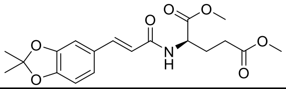	2.56	27	n.a.	n.a.	75
**30**	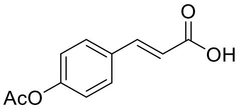	2.41	33	23%	n.a.	n.t.
**30a**	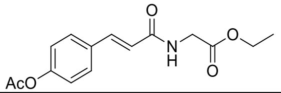	1.46	n.a.	n.a.	58 μM	n.a.
**30b**	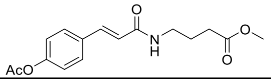	1.56	9	n.a.	50 μM	n.a.
**30c**	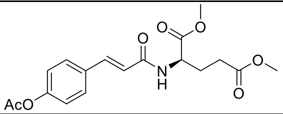	1.42	16	100 μM	6%	n.a.
**Trolox**	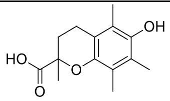		92	-	-	-
**NDGA**	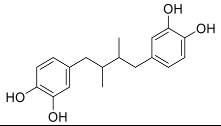		-	0.45 μM	-	-

**Indome thacin**	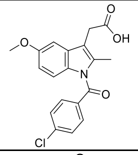		-	-	1.12 μM	-	
**Aspirin**	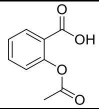		-	-	-	31.2	

Table 2 - Calculated physicochemical properties of the synthesized compounds, using the Molinspiration platform.

Compound	miLogP	TPSA	Natoms	MW	nON	nOHNH	Nviol	Rot	MV
**11a**	4.47	64.64	26	418.29	5	1	0	9	336.06
**12a**	3.74	64.64	24	325.36	5	1	0	8	301.37
**13a**	2.90	55.40	21	301.26	4	1	0	7	252.28
**14a**	2.82	55.40	18	312.16	4	1	0	6	238.86
**15a**	2.79	55.40	18	312.16	4	1	0	6	238.86
**16a**	2.69	55.40	18	267.71	4	1	0	6	234.51
**17a**	2.99	55.40	21	283.33	4	1	0	6	264.97
**18a**	1.66	73.87	21	293.32	6	1	0	8	272.07
**19a**	1.87	73.87	21	293.32	6	1	0	8	272.07
**20a**	1.90	73.87	20	277.28	6	1	0	6	244.91
**21a**	1.73	55.40	16	239.30	4	1	0	6	211.69
**22a**	2.01	55.40	17	233.27	4	1	0	6	220.98
**23a**	2.53	55.40	19	259.31	4	1	0	7	248.40
**24a**	1.35	84.86	20	279.29	6	2	0	7	254.54
**25a**	1.53	75.63	18	249.27	5	2	0	6	229.00
**26a**	1.76	79.19	19	258.28	5	1	0	6	237.84
**27a**	1.39	94.85	22	305.29	7	1	0	7	263.89
**28a**	1.04	95.86	19	265.26	6	3	0	6	237.01
**29a**	2.71	73.87	22	305.33	6	1	0	6	277.73
**30a**	1.56	81.71	21	291.30	6	1	0	8	265.51
**11b**	4.33	64.64	27	432.31	5	1	0	10	352.86
**12b**	3.60	64.64	25	339.39	5	1	0	9	318.17
**13b**	2.76	55.40	22	315.29	4	1	0	8	269.08
**14b**	2.67	55.40	19	326.19	4	1	0	7	255.67
**15b**	2.65	55.40	19	326.19	4	1	0	7	255.67
**16b**	2.54	55.40	19	281.74	4	1	0	7	251.32
**17b**	2.85	55.40	22	297.35	4	1	0	7	281.77
**18b**	1.51	73.87	22	307.35	6	1	0	9	288.87
**19b**	1.73	73.87	22	307.35	6	1	0	9	288.87
**20b**	1.75	73.87	21	291.30	6	1	0	7	261.71
**21b**	1.58	55.40	17	253.32	4	1	0	7	228.49
**22b**	1.86	55.40	18	247.29	4	1	0	7	237.78
**23b**	2.38	55.40	20	273.33	4	1	0	8	265.20
**24b**	1.20	84.86	21	293.32	6	2	0	8	271.34
**25b**	1.39	75.63	19	263.29	5	2	0	7	245.80
**26b**	1.62	79.19	20	272.30	5	1	0	7	254.64
**27b**	2.03	55.40	19	265.28	4	1	0	7	242.71
**28b**	0.90	95.86	20	279.29	6	3	0	7	253.82
**29b**	2.56	73.87	23	319.36	6	1	0	7	294.53
**30b**	1.42	81.71	22	305.33	6	1	0	9	282.31

**11c**	4.17	90.94	31	490.35	7	1	0	12	397.42
**12c**	3.44	90.94	29	397.43	7	1	0	11	362.73
**13c**	2.61	81.71	26	373.33	6	1	0	10	313.63
**14c**	2.52	81.71	23	384.23	6	1	0	9	300.22
**15c**	2.50	81.71	23	384.23	6	1	0	9	300.22
**16c**	2.39	81.71	23	339.77	6	1	0	9	295.87
**17c**	2.69	81.71	26	355.39	6	1	0	9	326.33
**18c**	1.36	100.18	26	365.38	8	1	0	11	333.43
**19c**	1.58	100.18	26	365.38	8	1	0	11	333.43
**20c**	1.60	100.18	25	349.34	8	1	0	9	306.26
**21c**	1.43	81.71	21	311.36	6	1	0	9	27305
**22c**	1.71	81.71	22	305.33	6	1	0	9	282.34
**23c**	2.23	81.71	24	331.37	6	1	0	10	309.75
**24c**	1.05	111.17	25	351.36	8	2	0	10	315.90
**25c**	1.24	101.94	23	321.33	7	2	0	9	290.35
**26c**	1.47	105.50	24	330.34	7	1	0	9	299.19
**27c**	1.88	81.71	23	323.32	6	1	0	9	287.27
**28c**	0.75	122.16	24	337.33	8	3	0	9	298.37
**29c**	2.41	100.18	27	377.39	8	1	0	9	339.09
**30c**	1.27	108.01	26	363.37	8	1	0	11	326.87

Table 3 - Retrieved data regarding the CNS permeability and Pgp interaction of the designed molecules.

**Compound**	**Structure**	**SwissADME**		**PreADMET**		**Molsoft**
		BBB permeant	P_{gp} substrate	BBB permeability[1]	P_{gp} inhibition	BBB score [5]
**11a**	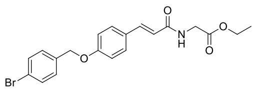	Yes	No	0.111	Inhibitor	4.21
**12a**	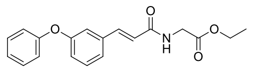	Yes	No	0.042	Non inhibitor	4.01
**13a**	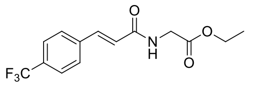	Yes	No	0.118	Inhibitor	4.21
**14a**	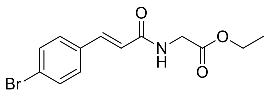	Yes	No	0.093	Non inhibitor	4.24
**15a**	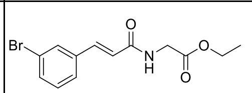	Yes	No	0.215	Non inhibitor	4.24

[1] Ratio of a substance's concentration in the brain over the substance's concentration in the blood.

**16a**	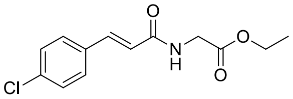	Yes	No	0.082	Non inhibitor	4.09
**17a**	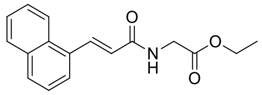	Yes	No	0.099	Non inhibitor	4.34
**18a**	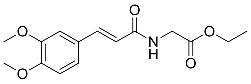	No	No	0.062	Non inhibitor	2.97
**19a**	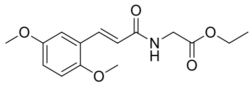	No	No	0.083	Non inhibitor	2.97
**20a**	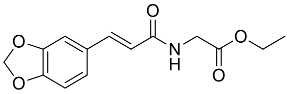	No	No	0.074	Non inhibitor	2.95
**21a**	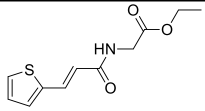	No	No	0.130	Non inhibitor	3.92
**22a**	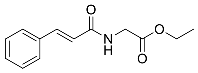	Yes	No	0.083	Non inhibitor	3.92
**23a**	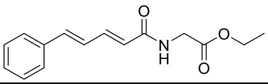	Yes	No	0.061	Non inhibitor	4.06
**24a**	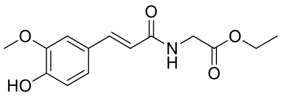	No	No	0.065	Non inhibitor	2.85
**25a**	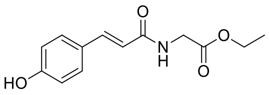	No	No	0.078	Non inhibitor	2.93
**26a**	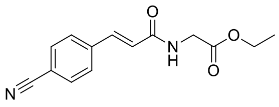	No	No	0.020	Non inhibitor	3.46
**27a**	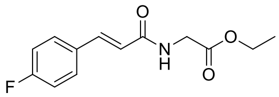	Yes	No	0.045	Non inhibitor	4.02
**28a**	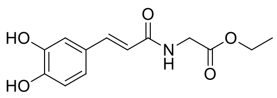	No	No	0.123	Non inhibitor	2.73

**29a**	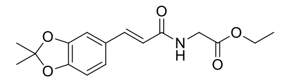	Yes	No	0.057	Non inhibitor	2.98
**30a**	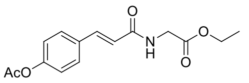	No	No	0.014	Non inhibitor	2.89
**11b**	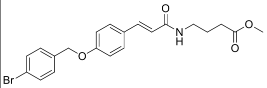	Yes	No	0.158	Inhibitor	4.21
**12b**	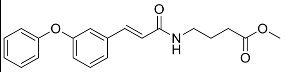	Yes	No	0.052	Inhibitor	4.03
**13b**	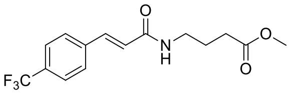	Yes	No	0.172	Inhibitor	4.24
**14b**	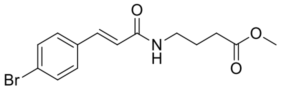	Yes	No	0.155	Non inhibitor	4.29
**15b**	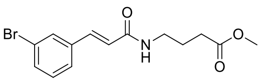	Yes	No	0.149	Non inhibitor	4.29
**16b**	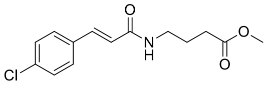	Yes	No	0.140	Non inhibitor	4.15
**17b**	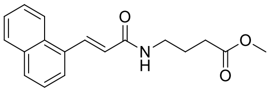	Yes	No	0.061	Inhibitor	4.37
**18b**	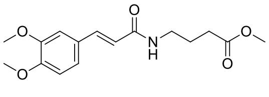	No	No	0.080	Non inhibitor	2.96
**19b**	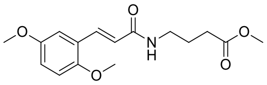	No	No	0.025	Non inhibitor	2.96
**20b**	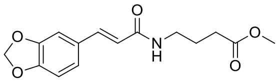	No	No	0.075	Non inhibitor	2.94
**21b**	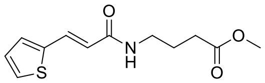	No	No	0.048	Non inhibitor	4.00

**22b**	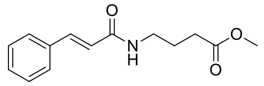	Yes	No	0.052	Non inhibitor	4.00
**23b**	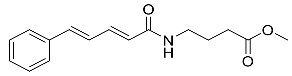	Yes	No	0.082	Non inhibitor	4.12
**24b**	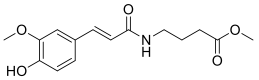	No	No	0.07	Non inhibitor	2.84
**25b**	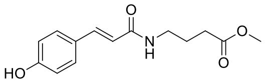	No	No	0.109	Non inhibitor	2.94
**26b**	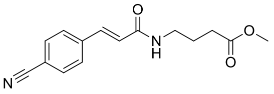	No	No	0.041	Non inhibitor	3.52
**27b**	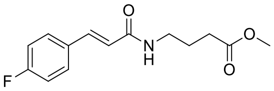	Yes	No	0.086	Non inhibitor	4.08
**28b**	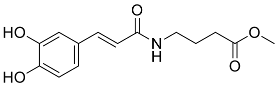	No	No	0.133	Non inhibitor	2.72
**29b**	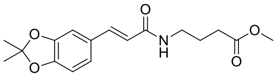	Yes	No	0.081	Inhibitor	3.40
**30b**	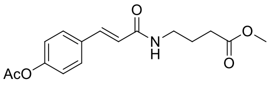	No	No	0.017	Non inhibitor	2.88
**11c**	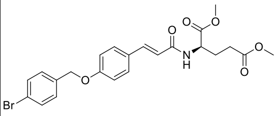	No	No	0.030	Inhibitor	2.68
**12c**	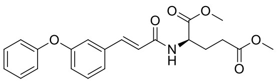	No	No	0.031	Non inhibitor	2.77
**13c**	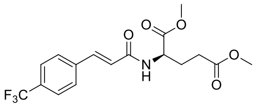	No	No	0.029	Inhibitor	2.79
**14c**	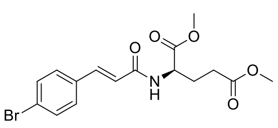	No	No	0.043	Inhibitor	2.86

**15c**	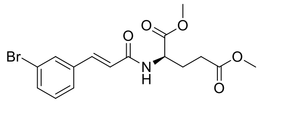	No	No	0.122	Inhibitor	2.86
**16c**	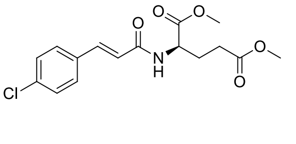	No	No	0.042	Non inhibitor	2.86
**17c**	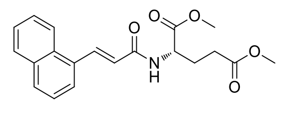	No	No	0.048	Inhibitor	2.97
**18c**	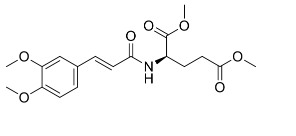	No	No	0.138	Inhibitor	2.57
**19c**	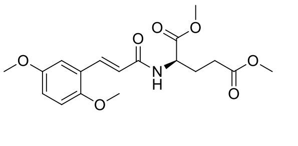	No	No	0.088	Inhibitor	2.57
**20c**	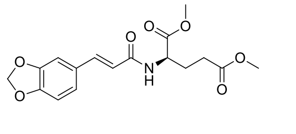	No	No	0.141	Non inhibitor	2.58
**21c**	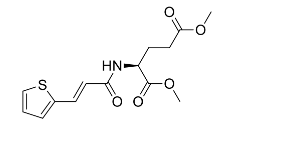	No	No	0.109	Non inhibitor	2.87
**22c**	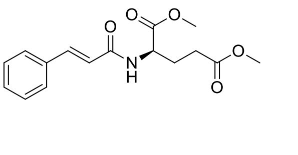	No	No	0.055	Non inhibitor	2.88
**23c**	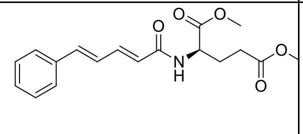	No	No	0.027	Inhibitor	2.84
**24c**	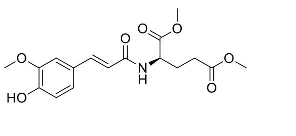	No	No	0.052	Non inhibitor	2.48
**25c**	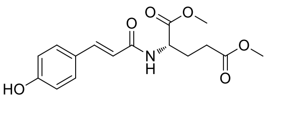	No	No	0.028	Non inhibitor	2.62
**26c**	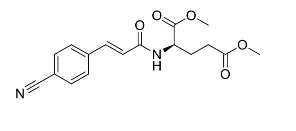	No	No	0.084	Non inhibitor	2.60

**27c**	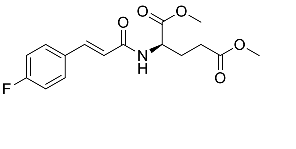	No	No	0.037	Non inhibitor	2.86
**28c**	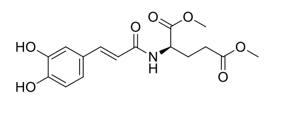	No	No	0.050	Non inhibitor	2.38
**29c**	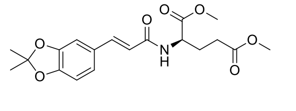	No	No	0.130	Non inhibitor	2.56
**30c**	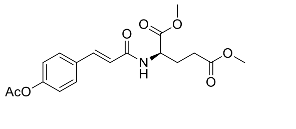	No	No	0.047	Inhibitor	2.50

Table 4 - *In silico* determination of % drug binding to plasma proteins and GI absorption of the synthesized compounds.

Compound	Structure	GI absorption	% drug binding to plasma proteins.
**11a**	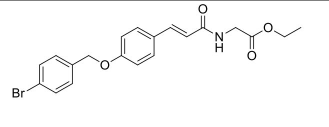	High	90.09
**12a**	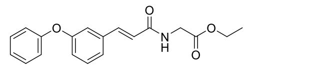	High	86.51
**13a**	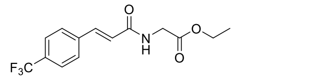	High	89.59
**14a**	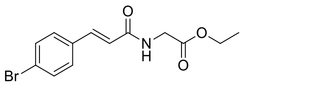	High	82.07
**15a**	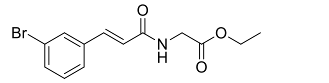	High	87.08
**16a**	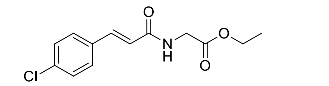	High	81.33
**17a**	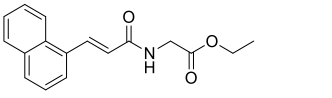	High	86.85
**18a**	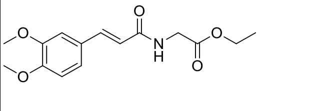	High	57.98

**19a**	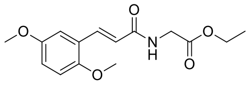	High	63.30
**20a**	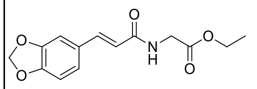	High	59.06
**21a**	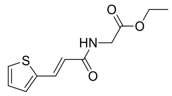	High	60.54
**22a**	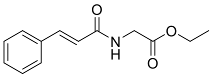	High	70.78
**23a**	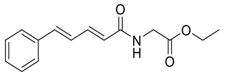	High	81.05
**24a**	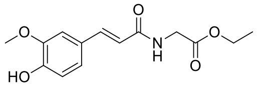	High	56.13
**25a**	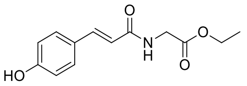	High	66.20
**26a**	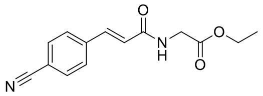	High	73.60
**27a**	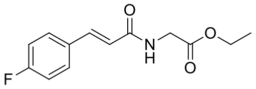	High	75.77
**28a**	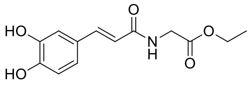	High	53.41
**29a**	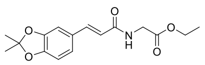	High	57.08
**30a**	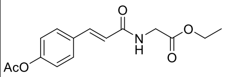	High	69.75
**11b**	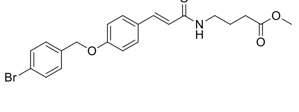	High	91.91

**12b**	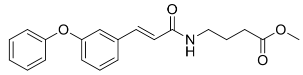	High	89.23
**13b**	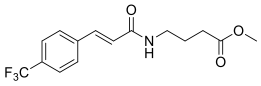	High	84.35
**14b**	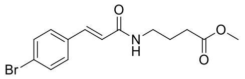	High	87.60
**15b**	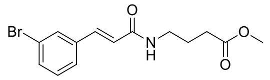	High	88.75
**16b**	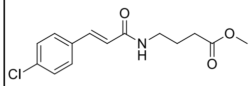	High	82.51
**17b**	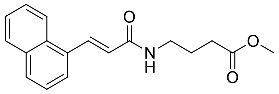	High	83.25
**18b**	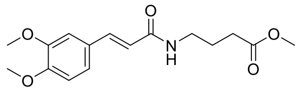	High	54.16
**19b**	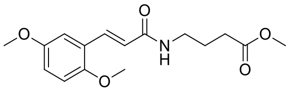	High	59.67
**20b**	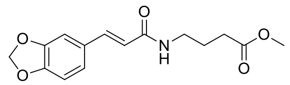	High	53.98
**21b**	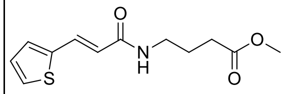	High	44.53
**22b**	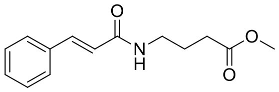	High	61.30
**23b**	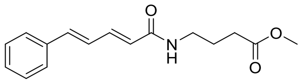	High	83.42
**24b**	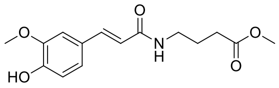	High	53.21
**25b**	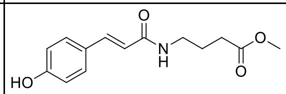	High	91.23

**26b**	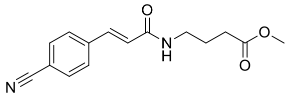	High	61.27
**27b**	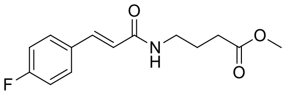	High	69.74
**28b**	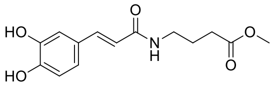	High	52.53
**29b**	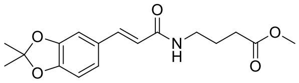	High	53.31
**30b**	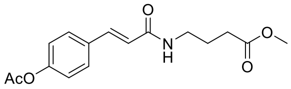	High	60.51
**11c**	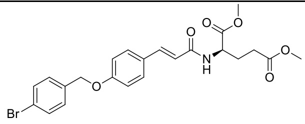	High	88.16
**12c**	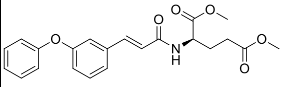	High	86.60
**13c**	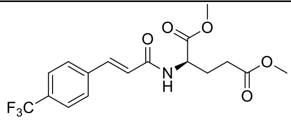	High	89.90
**14c**	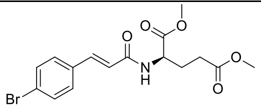	High	82.75
**15c**	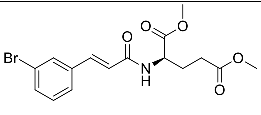	High	83.45
**16c**	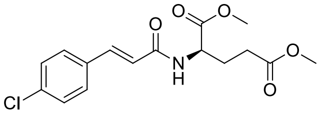	High	80.80
**17c**	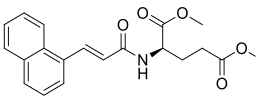	High	86.10
**18c**	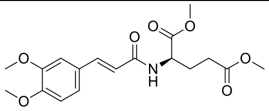	High	58.34

**19c**	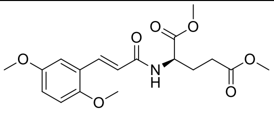	High	63.09
**20c**	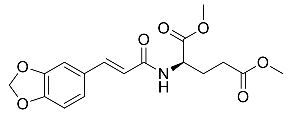	High	61.29
**21c**	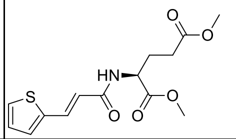	High	64.31
**22c**	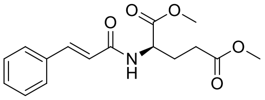	High	70.37
**23c**	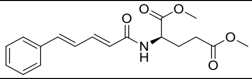	High	81.46
**24c**	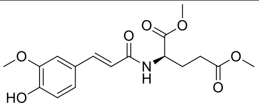	High	58.20
**25c**	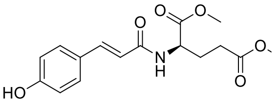	High	66.61
**26c**	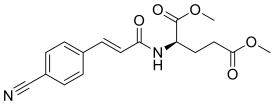	High	70.93
**27c**	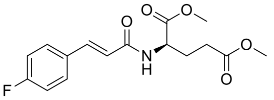	High	76.38
**28c**	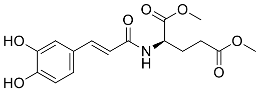	High	57.37
**29c**	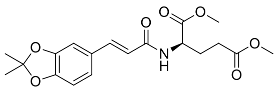	High	57.35
**30c**	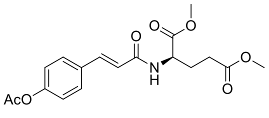	High	66.69

Table 5 - *In silico* generation of Phase I and Phase II metabolites for the studied molecules (in descending probability order).

Compound	Structure	Metabolites Generated		
		1st	2nd	3rd
**11a**	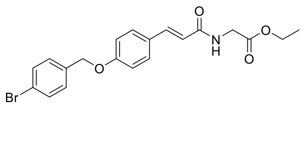	**0.79** 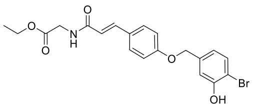	0.79 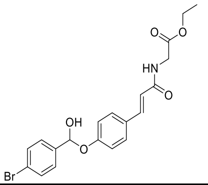	0.79 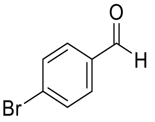
**12a**	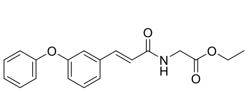	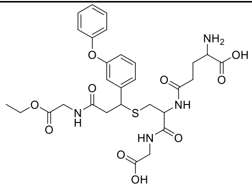 **0.68**	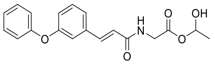 0.42	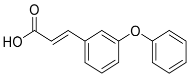 0.42
**13a**	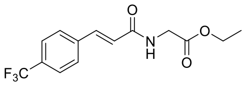	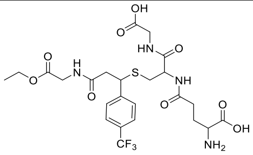 **0.80**	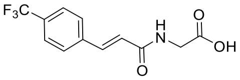 0.43	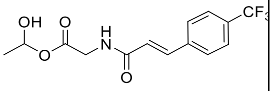 0.43
**14a**	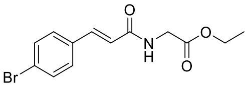	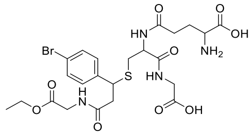 **0.79**	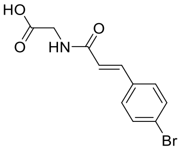 0.41	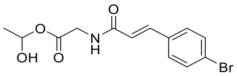 0.41
**15a**	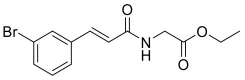	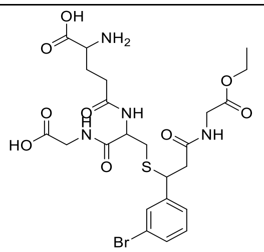 **0.79**	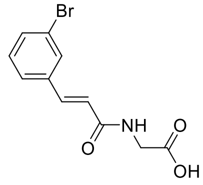 0.41	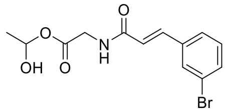 0.41
**16a**	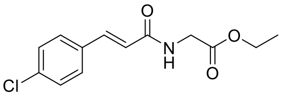	**0.79** 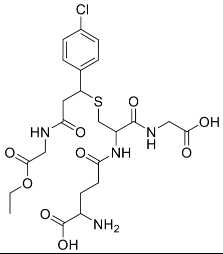	0.41 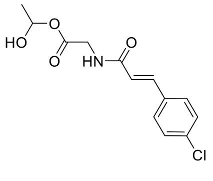	0.41 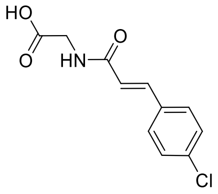
**17a**	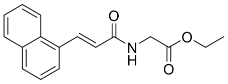	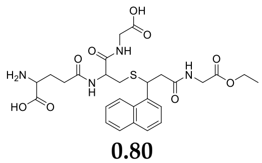 **0.80**	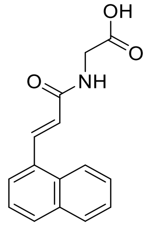	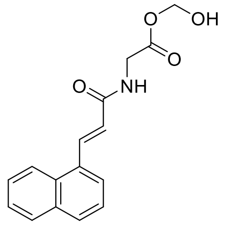

			0.43	0.43
**18a**	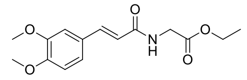	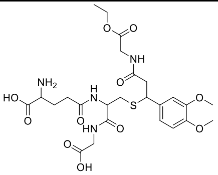 **0.75**	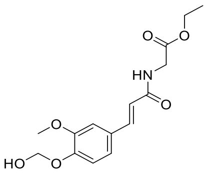 0.41	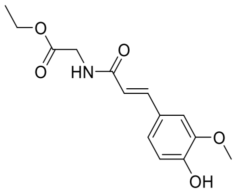 0.41
**19a**	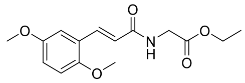	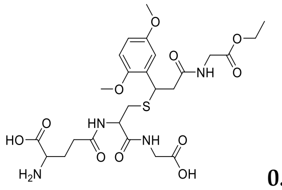 **0.80**	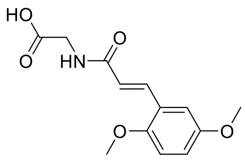 0.43	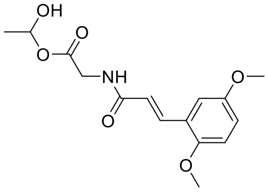 0.43
**20a**	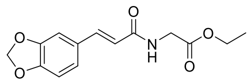	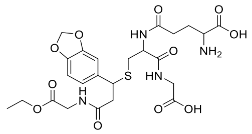 **0.80**	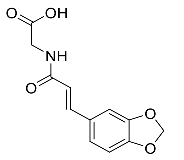 0.43	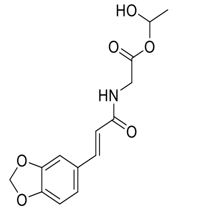 0.43
**21a**	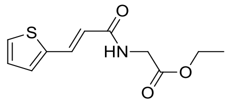	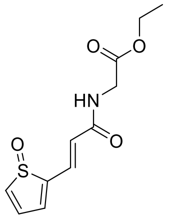 **0.45**	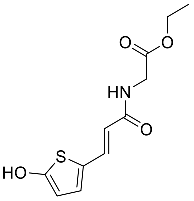 0.45	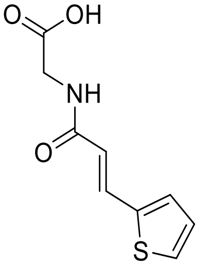 0.39
**22a**	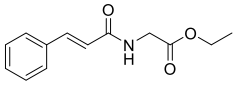	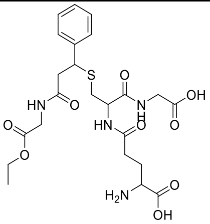 **0.79**	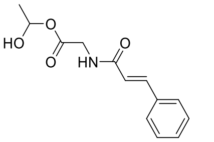 0.41	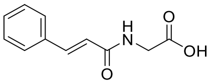 0.41
**23a**	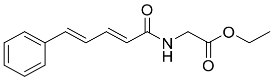	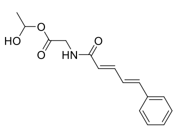 **0.40**	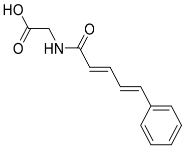 0.40	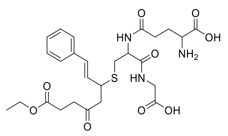 0.40
**24a**	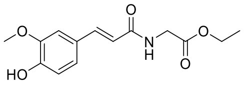	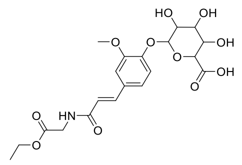 **0.96**	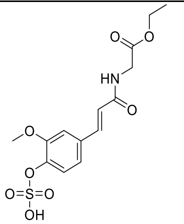 0.88	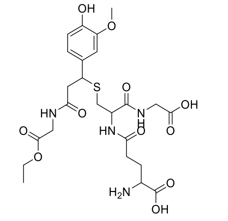 0.80

**25a**	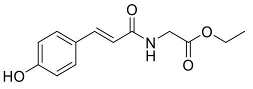	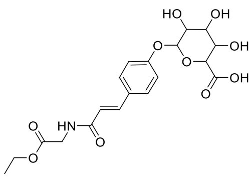 **0.98**	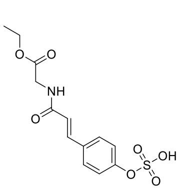 0.95	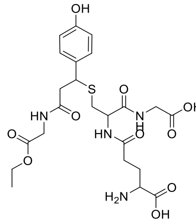 0.80
**26a**	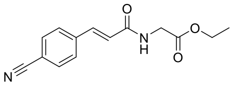	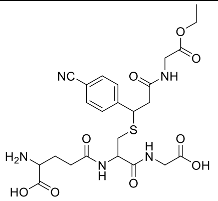 **0.80**	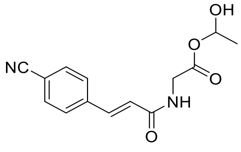 0.43	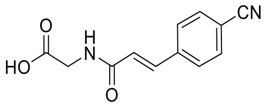 0.43
**27a**	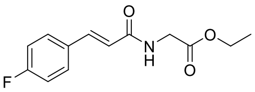	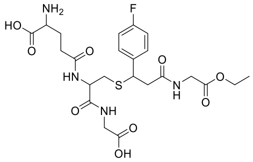 **0.79**	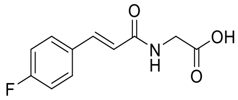 0.41	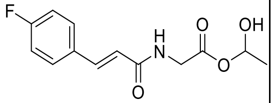 0.41
**28a**	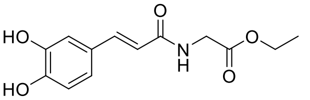	**0.97** 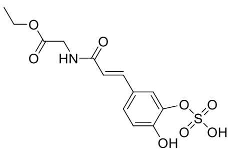	0.96 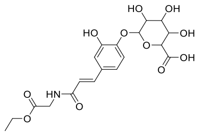	0.87 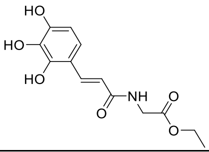
**29a**	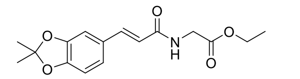	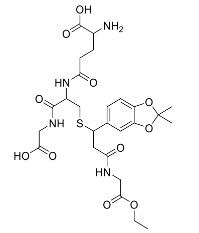 **0.75**	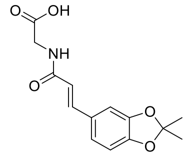 0.40	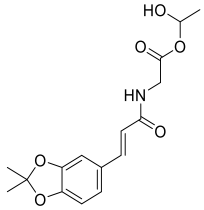 0.40
**30a**	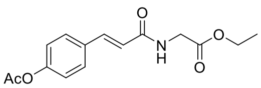	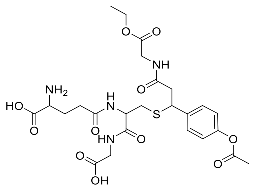 **0.72**	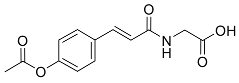 0.42	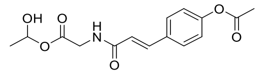 0.42
**11b**	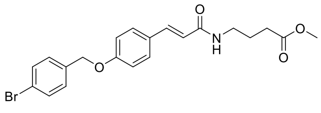	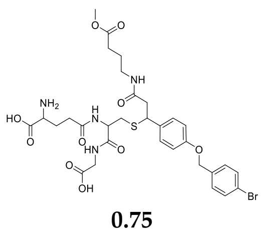 **0.75**	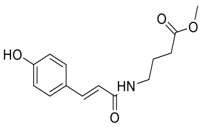 0.72	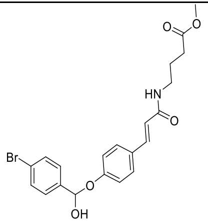 0.72

**12b**	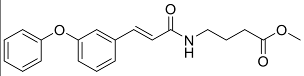	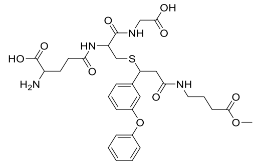 **0.80**	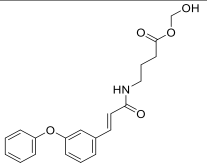 0.50	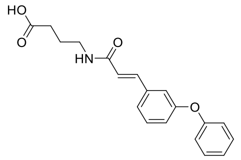 0.50
**13b**	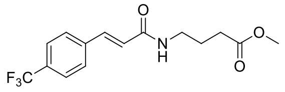	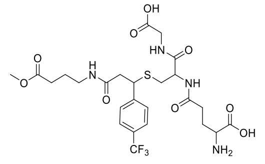 **0.97**	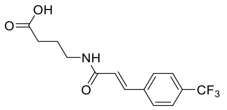 0.53	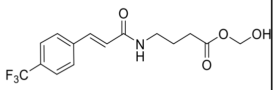 0.53
**14b**	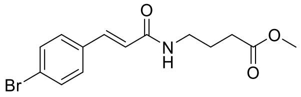	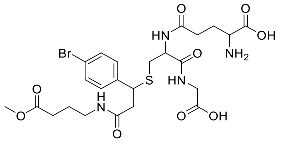 **0.96**	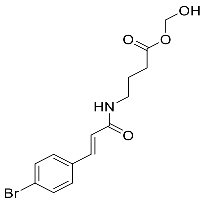 0.54	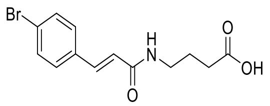 0.54
**15b**	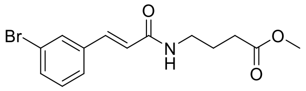	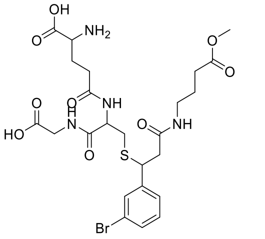 **0.96**	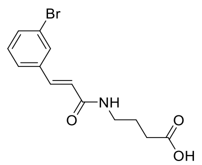 0.54	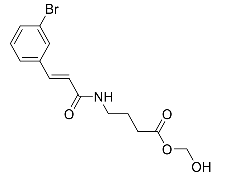 0.54
**16b**	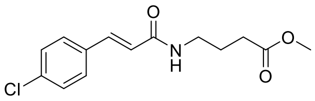	**0.96** 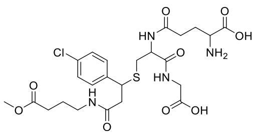	0.54 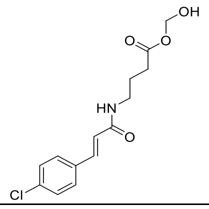	0.54 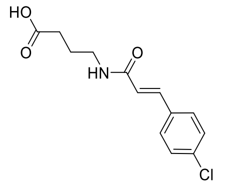
**17b**	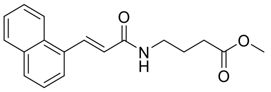	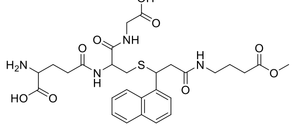 **0.96**	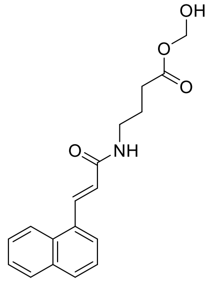 0.53	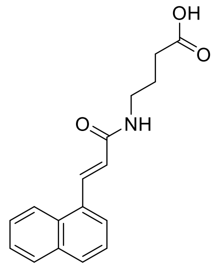 0.53
**18b**	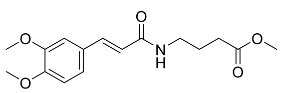	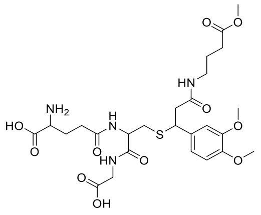 **0.93**	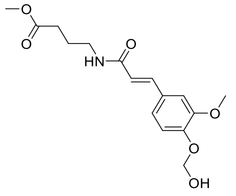 0.52	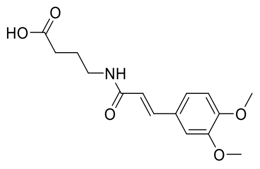 0.52

**19b**	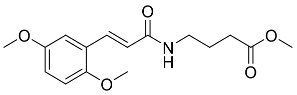	**0.97** 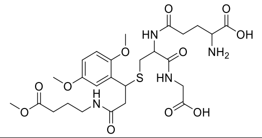	0.53 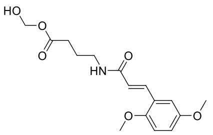	0.53 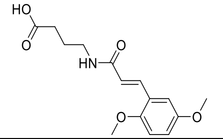
**20b**	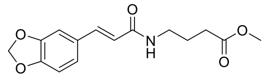	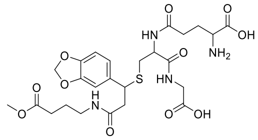 **0.97**	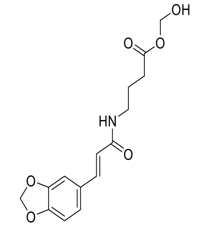 0.53	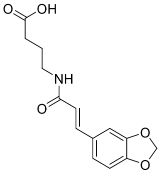 0.53
**21b**	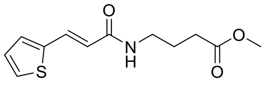	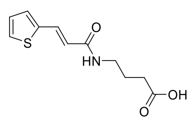 **0.56**	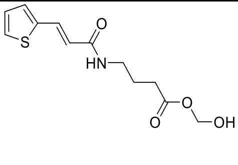 0.56	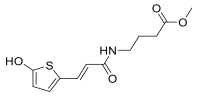 0.40
**22b**	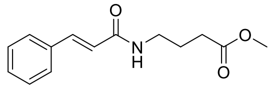	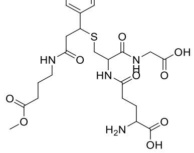 **0.96**	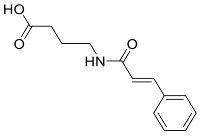 0.53	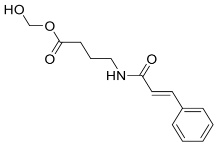 0.53
**23b**	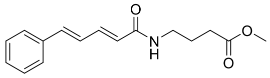	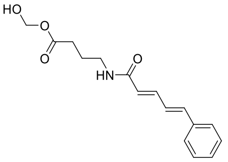 **0.52**	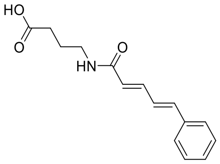 0.52	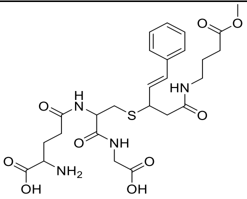 0.46
**24b**	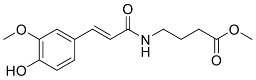	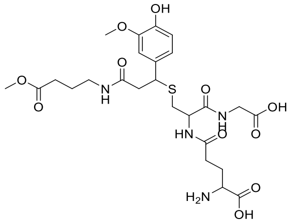 **0.97**	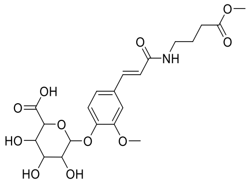 0.96	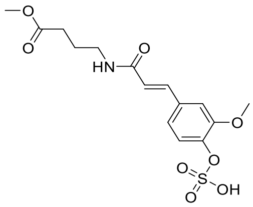 0.90
**25b**	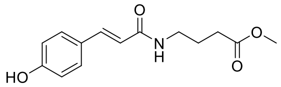	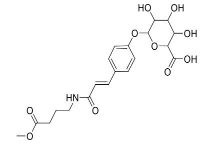 **0.98**	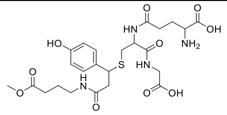 0.96	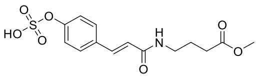 0.96
**26b**	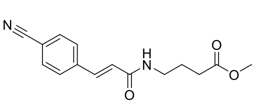	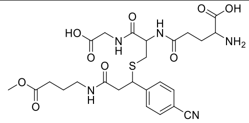 **0.96**	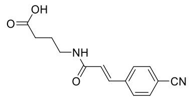 0.53	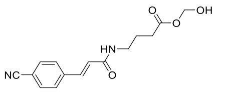 0.53

**27b**	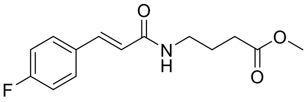	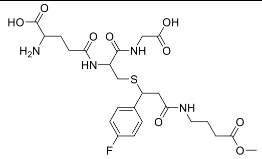 **0.96**	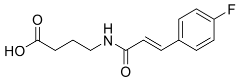 0.53	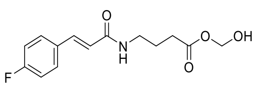 0.53
**28b**	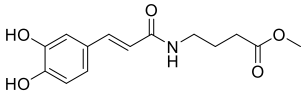	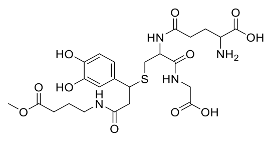 **0.96**	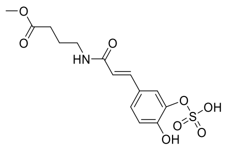 0.96	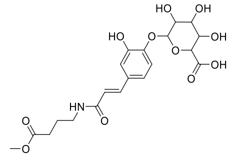 0.96
**29b**	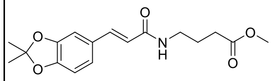	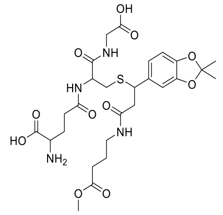 **0.93**	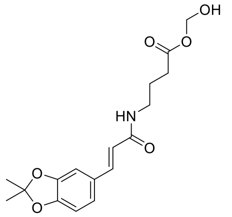 0.52	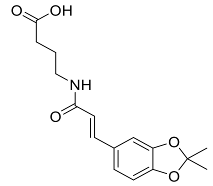 0.52
**30b**	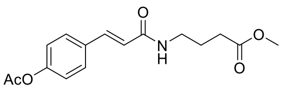	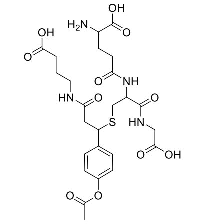 **0.85**	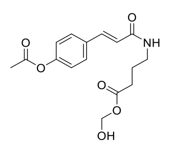 0.52	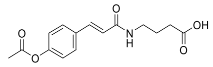 0.52
**11c**	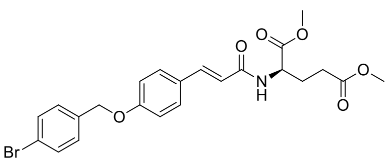	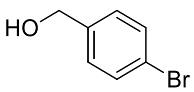 **0.73**	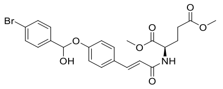 0.73	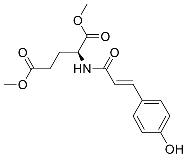 0.73
**12c**	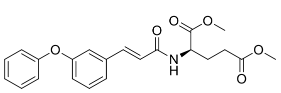	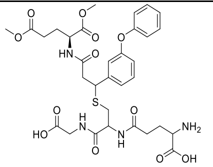 **0.62**	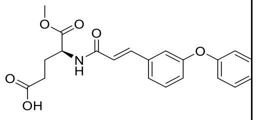 0.52	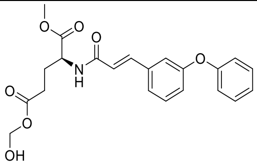 0.52
**13c**	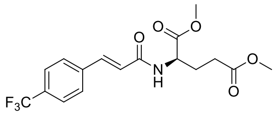	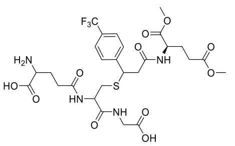 **0.77**	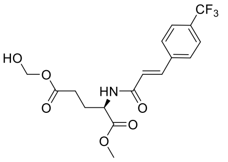 0.54	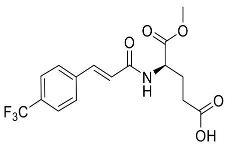 0.54
**14c**	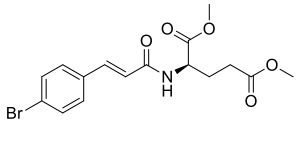	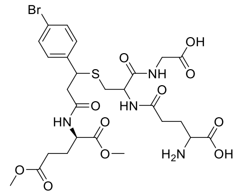 **0.73**	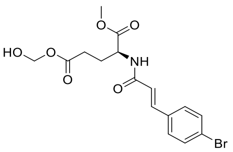 0.56	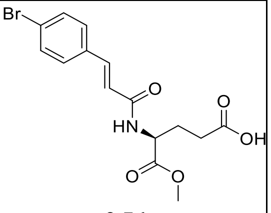 0.56

**15c**	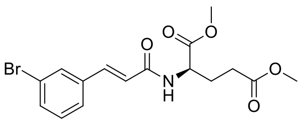	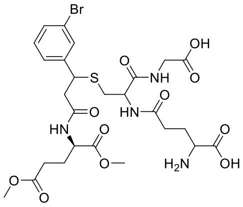 **0.75**	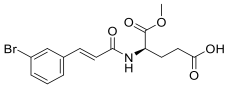 0.56	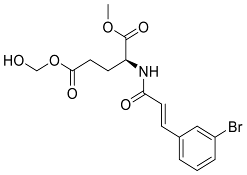 0.56
**16c**	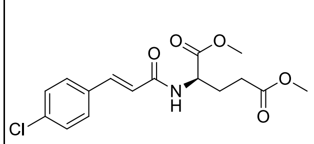	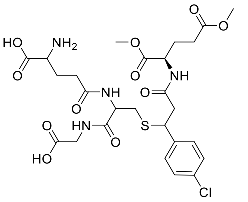 **0.76**	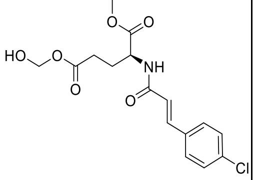 0.56	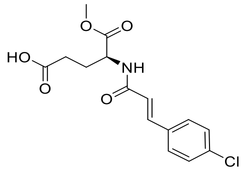 0.56
**17c**	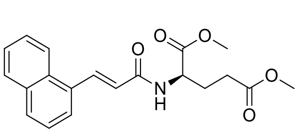	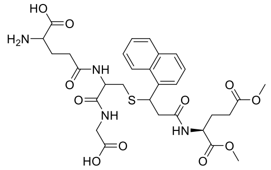 **0.76**	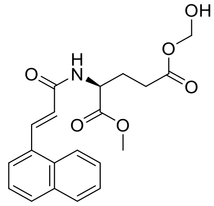 0.54	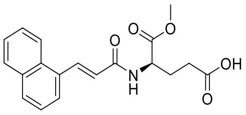 0.54
**18c**	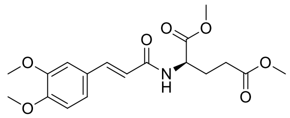	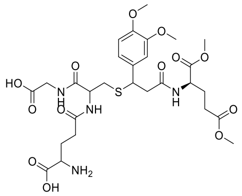 **0.73**	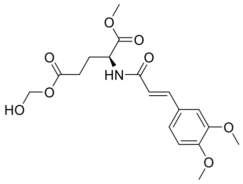 0.53	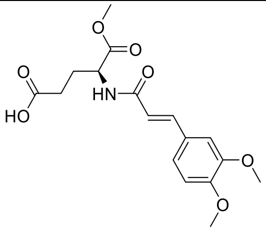 0.53
**19c**	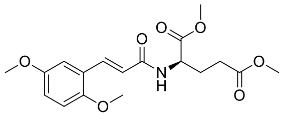	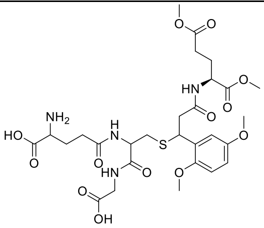 **0.76**	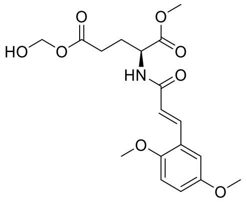 0.54	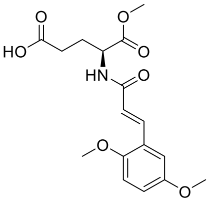 0.54
**20c**	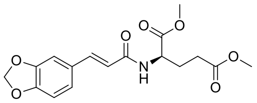	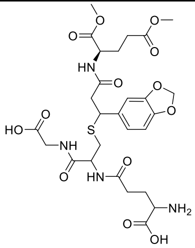 **0.77**	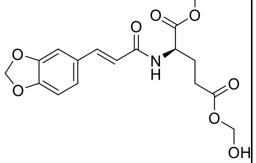 0.54	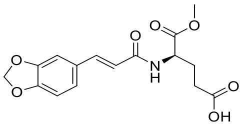 0.54
**21c**	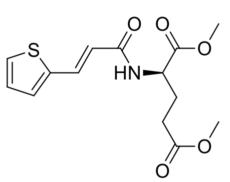	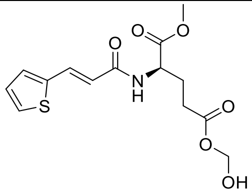 **0.58**	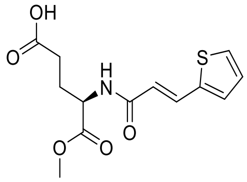 0.58	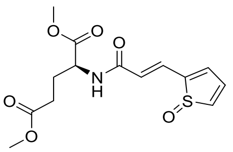 0.39

**22c**	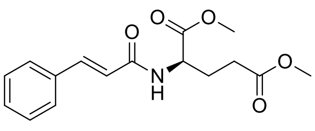	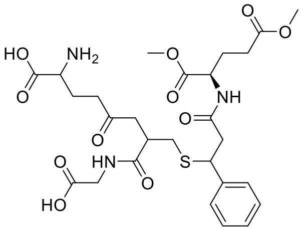 **0.76**	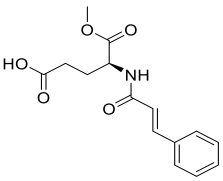 0.56	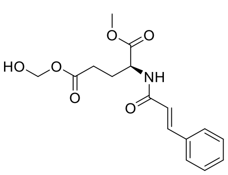 0.56
**23c**	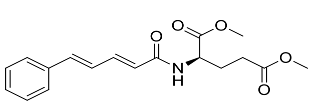	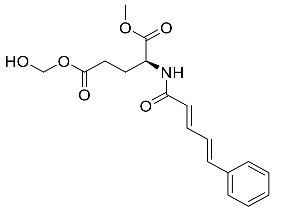 **0.53**	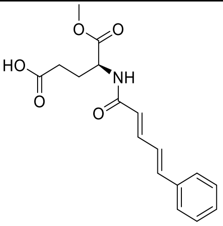 0.53	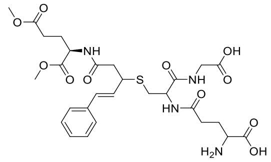 0.40
**24c**	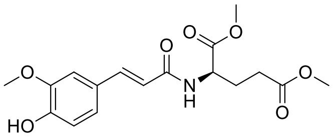	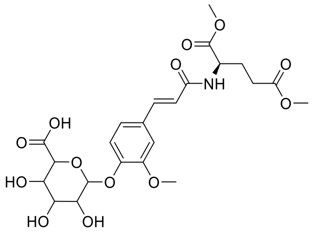 **0.96**	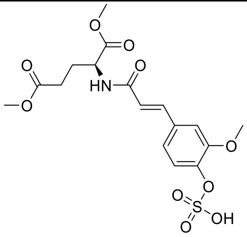 0.90	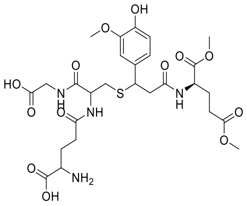 0.77
**25c**	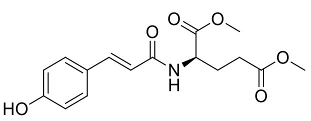	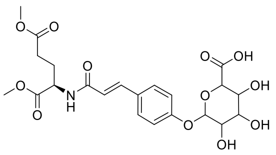 **0.98**	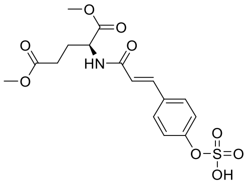 0.96	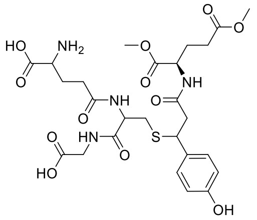 0.76
**26c**	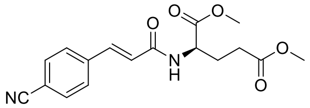	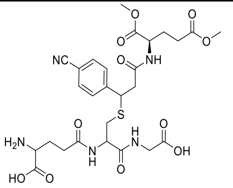 **0.76**	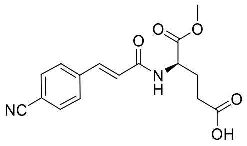 0.54	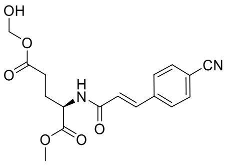 0.54
**27c**	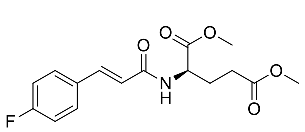	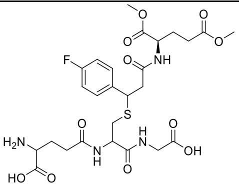 **0.76**	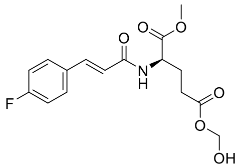 0.56	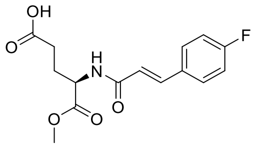 0.56
**28c**	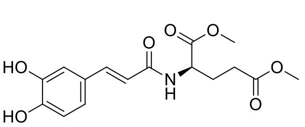	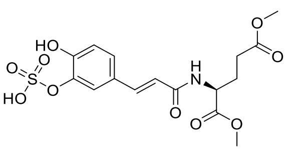 **0.96**	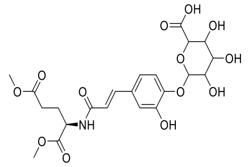 0.96	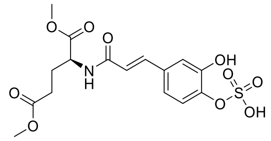 0.87

**29c**	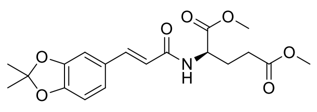	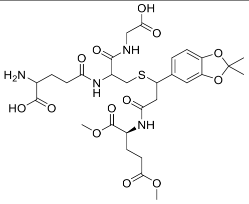 **0.73**	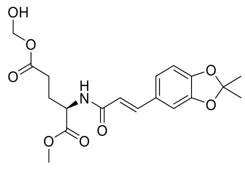 0.53	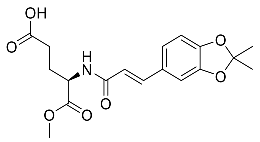 0.53
**30c**	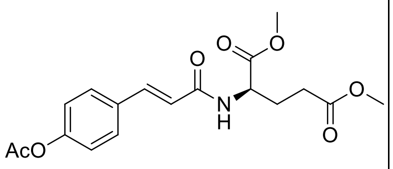	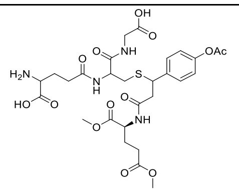 **0.66**	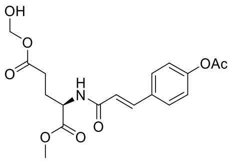 0.53	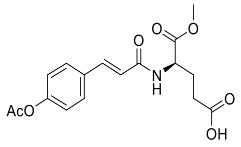 0.53

Table 6 - ***In silico*** **determination of the compounds' P450 inhibitory probability.**

Compound	CYP1A2	CYP2C19	CYP2C9	CYP2D6	CYP3A4
**11a**	Non-Inhibitor	Non-Inhibitor	Non-Inhibitor	Non-Inhibitor	Non-Inhibitor
**12a**	Non-Inhibitor	Non-Inhibitor	Non-Inhibitor	Non-Inhibitor	Non-Inhibitor
**13a**	Non-Inhibitor	Non-Inhibitor	Non-Inhibitor	Non-Inhibitor	Non-Inhibitor
**14a**	Non-Inhibitor	Non-Inhibitor	Non-Inhibitor	Non-Inhibitor	Non-Inhibitor
**15a**	Non-Inhibitor	Non-Inhibitor	Non-Inhibitor	Non-Inhibitor	Non-Inhibitor
**16a**	Non-Inhibitor	Non-Inhibitor	Non-Inhibitor	Non-Inhibitor	Non-Inhibitor
**17a**	Non-Inhibitor	Non-Inhibitor	Non-Inhibitor	Non-Inhibitor	Non-Inhibitor
**18a**	Non-Inhibitor	Non-Inhibitor	Non-Inhibitor	Non-Inhibitor	Non-Inhibitor
**19a**	Non-Inhibitor	Non-Inhibitor	Non-Inhibitor	Non-Inhibitor	Non-Inhibitor
**20a**	Non-Inhibitor	Non-Inhibitor	Non-Inhibitor	Non-Inhibitor	Non-Inhibitor
**21a**	Non-Inhibitor	Non-Inhibitor	Non-Inhibitor	Non-Inhibitor	Non-Inhibitor
**22a**	Non-Inhibitor	Non-Inhibitor	Non-Inhibitor	Non-Inhibitor	Non-Inhibitor
**23a**	Non-Inhibitor	Non-Inhibitor	Non-Inhibitor	Non-Inhibitor	Non-Inhibitor
**24a**	Non-Inhibitor	Non-Inhibitor	Non-Inhibitor	Non-Inhibitor	Non-Inhibitor
**25a**	Non-Inhibitor	Non-Inhibitor	Non-Inhibitor	Non-Inhibitor	Non-Inhibitor
**26a**	Non-Inhibitor	Non-Inhibitor	Non-Inhibitor	Non-Inhibitor	Non-Inhibitor
**27a**	Non-Inhibitor	Non-Inhibitor	Non-Inhibitor	Non-Inhibitor	Non-Inhibitor
**28a**	Non-Inhibitor	Non-Inhibitor	Non-Inhibitor	Non-Inhibitor	Non-Inhibitor
**29a**	Non-Inhibitor	Non-Inhibitor	Non-Inhibitor	Non-Inhibitor	Non-Inhibitor
**30a**	Non-Inhibitor	Non-Inhibitor	Non-Inhibitor	Non-Inhibitor	Non-Inhibitor
**11b**	Non-Inhibitor	Non-Inhibitor	Non-Inhibitor	Non-Inhibitor	Non-Inhibitor
**12b**	Non-Inhibitor	Non-Inhibitor	Non-Inhibitor	Non-Inhibitor	Non-Inhibitor
**13b**	Non-Inhibitor	Non-Inhibitor	Non-Inhibitor	Non-Inhibitor	Non-Inhibitor
**14b**	Non-Inhibitor	Non-Inhibitor	Non-Inhibitor	Non-Inhibitor	Non-Inhibitor
**15b**	Non-Inhibitor	Non-Inhibitor	Non-Inhibitor	Non-Inhibitor	Non-Inhibitor
**16b**	Non-Inhibitor	Non-Inhibitor	Non-Inhibitor	Non-Inhibitor	Non-Inhibitor
**17b**	Non-Inhibitor	Non-Inhibitor	Non-Inhibitor	Non-Inhibitor	Non-Inhibitor
**18b**	Non-Inhibitor	Non-Inhibitor	Non-Inhibitor	Non-Inhibitor	Non-Inhibitor
**19b**	Non-Inhibitor	Non-Inhibitor	Non-Inhibitor	Non-Inhibitor	Non-Inhibitor
**20b**	Non-Inhibitor	Non-Inhibitor	Non-Inhibitor	Non-Inhibitor	Non-Inhibitor
**21b**	Non-Inhibitor	Non-Inhibitor	Non-Inhibitor	Non-Inhibitor	Non-Inhibitor
**22b**	Non-Inhibitor	Non-Inhibitor	Non-Inhibitor	Non-Inhibitor	Non-Inhibitor
**23b**	Non-Inhibitor	Non-Inhibitor	Non-Inhibitor	Non-Inhibitor	Non-Inhibitor
**24b**	Non-Inhibitor	Non-Inhibitor	Non-Inhibitor	Non-Inhibitor	Non-Inhibitor
**25b**	Non-Inhibitor	Non-Inhibitor	Non-Inhibitor	Non-Inhibitor	Non-Inhibitor
**26b**	Non-Inhibitor	Non-Inhibitor	Non-Inhibitor	Non-Inhibitor	Non-Inhibitor
**27b**	Non-Inhibitor	Non-Inhibitor	Non-Inhibitor	Non-Inhibitor	Non-Inhibitor

**28b**	Non-Inhibitor	Non-Inhibitor	Non-Inhibitor	Non-Inhibitor	Non-Inhibitor
**29b**	Non-Inhibitor	Non-Inhibitor	Non-Inhibitor	Non-Inhibitor	Non-Inhibitor
**30b**	Non-Inhibitor	Non-Inhibitor	Non-Inhibitor	Non-Inhibitor	Non-Inhibitor
**11c**	Non-Inhibitor	Non-Inhibitor	Non-Inhibitor	Non-Inhibitor	Non-Inhibitor
**12c**	Non-Inhibitor	Non-Inhibitor	Non-Inhibitor	Non-Inhibitor	Non-Inhibitor
**13c**	Non-Inhibitor	Non-Inhibitor	Non-Inhibitor	Non-Inhibitor	Non-Inhibitor
**14c**	Non-Inhibitor	Non-Inhibitor	Non-Inhibitor	Non-Inhibitor	Non-Inhibitor
**15c**	Non-Inhibitor	Non-Inhibitor	Non-Inhibitor	Non-Inhibitor	Non-Inhibitor
**16c**	Non-Inhibitor	Non-Inhibitor	Non-Inhibitor	Non-Inhibitor	Non-Inhibitor
**17c**	Non-Inhibitor	Non-Inhibitor	Non-Inhibitor	Non-Inhibitor	Non-Inhibitor
**18c**	Non-Inhibitor	Non-Inhibitor	Non-Inhibitor	Non-Inhibitor	Non-Inhibitor
**19c**	Non-Inhibitor	Non-Inhibitor	Non-Inhibitor	Non-Inhibitor	Non-Inhibitor
**20c**	Non-Inhibitor	Non-Inhibitor	Non-Inhibitor	Non-Inhibitor	Non-Inhibitor
**21c**	Non-Inhibitor	Non-Inhibitor	Non-Inhibitor	Non-Inhibitor	Non-Inhibitor
**22c**	Non-Inhibitor	Non-Inhibitor	Non-Inhibitor	Non-Inhibitor	Non-Inhibitor
**23c**	Non-Inhibitor	Non-Inhibitor	Non-Inhibitor	Non-Inhibitor	Non-Inhibitor
**24c**	Non-Inhibitor	Non-Inhibitor	Non-Inhibitor	Non-Inhibitor	Non-Inhibitor
**25c**	Non-Inhibitor	Non-Inhibitor	Non-Inhibitor	Non-Inhibitor	Non-Inhibitor
**26c**	Non-Inhibitor	Non-Inhibitor	Non-Inhibitor	Non-Inhibitor	Non-Inhibitor
**27c**	Non-Inhibitor	Non-Inhibitor	Non-Inhibitor	Non-Inhibitor	Non-Inhibitor
**28c**	Non-Inhibitor	Non-Inhibitor	Non-Inhibitor	Non-Inhibitor	Non-Inhibitor
**29c**	Non-Inhibitor	Non-Inhibitor	Non-Inhibitor	Non-Inhibitor	Non-Inhibitor
**30c**	Non-Inhibitor	Non-Inhibitor	Non-Inhibitor	Non-Inhibitor	Non-Inhibitor

Table 7 - ***In silico*** **evaluation of compounds' probability to interact with several targets related to liver toxicity.**

Compound	Structure	BSEP inhib.	BSEP trans.	P_{gp} inhib.	P_{gp} trans.	MRP4 inhib.	MRP2 trans.	MRP3 inhib.	MRP3 trans.
**11a**	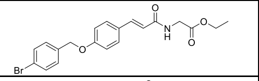	Negative	Negative	Negative	Negative	Positive	Positive	Positive	Negative
**11b**	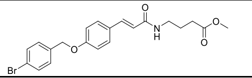	Negative	Negative	Negative	Negative	Positive	Positive	Positive	Negative
**11c**	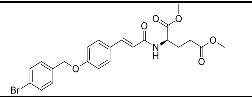	Positive	Negative	Negative	Negative	Positive	Positive	Positive	Negative
**12a**	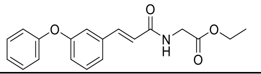	Negative	Negative	Negative	Negative	Positive	Positive	Negative	Negative
**12b**	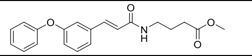	Negative	Negative	Negative	Negative	Positive	Positive	Negative	Negative
**12c**	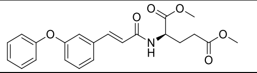	Positive	Negative	Negative	Negative	Positive	Positive	Positive	Negative
**13a**	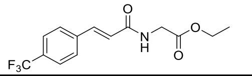	Negative	Positive	Negative	Negative	Positive	Positive	Negative	Negative

**13b**	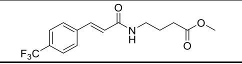	Negative	Positive	Negative	Negative	Positive	Negative	Negative	Positive
**13c**	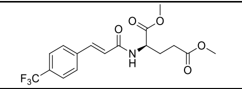	Negative	Positive	Negative	Negative	Positive	Negative	Negative	Positive
**14a**	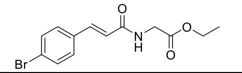	Negative	Positive	Negative	Negative	Positive	Negative	Negative	Positive
**14b**	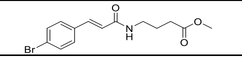	Negative	Negative	Negative	Negative	Positive	Negative	Negative	Positive
**14c**	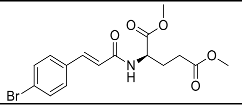	Negative	Positive	Negative	Negative	Positive	Positive	Negative	Positive
**15a**	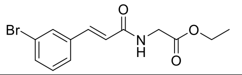	Negative	Positive	Negative	Negative	Positive	Negative	Negative	Positive
**15b**	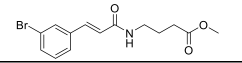	Negative	Negative	Negative	Negative	Positive	Negative	Negative	Positive
**15c**	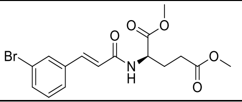	Negative	Positive	Negative	Negative	Positive	Positive	Negative	Positive
**16a**	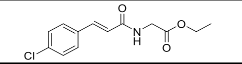	Negative	Negative	Negative	Negative	Positive	Positive	Negative	Positive
**16b**	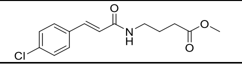	Negative	Negative	Negative	Negative	Positive	Positive	Negative	Positive
**16c**	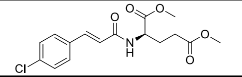	Negative	Negative	Negative	Negative	Positive	Positive	Negative	Negative
**17a**	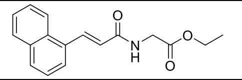	Negative	Negative	Negative	Negative	Positive	Negative	Negative	Negative
**17b**	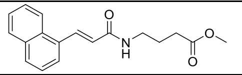	Negative	Negative	Negative	Negative	Positive	Positive	Negative	Negative
**17c**	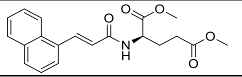	Negative	Positive	Negative	Negative	Positive	Positive	Negative	Negative
**18a**	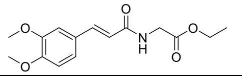	Negative	Negative	Negative	Negative	Positive	Positive	Negative	Negative
**18b**	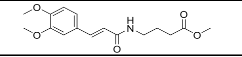	Negative	Negative	Negative	Negative	Positive	Negative	Negative	Positive
**18c**	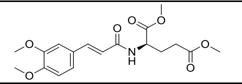	Negative	Negative	Negative	Negative	Positive	Positive	Negative	Negative
**19a**	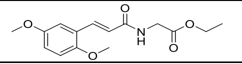	Negative	Negative	Negative	Negative	Positive	Positive	Negative	Positive
**19b**	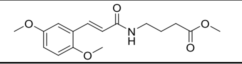	Negative	Negative	Negative	Negative	Positive	Negative	Negative	Positive
**19c**	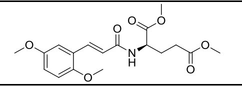	Negative	Negative	Negative	Negative	Positive	Positive	Negative	Negative
**20a**	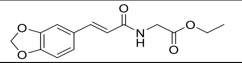	Negative	Negative	Negative	Negative	Positive	Positive	Negative	Negative
**20b**	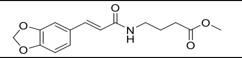	Negative	Negative	Negative	Negative	Positive	Positive	Negative	Negative
**20c**	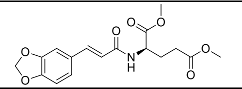	Negative	Negative	Negative	Negative	Positive	Positive	Negative	Negative
**21a**	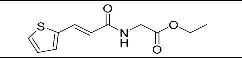	Negative	Negative	Negative	Negative	Negative	Negative	Negative	Positive
**21b**	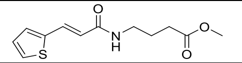	Negative	Negative	Negative	Negative	Negative	Negative	Negative	Negative

**21c**	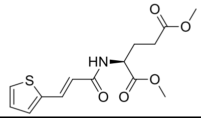	Negative	Positive	Negative	Negative	Positive	Negative	Negative	Positive
**22a**	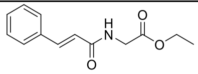	Negative	Positive	Negative	Negative	Negative	Positive	Negative	Positive
**22b**	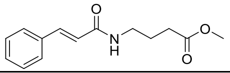	Negative	Positive	Negative	Negative	Negative	Positive	Negative	Positive
**22c**	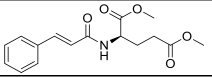	Negative	Positive	Negative	Negative	Positive	Positive	Negative	Negative
**23a**	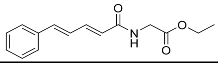	Negative	Positive	Negative	Negative	Negative	Positive	Negative	Positive
**23b**	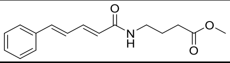	Negative	Positive	Negative	Negative	Negative	Positive	Negative	Positive
**23c**	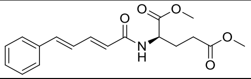	Negative	Positive	Negative	Negative	Positive	Positive	Negative	Positive
**24a**	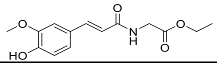	Negative	Positive	Negative	Negative	Positive	Negative	Negative	Positive
**24b**	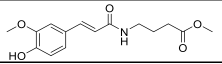	Negative	Positive	Negative	Negative	Positive	Negative	Negative	Positive
**24c**	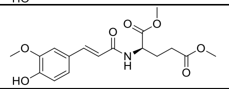	Negative	Positive	Negative	Negative	Positive	Positive	Negative	Negative
**25a**	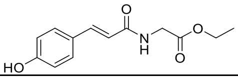	Negative	Positive	Negative	Negative	Positive	Negative	Negative	Positive
**25b**	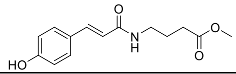	Negative	Negative	Negative	Negative	Negative	Negative	Negative	Positive
**25c**	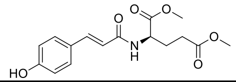	Negative	Positive	Negative	Negative	Positive	Negative	Negative	Negative
**26a**	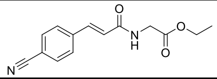	Negative	Negative	Negative	Negative	Positive	Positive	Negative	Positive
**26b**	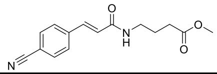	Negative	Negative	Negative	Negative	Positive	Positive	Negative	Positive
**26c**	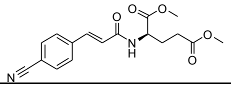	Negative	Negative	Negative	Negative	Positive	Positive	Negative	Negative
**27a**	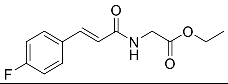	Negative	Positive	Negative	Negative	Positive	Negative	Negative	Positive
**27b**	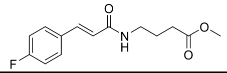	Negative	Positive	Negative	Negative	Positive	Positive	Negative	Positive
**27c**	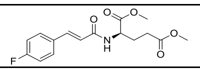	Negative	Positive	Negative	Negative	Positive	Positive	Negative	Positive
**28a**	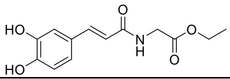	Negative	Positive	Negative	Negative	Negative	Negative	Negative	Negative
**28b**	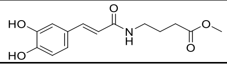	Negative	Positive	Negative	Negative	Positive	Negative	Negative	Positive
**28c**	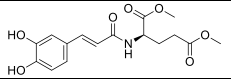	Negative	Positive	Negative	Negative	Positive	Negative	Negative	Positive
**29a**	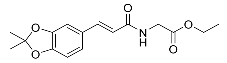	Negative	Negative	Negative	Negative	Positive	Positive	Not available	Not available
**29b**	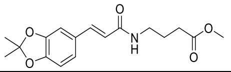	Negative	Negative	Negative	Negative	Positive	Positive	Not available	Not available

**29c**	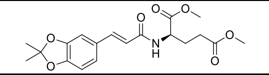	Negative	Negative	Negative	Negative	Positive	Positive	Not available	Not available
**30a**	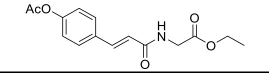	Negative	Negative	Negative	Negative	Positive	Positive	Not available	Not available
**30b**	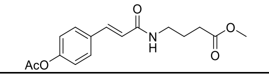	Negative	Negative	Negative	Negative	Positive	Positive	Not available	Not available
**30c**	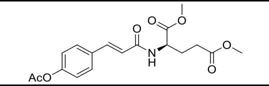	Positive	Negative	Negative	Negative	Positive	Positive	Not available	Not available

Table 8 - *In silico* study of the designed compounds' probability to interact with several hepatotoxicity-related targets.

Compound	Structure	BCRP inhib.	BCRP trans.	OATP1B1 inhib.	OATP1B3 inhib.	Drug-Induced liver injury	Hyperbilirubinemia	Cholestasis
**11a**	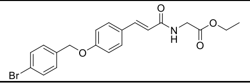	Negative	Positive	Negative	Negative	Positive	Negative	Positive
**11b**	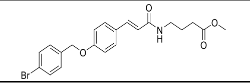	Negative	Positive	Negative	Negative	Positive	Positive	Positive
**11c**	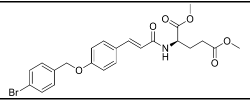	Negative	Positive	Positive	Positive	Positive	Negative	Positive
**12a**	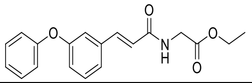	Negative	Positive	Negative	Negative	Positive	Negative	Negative
**12b**	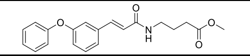	Negative	Positive	Negative	Negative	Positive	Negative	Negative
**12c**	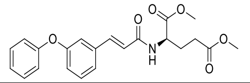	Negative	Positive	Negative	Negative	Positive	Negative	Positive
**13a**	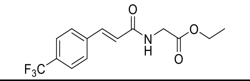	Negative	Positive	Negative	Negative	Positive	Negative	Positive
**13b**	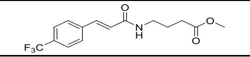	Negative	Negative	Negative	Negative	Positive	Negative	Positive
**13c**	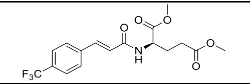	Negative	Negative	Negative	Negative	Positive	Negative	Positive
**14a**	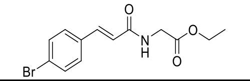	Negative	Positive	Negative	Negative	Positive	Negative	Positive
**14b**	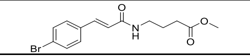	Negative	Positive	Negative	Negative	Positive	Negative	Positive
**14c**	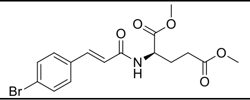	Negative	Negative	Negative	Negative	Positive	Negative	Positive
**15a**	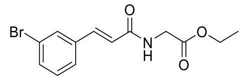	Negative	Positive	Negative	Negative	Positive	Negative	Positive
**15b**	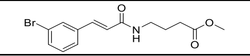	Negative	Positive	Negative	Negative	Positive	Negative	Positive
**15c**	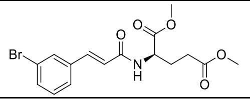	Negative	Negative	Negative	Negative	Positive	Negative	Positive
**16a**	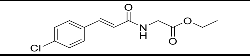	Negative	Positive	Negative	Negative	Positive	Negative	Positive

**16b**	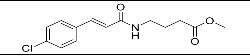	Negative	Positive	Negative	Negative	Positive	Negative	Positive
**16c**	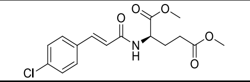	Negative	Positive	Negative	Negative	Positive	Negative	Positive
**17a**	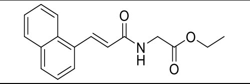	Negative	Positive	Negative	Negative	Positive	Negative	Positive
**17b**	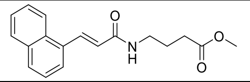	Negative	Negative	Negative	Negative	Positive	Positive	Positive
**17c**	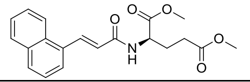	Negative	Positive	Negative	Negative	Positive	Negative	Positive
**18a**	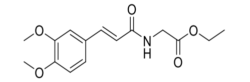	Negative	Positive	Negative	Negative	Positive	Negative	Positive
**18b**	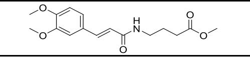	Positive	Positive	Negative	Negative	Positive	Negative	Positive
**18c**	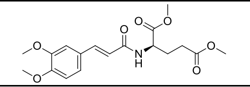	Negative	Positive	Negative	Negative	Positive	Negative	Positive
**19a**	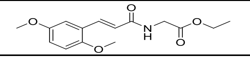	Negative	Positive	Negative	Negative	Positive	Negative	Positive
**19b**	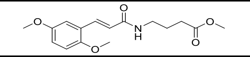	Negative	Positive	Negative	Negative	Positive	Positive	Positive
**19c**	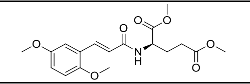	Negative	Positive	Negative	Negative	Positive	Negative	Positive
**20a**	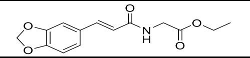	Negative	Positive	Negative	Negative	Positive	Negative	Positive
**20b**	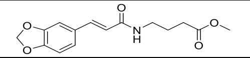	Negative	Positive	Negative	Negative	Positive	Positive	Positive
**20c**	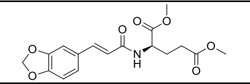	Negative	Positive	Negative	Negative	Positive	Negative	Positive
**21a**	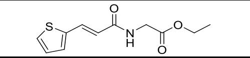	Negative	Positive	Negative	Negative	Positive	Negative	Positive
**21b**	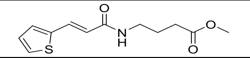	Negative	Negative	Negative	Negative	Positive	Negative	Positive
**21c**	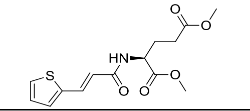	Negative	Negative	Negative	Negative	Positive	Negative	Positive
**22a**	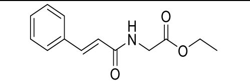	Negative	Positive	Negative	Negative	Positive	Negative	Positive
**22b**	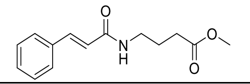	Negative	Positive	Negative	Negative	Positive	Negative	Positive
**22c**	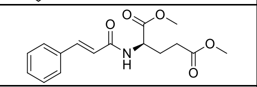	Negative	Positive	Negative	Negative	Positive	Negative	Positive
**23a**	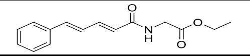	Negative	Positive	Negative	Negative	Positive	Negative	Positive
**23b**	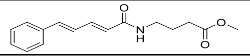	Negative	Positive	Negative	Negative	Positive	Negative	Positive
**23c**	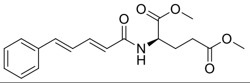	Negative	Positive	Negative	Negative	Positive	Negative	Positive
**24a**	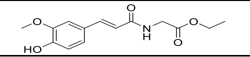	Negative	Positive	Negative	Negative	Negative	Negative	Negative
**24b**	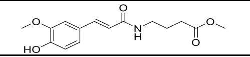	Negative	Positive	Negative	Negative	Positive	Negative	Positive

**24c**	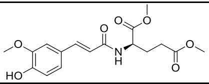	Negative	Positive	Negative	Negative	Positive	Negative	Positive
**25a**	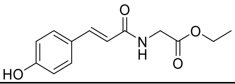	Negative	Positive	Negative	Negative	Positive	Negative	Positive
**25b**	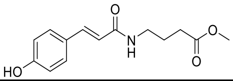	Negative	Positive	Negative	Negative	Positive	Negative	Positive
**25c**	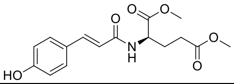	Negative	Positive	Negative	Negative	Positive	Negative	Positive
**26a**	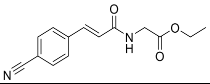	Negative	Positive	Negative	Negative	Positive	Negative	Positive
**26b**	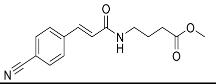	Negative	Positive	Negative	Negative	Positive	Negative	Positive
**26c**	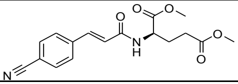	Negative	Positive	Negative	Negative	Positive	Negative	Positive
**27a**	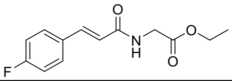	Negative	Positive	Negative	Negative	Positive	Negative	Negative
**27b**	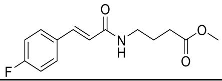	Negative	Positive	Negative	Negative	Positive	Negative	Positive
**27c**	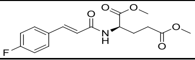	Negative	Positive	Negative	Negative	Positive	Negative	Positive
**28a**	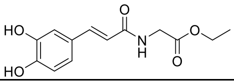	Negative	Positive	Negative	Negative	Negative	Negative	Positive
**28b**	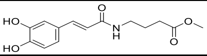	Negative	Positive	Negative	Negative	Positive	Negative	Positive
**28c**	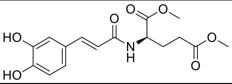	Negative	Positive	Negative	Negative	Positive	Negative	Positive
**29a**	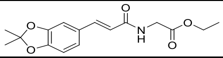	Negative	Positive	Negative	Negative	Positive	Negative	Negative
**29b**	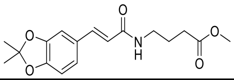	Negative	Positive	Negative	Negative	Positive	Negative	Positive
**29c**	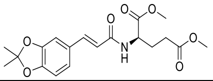	Negative	Positive	Negative	Negative	Positive	Negative	Positive
**30a**	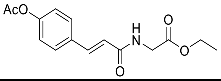	Negative	Positive	Negative	Negative	Positive	Negative	Negative
**30b**	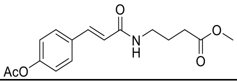	Negative	Positive	Negative	Negative	Positive	Negative	Positive
**30c**	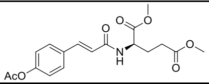	Negative	Positive	Negative	Negative	Positive	Negative	Positive

2. Docking poses of compounds 28c, 11a and 22a.

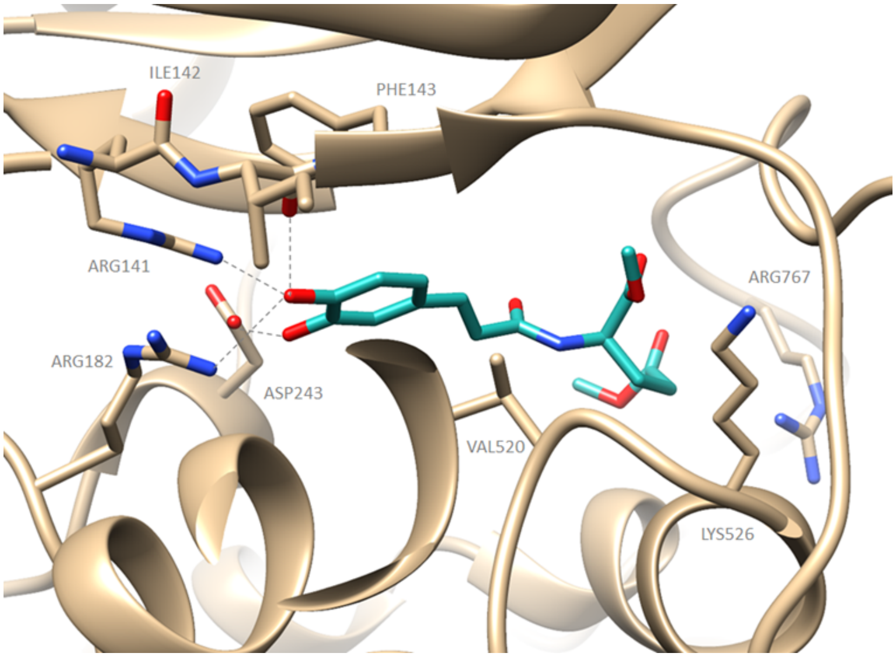

Figure 1 - Preferred docking pose of compound 28c depicted in light sea green bound on SLOX-1 (PDB ID: 3PZW). Blue coloring refers to nitrogen atoms and red to oxygen atoms.

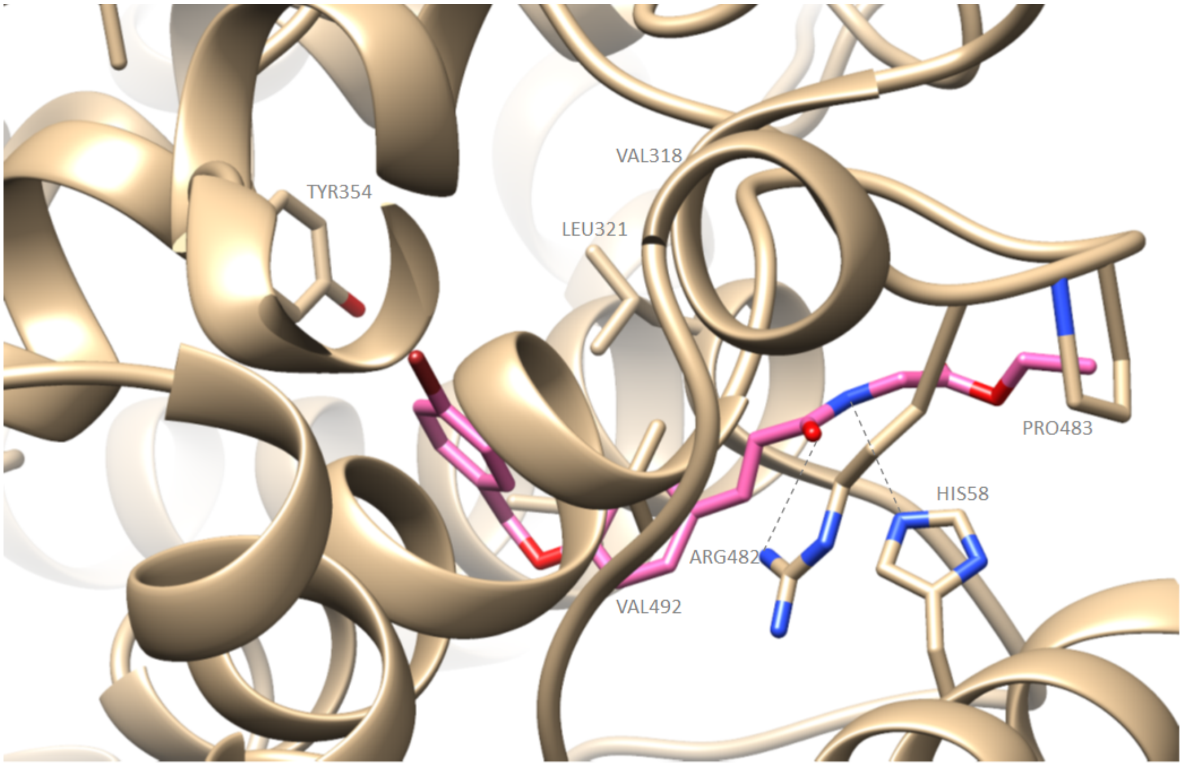

Figure 2 - Preferred docking pose of compound 11a (depicted in pink) bound to COX-2 (PDB IS: 1CX2). Blue coloring refers to nitrogen at-oms and red to oxygen atoms.

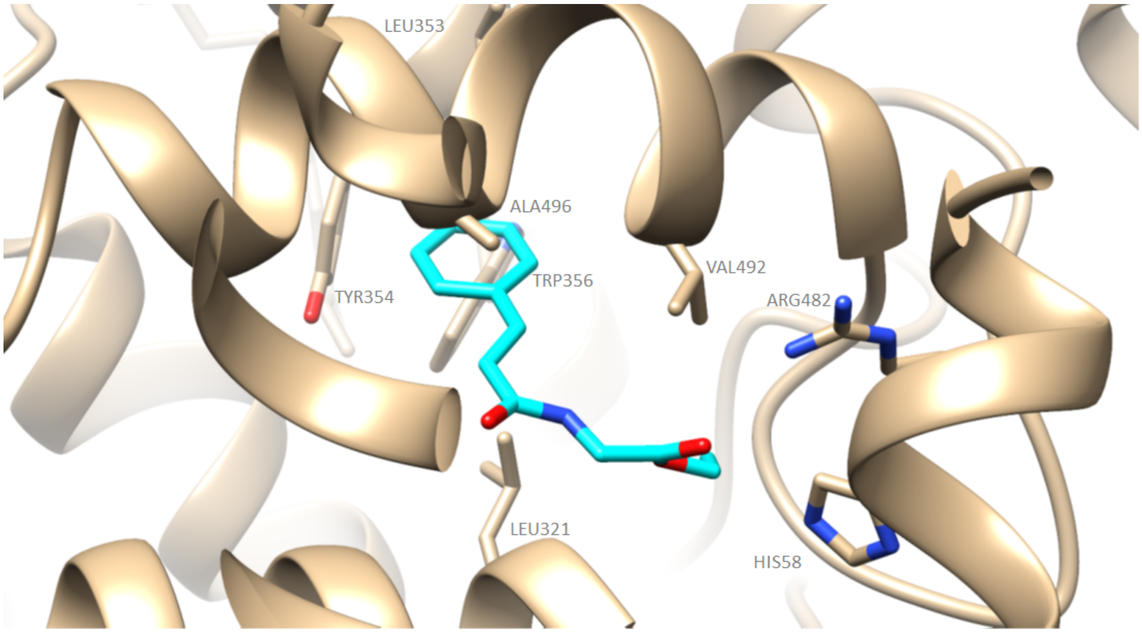

Figure 3 - Preferred docking pose of compound 22a depicted in cyan bound to COX-2 (PDB IS: 1CX2). Blue coloring refers to nitrogen atoms and red to oxygen atoms.

3. Analytical and spectroscopic data of starting materials and final compounds.

3.1. Analytical and spectroscopic data of cinnamic acids 11-20, 29, 30

11

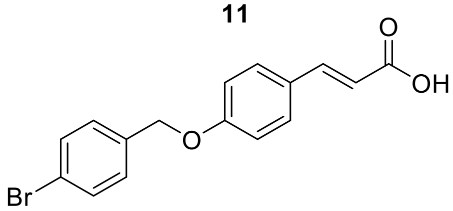

(*E*)-3-(4-((4-bromobenzyl)oxy)phenyl) acrylic acid (11): Starting from 4-((4-bromobenzyl)oxy)benzaldehyde (1). White solid. Yield: 76%, m.p.: 228-230°C (recrystallized from H_2O) [39]. ^{1}H-NMR (500 MHz, DMSO-d$_6$) δ 7.62 (d, *J* = 8.8 Hz, 2H), 7.58 (d, J = 8.3 Hz, 2H), 7.51 (d, *J* = 16.0 Hz, 1H), 7.41 (d, *J* = 8.3 Hz, 2H), 7.03 (d, *J* = 8.8 Hz, 2H), 6.36 (d, *J* = 16.0 Hz, 1H), 5.13 (s, 2H). ^{13}C-NMR (125 MHz, DMSO-d$_6$): δ 167.8, 159.8, 143.6, 136.2, 131.4, 129.9, 129.8, 127.2, 121.0, 116.7, 115.2, 68.5. LC – MS (ESI) m/z: [M+H]$^+$ = 334.

12

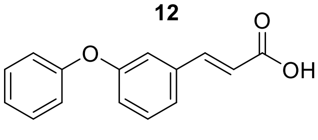

(*E*)-3-(3-phenoxyphenyl)acrylic acid (12): Starting from 3-phenoxybenzaldehyde (2). White solid, Yield: 85%. m.p.: 110-112°C (recrystallized for H_2O) [40]. ^{1}H-NMR: (500 Hz, $CDCl_3$): δ 7.75 (d, *J* = 15.6 Hz, 1H), 7.37-7.40 (m, 4H), 7.01-7.17 (m, 5H), 6.39 (d, *J* = 15.6 Hz, 1H). LC – MS (ESI, m/z): [M+H]$^+$ = 241.

13

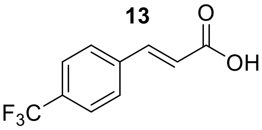

(*E*)-3-(4-(trifluoromethyl)phenyl)acrylic acid (13): Starting from 4-(trifluoro)-methylbenzaldehyde(3). White solid. Yield: 84%. m.p.: 230-232°C (recrystallized from PS) [41]. ^{1}H NMR (500 MHz, DMSO-d$_6$): δ 7.90 (d, *J* = 8.1 Hz, 2H), 7.75 (d, *J* = 8.2 Hz, 2H), 7.65 (d, *J* = 16.1 Hz, 1H), 6.67 (d, *J* = 16.1 Hz, 1H). LC – MS (ESI, m/z):

14

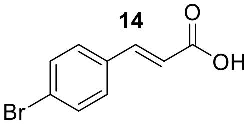

[M+H]$^+$ = 217.

(*E*)-3-(4-bromophenyl) acrylic acid (14): Starting from 4-bromobenzaldehyde (4). White solid. Yield: 74%. m.p.: 254-256°C (recrystallized from PS) [43]. ^{1}H – NMR (500 MHz,$CDCl_3$): δ 7.69 (d, *J* = 16.0 Hz, 1H), 7.54 (d, *J* = 8.2 Hz, 2H), 7.41 (d, *J* = 8.2 Hz, 2H), 6.44 (d, *J* = 16.0 Hz, 1H). LC – MS (ESI, m/z): [M+H]$^+$ = 229.

15

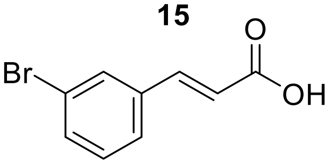

(*E*)-3-(3-bromophenyl) acrylic acid (15): Starting from 3-bromobenzaldehyde (5). White solid. Yield: 70%. m.p.: 178-180°C (recrystallized from PS) [42]. ^{1}H-NMR (500 MHz, DMSO-d$_6$): δ 7.91 (s, 1H), 7.69 (d, *J* = 7.7 Hz, 1H), 7.59 (d, *J* = 8.0 Hz, 1H), 7.53 (d, *J* = 16.0 Hz, 1H), 7.36 (t, *J* = 7.9 Hz, 1H), 6.59 (d, *J* = 16.0 Hz, 1H). LC – MS (ESI, m/z): [M+H]$^+$ = 229.

16

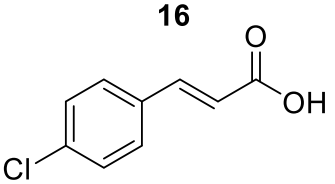

(*E*)-3-(4-chlorophenyl) acrylic acid (16): Starting from 4-chlorobenzaldehyde (6). White solid. Yield: 85%. m.p.: 247-249°C (dec) (recrystallized from PS) [44]. ^{1}H-NMR (DMSO-d$_6$, 500 MHz): δ 7.46 (d, *J* = 16 Hz, 1H), 7.36 (d,

17

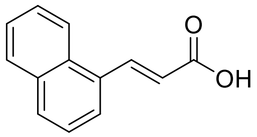

J = 8.2 Hz, 2H), 7.23 (d, *J* = 8.2 Hz, 2H), 6.27 (d, *J* = 16 Hz, 1H). LC-MS (ESI, m/z): [M+H]$^+$ = 183.

(*E*)-3-(naphthalen-1-yl) acrylic acid (17): Starting from 1-naphthaldehyde (7). Off-white solid. Yield: 79%. m.p.: 210 – 212°C (recrystallized from H_2O) [44]. ^{1}H-NMR (500 MHz, CDCl$_3$): δ 8.24 (d, *J* = 15.7 Hz, 1H), 7.95 (d, *J* = 8.3 Hz, 1H), 7.65 (t, *J* = 7.3 Hz, 2H), 7.53 (d, *J* = 7.2 Hz, 1H), 7.35 – 7.24 (m, 3H), 6.27 (d, *J* = 15.7 Hz, 1H). LC – MS (ESI, m/z): [M-H]$^-$ = 197.

18

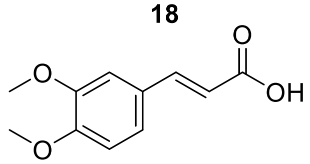

(*E*)-3-(3,4-dimethoxyphenyl) acrylic acid (18): Starting from 3,4-dimethoxybenzaldehyde (8). White solid. Yield: 90%. m.p.: 180-182°C (recrystallized from H_2O) [45]. ^{1}H-NMR (500 MHz, CDCl$_3$): 7.75 (d, *J* = 15.9 Hz, 1H), 7.11 (dd, *J* = 8.3, 2.0 Hz), 7.08 (d, *J* = 2.0 Hz, 1H), 6.91 (d, *J*= 8.3 Hz, 1H), 6.33 (d, *J* = 15.9 Hz, 1H), 3.95 (s, 6H). LC – MS (ESI, m/z): [M+Na]$^+$ = 231.

19

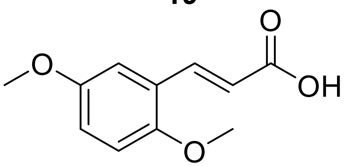

(*E*)-3-(2,5-dimethoxyphenyl) acrylic acid (19): Starting from 2,5-dimethoxybenzaldehyde (9). Light yellow solid. Yield: 82%. m.p.: 148-150 °C (recrystallized from H_2O) [42]. .^{1}H-NMR (500 MHz,CDCl$_3$): δ 8.08 (d, *J* = 16.1 Hz, 1H), 7.07 (d, *J* = 3.0 Hz, 1H), 6.94 (dd, *J* = 9.0, 3.0 Hz, 1H), 6.87 (d, *J* = 9.0 Hz, 1H), 6.52 (d, *J* = 16.1 Hz, 1H), 3.86 (s, 3H), 3.80 (s, 3H). LC – MS (ESI, m/z): [M+H]$^+$ = 209.

20

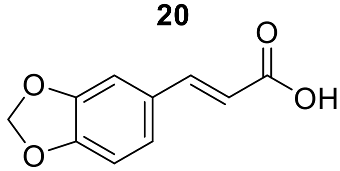

(*E*)-3-(benzo[d][1,3]dioxol-5-yl) acrylic acid (20): Starting from benzo[d][1,3]dioxole-5-carbaldehyde (10). White solid. Yield: 100%. m.p.: 245-247 °C (recrystallized from PS) [45]. ^{1}H-NMR (500 MHz, DMSO-d$_6$): δ 12.19 (s, 1H), 7.48 (d, *J* = 15.9 Hz, 1H), 7.30 (d, *J* = 1.5 Hz, 1H), 7.11 (dd, *J* = 8.1, 1.6 Hz, 1H), 6.90 (d, *J* = 8.0 Hz, 1H), 6.35 (d, *J* = 15.9 Hz, 1H), 6.05 (s, 2H). LC – MS (ESI, m/z): [M+H]$^+$ = 193.

29

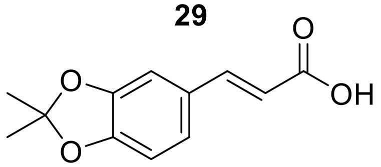

(E)-3-(2,2-dimethylbenzo[d][1,3]dioxol-5-yl)acrylic acid (29): Starting from methyl (E)-3-(2,2-dimethylbenzo[d][1,3]dioxol-5-yl)acrylate. Yellow solid. Yield: 69%. m.p.: 142-145°C (crystallized from 1PS:1EA) [45]. : ^{1}H-NMR (500 MHz, $CDCl_3$): δ 7.68 (d, *J* = 15.8 Hz, 1H), 6.98 (d, *J* = 10.5 Hz, 2H), 6.73 (d, *J* = 7.8 Hz, 1H), 6.26 (d, *J* = 15.8 Hz, 1H), 1.69 (s, 6H). LC – MS (ESI, m/z): $[M+Na]^+$ = 243.

30

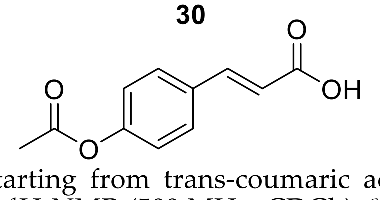

(E)-3-(4-acetoxyphenyl)acrylic acid (30): Starting from trans-coumaric acid (25). White solid. Yield: 100%. m.p.: 123-125°C (crystallized from EA) [46]. ^{1}H-NMR (500 MHz, $CDCl_3$): δ 7.77 (d, *J* = 15.9 Hz), 7.57 (d, *J* = 8.5 Hz), 7.15 (d, *J* = 8.5 Hz), 6.41 (d, *J* = 16.0 Hz), 2.32 (s, 3H). LC – MS (ESI, m/z): $[M+H]^+$ = 206.

3.2. Analytical and spectroscopic data for the amino acid cinnamates 11a-30a, 11b-30b, 11c-30c.

11a

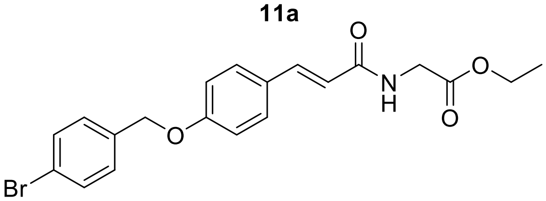

ethyl (E) - (3 - (4 - ((4 - bromobenzyl) oxy) phenyl) acryloyl) glycinate (11a): Starting from (E)-3-(4-((4-bromobenzyl)oxy)phenyl) acrylic acid (11). Off-white solid. Yield: 78%. m.p.: 157-160°C (PS). IR (KBr, cm^{-1}): 3350.4, 1742.6, 1731.0. ^{1}H – NMR (250 MHz, $CDCl_3$): δ 7.60 (d, *J* = 15.6 Hz, 1H), 7.52 (d, *J* = 8.3 Hz, 2H), 7.46 (d, *J* = 8.6 Hz, 2H), 7.30 (d, *J* = 8.2 Hz, 2H), 6.94 (d, *J* = 8.6 Hz, 2H), 6.33 (d, *J* = 15.6 Hz, 1H), 6.09 (brs, 1H), 5.04 (s, 2H), 4.25 (q, *J* = 7.1 Hz, 2H), 4.17 (d, *J* = 5.0 Hz, 2H), 1.31 (t, *J* = 7.1 Hz, 3H). ^{13}C-NMR (63 MHz, $CDCl_3$): δ 170.3, 166.3, 160.1, 141.5, 135.7, 131.9, 129.7, 129.2, 128.0, 122.2, 117.8, 115.3, 69.5, 61.8, 41.8, 14.3. HRMS (ESI) *m/z:* $[M+H]^+$ Calculated for $C_{20}H_{21}BrNO_4$ 418.0648 Found 418.0647.

12a

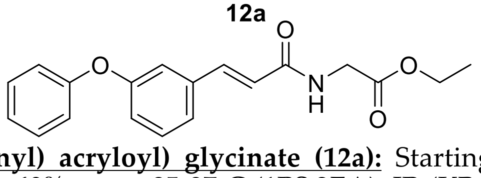

ethyl (E) - (3 - (3 - phenoxyphenyl) acryloyl) glycinate (12a): Starting from (*E*)-3-(3-phenoxyphenyl) acrylic acid (12). White solid. Yield: 60%. m.p.: 85-87°C (1PS:2EA). IR (KBr,cm^{-1}): 3357.0, 1765.0, 1658.7. ^{1}H-NMR (500 MHz, Acetone-d_6): δ 7.62 (brs, 1H), 7.54 (d, *J* = 15.7 Hz, 1H), 7.43 – 7.38 (m, 3H), 7.36 (d, *J* = 7.7 Hz, 1H), 7.26 – 7.23 (m, 1H), 7.18 – 7.14 (m, 1H), 7.06 – 7.03 (m, 2H), 7.01 (ddd, *J* = 8.1, 2.4, 0.9 Hz, 1H), 6.76 (d, *J* = 15.7 Hz, 1H), 4.15 (q, *J* = 7.1 Hz, 2H), 4.06 (d, *J* = 4.9 Hz, 2H), 1.23 (t, *J* = 7.1 Hz, 3H). ^{13}C-NMR (125 MHz, Acetone-d_6): δ 170.5, 166.1, 166.0, 158.7, 157.9, 140.2, 138.1, 131.2, 130.8, 124.5, 123.9, 123.00, 120.6, 119.7, 118.1, 61.4, 41.9, 41.7, 14.5. HRMS (ESI) *m/z:* $[M+Na]^+$ Calculated for $C_{19}H_{19}NO_4Na$: 348.1212 Found

13a

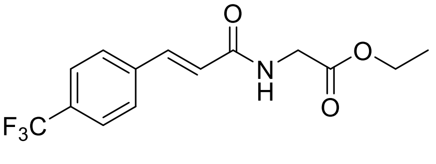

348.1210.

ethyl (E)-(3-(4-(trifluoromethyl) phenyl) acryloyl) glycinate (13a): Starting from (E)-3-(4-(trifluoromethyl) phenyl)acrylic acid (13). White solid. Yield: 67%. m.p.: 138-140°C (1PS:2EA). IR(KBr, cm^1): 3373.6, 1732.1,

1679.7. ^{1}H – NMR (500 MHz, $CDCl_3$): δ 7.67 (d, *J* = 15.6 Hz, 1H), 7.61 (q, *J* = 8.3 Hz, 4H), 6.55 (d, J = 15.6 Hz, 1H), 6.30 (brs, 1H), 4.26 (q, *J* = 7.1 Hz, 2H), 4.18 (d, *J* = 4.6 Hz, 2H), 1.31 (t, *J* = 7.0 Hz). ^{13}C – NMR (125 MHz, $CDCl_3$) δ: 170.1, 166.3, 140.3, 138.2 (d, *J*=1.25 Hz), 131.6 (d, *J*=32.5 Hz), 128.2, 126.0 (q, *J*=3.8 Hz), 124.1 (d, *J*=271.3 Hz), 61.9, 41.8, 14.3. HRMS (ESI) *m/z:* [M+H]$^+$ Calculated for $C_{14}H_{15}F_3NO_3$ 302.0999 Found 302.1001.

14a

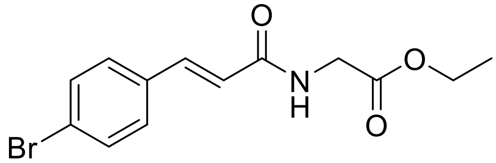

ethyl (E)-(3-(4-bromophenyl)acryloyl)glycinate (14a): Starting from (E)-3-(4-bromophenyl)acrylic acid (14). White solid. Yield: 95%. m.p.: 101-103°C (1PS:2EA). IR (KBr, cm^{-1}): 3232.4, 1737.0, 1655.9. ^{1}H – NMR (500 MHz, $CDCl_3$): δ 7.51 (d, *J* = 15.6 Hz, 1H), 7.41 (d, *J* = 8.2 Hz, 2H), 7.28 (d, J = 8.2 Hz, 2H), 6.78 (brs, 1H), 6.49 (d, *J* = 15.6 Hz, 1H), 4.18 (q, *J* = 7.1 Hz, 2H), 4.12 (d, *J* = 5.2 Hz, 2H), 1.25 (t, *J* = 7.1 Hz, 3H). ^{13}C – NMR (125 MHz, $CDCl_3$): δ 170.2, 166.0, 140.4, 133.6, 132.0, 129.3, 124.0, 120.7, 61.7, 41.7, 14.2. HRMS (ESI) *m/z:* [M+H]$^+$ Calculated for $C_{13}H_{15}BrNO_3$: 312.0230/314.0210 Found 312.0236/314.0214 (1:1).

ethyl (E)-(3-(3-bromophenyl)acryloyl)glycinate (15a): Starting from (E)-3-(3-bromophenyl)acrylic acid (15). White solid. Yield: 71%. m.p.: 88-90°C (1PS:2EA). IR (KBr, cm^{-1}): 3333.3, 1739.8, 1661.5. ^{1}H-NMR (500 MHz, $CDCl_3$): δ 7.59 (s, 1H), 7.52 (d, *J* = 15.6 Hz, 1H), 7.43 (d, *J* = 7.9 Hz, 1H), 7.36 (d, *J* = 7.6 Hz, 1H), 7.19 (t, *J* = 7.8 Hz, 1H), 6.66 (brs, 1H), 6.50 (d, *J* = 15.6 Hz, 1H), 4.21 (q, *J* = 7.1 Hz, 2H), 4.15 (d, *J* = 5.2 Hz, 2H), 1.27 (t, *J* = 7.1 Hz, 3H). ^{13}C-NMR (125 MHz, $CDCl_3$) δ: 170.1, 165.7, 140.2, 136.9, 132.7, 130.5, 130.4, 126.7, 123.0, 121.4, 61.8, 41.7, 14.2. HRMS (ESI) *m/z*: [M+H]$^+$ Calculated for $C_{13}H_{15}BrNO_3$: 312.0230/314.0210 Found 312.0236/314.0215

15a

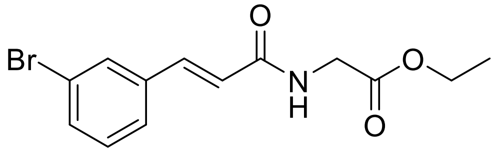

(1:1).

16a

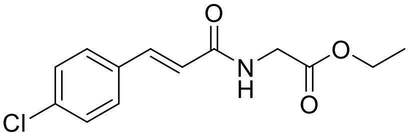

ethyl (E)-(3-(4-chlorophenyl)acryloyl)glycinate (16a): Starting from (E)-3-(4-chlorophenyl)acrylic acid (16). White solid. Yield: 98%. m.p.: 112-114°C (PS). IR (KBr, cm^{-1}): 3254.9, 1737.0 1655.9. ^{1}H–NMR (500 MHz, $CDCl_3$): δ 7.59 (d, *J* = 15.6 Hz, 1H), 7.43 (d, *J* = 8.5 Hz, 2H), 7.34 (d, *J* = 8.5 Hz, 2H), 6.44 (d, *J* = 15.6 Hz, 1H), 6.25 (brs, 1H), 4.25 (q, *J* = 7.1 Hz, 2H), 4.17 (d, *J* = 5.1 Hz, 2H), 1.30 (t, *J* = 7.2 Hz, 3H). ^{13}C-NMR (125 MHz, $CDCl_3$): δ 170.2, 165.7, 140.6, 135.8, 133.3, 129.2, 129.1, 120.5, 61.8, 41.8, 14.3. HRMS (ESI) *m/z*: [M+H]$^+$

17a

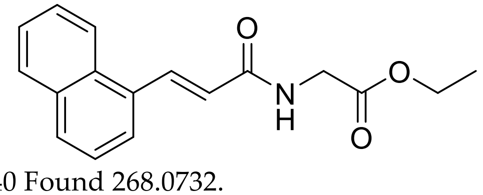

Calculated for $C_{13}H_{15}ClNO_3$ 268.0740 Found 268.0732.

ethyl (E) - (3 - (naphthalen-1-yl) acryloyl) glycinate (17a): Starting from (E)-3-(naphthalen-1-yl)acrylic acid (17). White solid. Yield: 99%. m.p.: 142-144°C (PS). IR (KBr, cm^{-1}): 3299.7, 1737.0, 1647.5. ^{1}H – NMR (500 MHz, $CDCl_3$): δ 8.49 (d, *J* = 15.4 Hz, 1H), 8.21 (d, *J* = 8.3 Hz, 1H), 7.87 (dd, *J* = 7.8, 3.3 Hz, 2H), 7.72 (d, *J* = 7.2 Hz, 1H), 7.54 (ddd, *J* = 14.9, 13.7, 6.8 Hz, 2H), 7.48 (t, *J* = 7.7 Hz, 1H), 6.55 (d, *J* = 15.3 Hz, 1H), 6.23 (brs, 1H),

4.27 (q, *J* = 7.1 Hz, 2H), 4.22 (d, *J* = 5 Hz, 2H), 1.32 (t, *J* = 7.1 Hz, 3H). ^{13}C – NMR (125 MHz, $CDCl_3$) δ: 170.3, 166.0, 139.0, 133.7, 132.3, 131.6, 130.1, 128.7, 126.8, 126.2, 125.5, 124.8, 123.7, 122.8, 61.7, 41.8, 14.3. HRMS (ESI) *m/z:* $[M+H]^+$ Calculated for $C_{17}H_{18}NO_3$ 284.1286 Found 284.1283.

18a

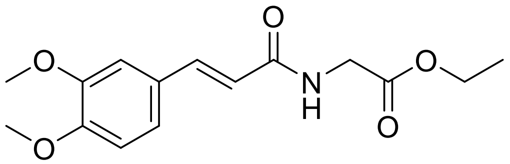

ethyl (E) - (3 - (3,4 -dimethoxyphenyl) acryloyl) glycinate (18a): Starting from (E)-3-(3,4-dimethoxyphenyl) acrylic acid (18). Yellow solid. Yield: 55%. m.p.: 110-112°C (2PS:1EA). IR (KBr, cm^{-1}): 3265.8, 1745.8, 1653.8. ^{1}H – NMR (500 MHz, $CDCl_3$): δ 7.51 (d, *J* = 15.6 Hz, 1H), 6.98 (dd, *J* = 8.3, 1.8 Hz, 1H), 6.93 (d, *J* = 1.8 Hz, 1H), 6.74 (d, *J* = 8.3 Hz, 1H), 6.68 (t, *J* = 5.0 Hz, 1H), 6.36 (d, *J* = 15.6 Hz, 1H), 4.16 (q, *J* = 7.1 Hz, 2H), 4.11 (d, *J* = 5.3 Hz, 2H), 3.82 (s, 3H), 3.80 (s, 3H), 1.23 (t, *J* = 7.1 Hz, 3H). ^{13}C-NMR (125 MHz, $CDCl_3$): δ 170.3, 166.4, 150.6, 149.0, 141.6, 127.6, 122.1, 118.0, 111.0, 109.9, 61.5, 55.9, 41.6, 14.2. HRMS (ESI) *m/z:* $[M+H]^+$ Calculated for

19a

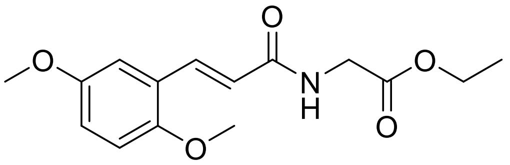

$C_{15}H_{20}NO_5$ 294.1336 Found 294.1340.

ethyl (E) - (3 - (2,5 - dimethoxyphenyl) acryloyl) glycinate (19a): Starting from (E)-3-(2,5-dimethoxyphenyl) acrylic acid (19). White solid. Yield: 74%. m.p.: 141-142°C (1PS:2EA). IR (KBr, cm^{-1}): 3260.7, 1740.7, 1652.7. ^{1}H – NMR (500 MHz, $CDCl_3$): δ 7.85 (d, *J* = 15.8 Hz, 1H), 7.02 (d, *J* = 2.9 Hz, 1H), 6.88 (dd, *J* = 8.9, 2.9 Hz, 1H), 6.84 (d, *J* = 9.0 Hz, 1H), 6.58 (d, *J* = 15.8 Hz, 1H), 6.15 (brs, 1H), 4.25 (q, *J* = 7.1 Hz, 2H), 4.18 (d, *J* = 5.0 Hz, 2H), 3.84 (s, 3H), 3.79 (s, 3H), 1.31 (t, *J* = 7.2 Hz, 3H). ^{13}C-NMR (125 MHz, $CDCl_3$) δ 170.3, 166.6, 153.5, 152.9, 137.1, 124.3, 121.1, 116.5, 113.9, 112.4, 61.7, 56.1, 55.9, 41.7, 14.3. HRMS (ESI) *m/z*: $[M+H]^+$ Calculated for $C_{15}H_{19}NO_5$ 294.1336 Found 294.1337.

20a

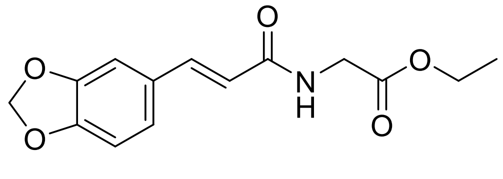

ethyl (E) - (3- (benzo[d][1,3]dioxol – 5 -yl) acryloyl) glycinate (20a): Starting from (E)-3-(benzo[d][1,3]dioxol-5-yl)acrylic acid (20). White solid. Yield: 62%. m.p.: 135-137°C (1PS: 2EA) [lit[48]. 135.5°C. ^{1}H – NMR (500 MHz, $CDCl_3$): δ 7.56 (d, *J* = 15.5 Hz, 1H), 7.01 (d, *J* = 1.4 Hz, 1H), 6.99 (dd, *J* = 8.0, 1.4 Hz, 1H), 6.80 (d, *J* = 8.0 Hz, 1H), 6.29 (d, *J* = 15.5 Hz, 1H), 6.12 (s, 1H), 6.00 (s, 2H), 4.25 (q, *J* = 7.1 Hz, 2H), 4.17 (d, *J* = 5.1 Hz, 2H), 1.30 (t, *J* = 7.1 Hz, 3H). ^{13}C – NMR (125 MHz, $CDCl_3$): δ 170.3, 166.2, 149.3, 148.3, 141.7, 129.2, 124.2, 117.9, 108.6, 106.5, 101.6, 61.8, 41.8, 14.3. HRMS (ESI) *m/z:* $[M+H]^+$ Calculated for $C_{14}H_{16}NO_5$ 278.1023 Found 278.1026.

21a

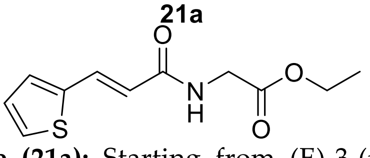

ethyl (E)-(3-(thiophen-2-yl)acryloyl)glycinate (21a): Starting from (E)-3-(thiophen-2-yl)acrylic acid (21). Off-white solid. Yield: 70% m.p.: 92-94°C (PS). IR (KBr, cm^{-1}): 2294.3, 1678.3. ^{1}H - NMR (500 MHz, $CDCl_3$): δ 7.77 (d, *J* = 15.3 Hz, 1H), 7.33 (d, *J* = 4.9 Hz, 1H), 7.23 (d, *J* = 3.4 Hz, 1H), 7.06 – 7.02 (m, 1H), 6.27 (d, J = 15.3 Hz, 1H), 4.25 (dd, J_1 = 14.0 Hz, J_2 = 6.8 Hz, 2H), 4.16 (d, *J* = 4.6 Hz, 2H), 1.31 (t, *J* = 7.2 Hz, 3H). ^{13}C – NMR (125 MHz, $CDCl_3$): δ 170.2, 165.8, 139.9, 134.5, 130.6, 128.1, 127.1, 118.8, 61.7, 41.7, 14.3. HRMS (ESI) *m/z:*

22a

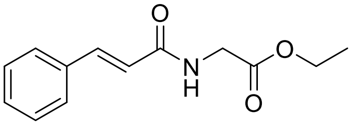

$[M+H]^+$ Calculated for $C_{11}H_{14}NO_3S$ 240.0689 Found 240.0694.

ethyl cinnamoyl glycinate (22a): Starting from trans-cinnamic acid (22). White solid. Yield: 66%. m.p.: 102-104°C (PS) [lit [49]. 88-89°C]. IR (KBr, cm^{-1}): 3355.7, 1725.8, 1667.1. ^{1}H – NMR (500 MHz, $CDCl_3$): δ 7.66 (d, *J* = 15.6 Hz, 1H), 7.51 (dd, *J* = 7.4, 1.9 Hz, 2H), 7.40 – 7.34 (m, 3H), 6.47 (d, *J* = 15.6 Hz, 1H), 6.14 (brs, 1H), 4.26 (q, *J* = 7.1Hz, 2H), 4.18 (d, *J* = 4.4 Hz, 2H), 1.31 (t, *J* = 7.1 Hz, 3H). HRMS (ESI) *m/z:* [M+H]+ Calculated for $C_{13}H_{16}NO_3$ 234.1130 Found 234.1127.

ethyl ((2E,4E)-5-phenylpenta-2,4-dienoyl)glycinate (23a): Starting from (2E,4E)-5-phenylpenta-2,4-dienoic acid (23). White solid. Yield: 47%. m.p.: 108-110°C (2PS:1EA). IR (KBr, cm^{-1}): 3316.9, 1743.1, 1645.7. ^{1}H – NMR (500 MHz, $CDCl_3$): δ 7.45 – 7.39 (m, 3H), 7.32 (t, *J* = 7.3 Hz, 2H), 7.30 – 7.27 (m, 1H), 6.85 (d, *J* = 5.4 Hz, 2H), 6.38 (brs, 1H), 6.06 (d, *J* = 14.9 Hz, 1H), 4.23 (q, *J* = 7.1 Hz, 2H), 4.15 (d, *J* = 5.2 Hz, 2H), 1.29 (t, *J* = 7.1 Hz, 3H). ^{13}C -NMR (125 MHz, $CDCl_3$): δ 170.2, 166.1, 141.7, 139.6, 136.2, 128.8, 128.7, 127.0, 126.2, 123.1, 61.6,

23a

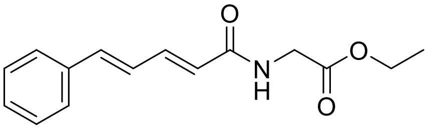

24a

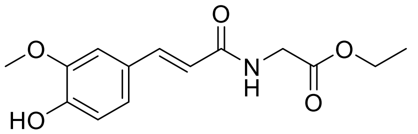

41.6, 14.1. HRMS (ESI) *m/z*: $[M+Na]^+$ Calculated for $C_{15}H_{17}NO_3Na$ 282.1101 Found 282.1105.

ethyl (E) - (3- (4- hydroxy-3-methoxyphenyl) acryloyl) glycinate (24a): Starting from trans-ferulic acid (24). Off-yellow solid. Yield: 38%. m.p.: 132-134°C (PS) [50]. IR (KBr, cm^{-1}): 3320.7, 1724.8, 1664.1. ^{1}H – NMR (500 MHz, Acetone-d_6): δ 8.03 (s, 1H), 7.48 (d, *J* = 15 Hz, 1H), 7.49 (s, 1H), 7.20 (d, *J* = 1.8 Hz, 1H), 7.07 (dd, *J* = 8.2, 1.8 Hz, 1H), 6.84 (d, *J* = 8.1 Hz, 1H), 6.62 (d, *J* = 15.6 Hz, 1H), 4.15 (q, *J* = 7.1 Hz, 2H), 4.06 (d, *J* = 5.9 Hz, 2H), 3.88 (s, 3H), 1.23 (t, *J* = 7.1 Hz, 3H). ^{13}C – NMR (125 MHz, Acetone-d_6): δ 170.7, 166.8, 149.3, 148.6, 141.3, 128.1, 122.8, 119.2, 116.1, 111.33, 61.3, 56.2, 41.8, 14.5. HRMS (ESI) *m/z*: $[M+H]^+$ Calculated for $C_1H_{18}NO_5$ 280.1180 Found 280.1187

25a

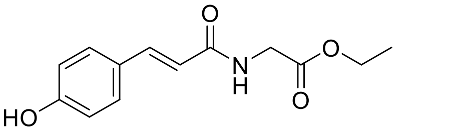

<u>**ethyl (E)-(3-(4-hydroxyphenyl)acryloyl)glycinate (25a):**</u> Starting from trans-coumaric acid (25). White solid. Yield: 58%. m.p.: 167-169°C (1PS:2EA) [51]. IR (KBr, cm^{-1}): 3327.7, 1725.8, 1661.5. ^{1}H-NMR (500 MHz, Acetone-d_6): δ 8.78 (s, 1H), 7.50 (d, *J* = 15.6 Hz, 1H), 7.46 (d, *J* = 8.6 Hz, 2H), 6.87 (d, *J* = 8.6 Hz, 2H), 6.59 (d, *J* = 15.7 Hz, 1H), 4.15 (q, *J* = 7.1 Hz, 2H), 4.06 (d, *J* = 5.9 Hz, 2H), 2.90 (s, 1H), 1.23 (t, *J* = 7.1 Hz, 3H). HRMS (ESI) *m/z*: $[M+H]^+$ Calculated for $C_{13}H_{16}NO_4$ 250.1074 Found 250.1077.

26a

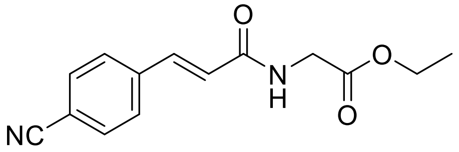

<u>**ethyl (E)- (3- (4-cyanophenyl) acryloyl) glycinate (26a):**</u> Starting from (E)-3-(4-cyanophenyl)acrylic acid (26). White solid. Yield: 50%. m.p.: 158-160°C (PS). IR (KBr, cm^{-1}): 3263.5, 1737.0, 1653.1. ^{1}H – NMR (500 MHz, DMSO-d_6): δ 8.62 (t, *J* = 5.8 Hz, 1H), 7.88 (d, *J* = 8.3 Hz, 2H), 7.78 (d, *J* = 8.3 Hz, 2H), 7.51 (d, *J* = 15.9 Hz, 1H), 6.86 (d, *J* = 15.9 Hz, 1H), 4.11 (q, *J* = 7.1 Hz, 2H), 3.98 (d, *J* = 5.9 Hz, 2H), 1.20 (t, *J* = 7.1 Hz, 3H). ^{13}C – NMR (125 MHz, DMSO-d_6): δ 169.8, 164.9, 139.4, 137.6, 132.8, 128.3, 124.9, 118.7, 111.6, 60.5, 41.0, 14.1. HRMS (ESI) *m/z:* $[M+Na]^+$ Calculated for $C_{14}H_{14}N_2O_3Na$ 281.0980 Found 281.0984.

27a

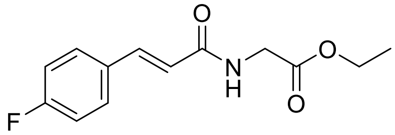

<u>**ethyl (E)- (3- (4- fluorophenyl) acryloyl) glycinate (27a):**</u> Starting from (E)-3-(4-fluorophenyl)acrylic acid (27). White solid. Yield: 46%. m.p.: 101-102°C (PS). IR (KBr, cm^{-1}): 3260.5, 1737.0 1658.7. ^{1}H - NMR (500 MHz, $CDCl_3$): δ 7.60 (d, *J* = 15.6 Hz, 1H), 7.49 – 7.44 (m, 2H), 7.04 (t, *J* = 8.6 Hz, 2H), 6.39 (d, J = 15.6 Hz, 1H), 6.31 (s, 1H), 4.24 (q, *J* = 7.1 Hz, 2H), 4.17 (d, *J* = 5.1 Hz, 2H), 1.30 (t, *J* = 7.1 Hz, 3H).^{13}C – NMR (125 MHz, $CDCl_3$): δ 170.3, 166.9, 163.8 (d, *J*=248.8 Hz), 140.7, 131.0 (d, *J* = 3.8 Hz), 129.8 (d, *J* = 2.5 Hz), 116.1 (d, *J* = 21.3

28a

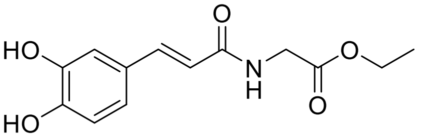

Hz), 61.8, 41.8, 14.3. HRMS (ESI) *m/z*: $[M+H]^+$ Calculated for $C_{13}H_{15}FNO_3$ 252.1036 Found 252.1029.

<u>**ethyl (E) - (3 - (3,4 – dihydroxyphenyl) acryloyl) glycinate (28a):**</u> Starting from trans-caffeic acid (28). Yellow solid. Yield: 31%. m.p.: 170-171°C (1PS:5EA) [51] ^{1}H – NMR (500 MHz, Acetone-d_6): δ 7.47 (brs, 1H), 7.43 (d, *J* = 15.6 Hz, 1H), 7.09 (d, *J* = 2.0 Hz, 1H), 6.96 (dd, J_1 = 8.2 Hz, J_2 = 2.0 Hz, 1H), 6.84 (d, *J* = 8.1 Hz, 1H), 6.53 (d, *J* = 15.6 Hz, 1H), 4.15 (q, *J* = 7.1 Hz, 2H), 4.05 (d, *J* = 5.9 Hz, 2H), 2.94 (s, 1H), 2.78 (s, 1H), 1.23 (t, *J* = 7.1 Hz, 3H). HRMS (ESI) *m/z:* $[M+H]^+$ Calculated for $C_{13}H_{16}NO_5$ 266.2689 Found 266.2694.

29a

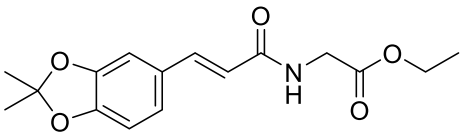

ethyl (E) - (3- (2,2-dimethylbenzo[d][1,3]dioxol-5-yl) acryloyl) glycinate (29a): Starting from (E)-3-(2,2-dimethylbenzo[d][1,3]dioxol-5-yl)acrylic acid (29). Oil. Yield: 75%. ^{1}H – NMR (500 MHz, $CDCl_3$) :δ 7.51 (d, *J* = 15.5 Hz, 1H), 6.89 (d, *J* = 9.7 Hz, 2H), 6.64 (d, *J* = 7.8 Hz, 1H), 6.56 (brs, 1H), 6.29 (d, *J* = 15.5 Hz, 1H), 4.19 (q, *J* = 7.1 Hz, 2H), 4.13 (d, *J* = 5.2 Hz, 2H), 1.64 (s, 6H), 1.25 (t, *J* = 7.2 Hz, 3H). ^{13}C – NMR (125 MHz, $CDCl_3$): δ 170.3, 166.5, 149.1, 148.0, 141.8, 128.5, 123.7, 118.7, 117.5, 108.3, 106.3, 61.6, 41.7, 25.9, 14.2. HRMS (ESI) *m/z:* $[M+Na]^+$ Calculated for $C_{16}H_{19}NO_5$ 328.1155 Found 328.1154.

30a

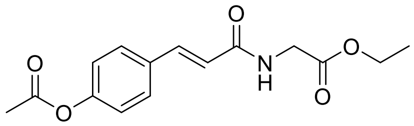

ethyl (E)- (3 - (4- acetoxyphenyl) acryloyl) glycinate (30a): Starting from (E)-3-(4-acetoxyphenyl)acrylic acid (30). White solid. Yield: 59%. m.p.: 98-100ºC (PS). IR (KBr, cm^{-1}): 3366.9, 1742.6, 1678.3. ^{1}H – NMR (500 MHz, $CDCl_3$): δ 7.62 (d, *J* = 15.6 Hz, 1H), 7.51 (d, *J* = 8.3 Hz, 2H), 7,10 (d, *J* = 8.5 Hz, 2H), 6.41 (d, *J* = 15.6 Hz, 1H), 6.22 (brs, 1H), 4.24 (q, J = 7.1 Hz, 2H), 4.17 (d, *J* = 4.8 Hz, 2H), 2.30 (s, 3H), 1.30 (t, *J* = 7.1 Hz, 3H). ^{13}C – NMR (125 MHz, $CDCl_3$) δ: 170.2, 169.3, 166.8, 151.9, 140.9, 132.5, 129.1, 122.2, 120.1, 61.8, 41.8, 21.3, 14.3. HRMS (ESI) *m/z:* $[M+Na]^+$ Calculated for $C_{15}H_{17}NO_5Na$ 314.1005 Found 314.1000.

6b

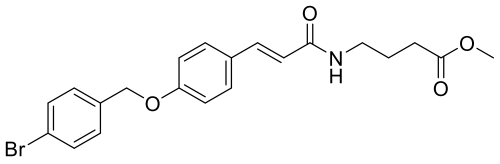

methyl (E)-4-(3-(4-((4-bromobenzyl) oxy) phenyl) acrylamido) butanoate (11b): Starting from (E)-3-(4-((4-bromobenzyl)oxy)phenyl) acrylic acid (11). Yellow solid. Yield: 50%. m.p.: 143-145ºC. (PS). IR (KBr, cm^{-1}): 3310.8, 1733.1, 1651.3. ^{1}H – NMR (250 MHz, $CDCl_3$): δ 7.56 (d, *J* = 15.6 Hz, 1H), 7.52 (d, *J* = 8.3 Hz, 2H), 7.44 (d, *J* = 8.7 Hz, 2H), 7.30 (d, *J* = 8.2 Hz, 2H), 6.93 (d, *J* = 8.7 Hz, 2H), 6.24 (d, *J* = 15.6 Hz, 1H), 5.81 (s, 1H), 5.04 (s, 2H), 3.68 (s, 3H), 3.43 (q, *J* = 6.6 Hz, 2H), 2.42 (t, *J* = 7.1 Hz, 2H), 1.91 (p, *J* = 6.9 Hz, 2H). ^{13}C – NMR (63 MHz, $CDCl_3$): δ 174.2, 166.4, 159.9, 140.7, 135.8, 131.9, 129.5, 129.2, 128.2, 122.2, 118.6, 115.3, 69.4, 51.9, 39.4, 31.8, 24.8. HRMS (ESI) m/z: $[M+H]^+$ Calculated for $C_{21}H_{23}BrNO_4$ 432.0805 Found 432.0808

12b

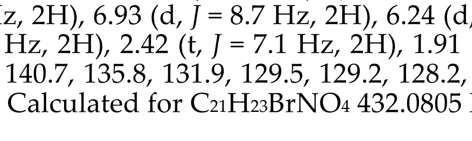

methyl (E) - 4- (3- (3-phenoxyphenyl) acrylamido) butanoate (12b): Starting from (E)-3-(3-phenoxyohenyl)acrylic acid (12). Light brown oil. Yield: 58%. IR (KBr, cm^{-1}): 3349.9, 1722.8. ^{1}H – NMR (500 MHz, $CDCl_3$): δ 7.52 (d, *J* = 15.6 Hz, 1H), 7.30 (t, *J* = 7.9 Hz, 2H), 7.24 (d, *J* = 7.9 Hz, 1H), 7.16 (d, *J* = 7.6 Hz, 1H), 7.10 – 7.06 (m, 2H), 6.96 (d, *J* = 7.8 Hz, 2H), 6.92 (dd, *J* = 8.0, 1.9 Hz, 1H), 6.55 (s, 1H), 6.37 (d, *J* = 15.6 Hz, 1H), 3.61 (s, 3H), 3.37 (dd, *J* = 12.8, 6.6 Hz, 2H), 2.36 (t, *J* = 7.2 Hz, 2H), 1.86 (p, *J* = 7.0 Hz, 2H). ^{13}C-NMR (125 MHz, $CDCl_3$) δ: 174.0, 166.1, 157.7, 156.8, 140.2, 136.7, 130.1, 129.9, 123.6, 123.0, 121.5, 119.9, 119.0, 117.4, 51.7, 39.2, 31.5, 24.7. HRMS (ESI) *m/z*: $[M+Na]^+$ Calculated for $C_{20}H_{21}NO_4Na$ 362.1363 Found 362.1368.

13b

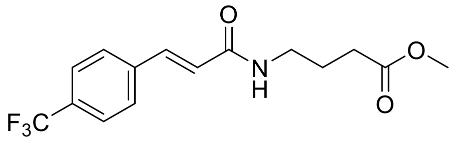

methyl (E) - 4- (3- (4-(trifluoromethyl) phenyl) acrylamido) butanoate (13b): Starting from (E)-3-(4-(trifluoromethyl)phenyl)acrylic acid (13). White solid. Yield: 46%. m.p.: 108-110°C (PS). IR (KBr, cm^{-1}): 3253.4, 1659.2. ^{1}H – NMR (500 MHz, DMSO-d_6): δ 8.22 (t, *J* = 5.6 Hz, 1H), 7.75 (dd, *J* = 8.5 Hz, 3.0 Hz, 4H), 7.48 (d, *J* = 15.8 Hz, 1H), 6.72 (d, *J* = 15.8 Hz, 1H), 3.59 (s, 3H), 3.20 (dd, *J* = 12.8, 6.7 Hz, 2H), 2.35 (t, *J* = 7.4 Hz, 2H), 1.73 (p, *J* = 7.2 Hz, 2H). ^{13}C – NMR (125 MHz, DMSO-d_6) δ: 172.9, 164.4, 139.0 (d, *J*=1.25 Hz), 136.8, 129.2 (d, *J*=32.5 Hz), 128.0, 125.7 (q, *J*=3.8 Hz), 125.3 (q, *J*=271.4 Hz), 124.9, 51.2, 38.0, 30.7, 24.5. HRMS (ESI) *m/z:* $[M+H]^+$ Calculated for $C_{15}H_{17}F_3NO_3$ 316.1160 Found 316.1157.

14b

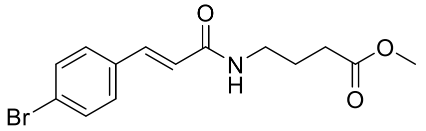

methyl (E) - 4- (3- (4-bromophenyl) acrylamido) butanoate (14b): Starting from (E)-3-(4-bromophenyl)acrylic acid (14). White solid. Yield: 86%. m.p.: 113-115°C (PS). IR (KBr, cm^{-1}): 3282.8, 1654.4. ^{1}H – NMR (500 MHz, $CDCl_3$): δ 7.54 (d, *J* = 15.6 Hz, 1H), 7.48 (d, *J* = 8.5 Hz, 2H), 7.34 (d, *J* = 8.4 Hz, 2H), 6.37 (d, *J* = 15.6 Hz, 1H), 6.08 (brs, 1H), 3.67 (s, 3H), 3.43 (dd, *J* = 12.7, 6.7 Hz, 2H), 2.42 (t, *J* = 7.1 Hz, 2H), 1.91 (p, *J* = 6.9 Hz, 2H). ^{13}C – NMR (125 MHz, $CDCl_3$): δ 174.1, 165.7, 139.7, 133.8, 132.0, 129.2, 121.3, 51.8, 39.3, 31.6, 24.6. HRMS (ESI) *m/z:* $[M+H]^+$ Calculated for $C_{14}H_{17}BrNO_3$ 326.0386 Found 326.0379.

15b

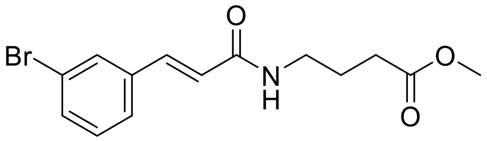

methyl (E) - 4- (3- (3- bromophenyl) acrylamido) butanoate (15b): Starting from (E)-3-(3-bromophenyl)acrylic acid (15). White solid. Yield: 52%. m.p.: 70-72°C (PS). IR (KBr, cm^{-1}): 3305.0, 1653.9. ^{1}H – NMR (500 MHz, $CDCl_3$): δ 7.64 (s, 1H), 7.54 (d, *J* = 15.6 Hz, 1H), 7.46 (d, *J* = 8.0 Hz, 1H), 7.40 (d, *J* = 7.8 Hz, 1H), 7.23 (t, *J* = 7.8 Hz, 1H), 6.37 (d, *J* = 15.6 Hz, 1H), 6.02 (s, 1H), 3.69 (s, 3H), 3.44 (q, *J* = 6.7 Hz, 2H), 2.42 (t, *J* = 7.0 Hz, 2H), 1.92 (p, *J* = 6.9 Hz, 2H). ^{13}C – NMR (125 MHz, $CDCl_3$): δ 174.1, 165.5, 139.5, 137.0, 132.5, 130.4, 130.3, 126.6, 123.0, 122.0, 51.9, 39.4, 31.7, 24.6. HRMS (ESI) *m/z*: $[M+H]^+$ Calculated for $C_{14}H_{17}BrNO_3$ 326.0386

16b

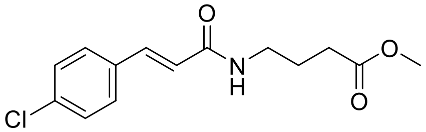

Found 326.0379.

methyl (E) - 4- (3- (4- chlorophenyl) acrylamido) butanoate (16b): Starting from (E)-3-(4-chlorophenyl)acrylic acid (16). White solid. Yield: 52%. m.p.: 102-103°C (10% *v/v* MeOH/DCM). IR (KBr, cm^{-1}): 3312.0, 1655.2. ^{1}H – NMR (400 MHz, $CDCl_3$): δ 7.58 (d, *J* = 15.6 Hz, 1H), 7.42 (d, *J* = 8.4 Hz, 2H), 7.32 (d, *J* = 8.4 Hz, 2H), 6.62 (brs, 1H), 6.47 (d, *J* = 15.6 Hz, 1H), 3.69 (s, 3H), 3.46 (dd, *J* = 12.7, 6.6 Hz, 2H), 2.45 (t, *J* = 7.2 Hz, 2H), 1.95 (p, *J* = 7.0 Hz, 2H). ^{13}C – NMR (100 MHz, $CDCl_3$): δ 174.0, 166.0, 140.0, 136.0, 133.4, 129.1, 129.0, 121.5, 51.8, 39.3, 31.6, 24.7. HRMS (ESI) *m/z:* $[M+H]^+$ Calculated for $C_{14}H_{17}ClNO_3$ 282.0897 Found 282.0900

17b

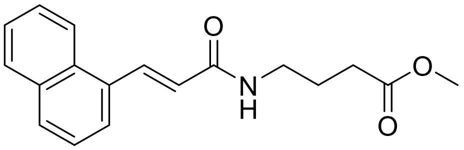

methyl (E)-4-(3-(naphthalen-1-yl) acrylamido) butanoate (17b): Starting from (E)-3-(naphthalen-1-yl)acrylic acid (17). White solid. Yield: 56%. m.p.: 115-117°C (PS). ^{1}H – NMR (500 MHz, $CDCl_3$): δ 8.44 (d, *J* = 15.4 Hz, 1H), 8.20 (d, *J* = 8.0 Hz, 1H), 7.86 – 7.80 (m, 2H), 7.66 (d, *J* = 7.1 Hz, 1H), 7.54 – 7.47 (m, 2H), 7.41 (t, J = 7.7 Hz, 1H), 6.47 (d, *J* = 15.4 Hz, 1H), 6.30 (s, 1H), 3.67 (s, 3H), 3.47 (dd, *J* = 12.8, 6.6 Hz, 2H), 2.43 (t, *J* = 7.1 Hz, 2H), 1.94 (p, *J* = 7.0 Hz, 2H). ^{13}C – NMR (125 MHz, $CDCl_3$): δ 174.2, 166.1, 138.2, 133.7, 132.5, 131.6, 129.9, 128.7, 126.8, 126.3, 125.5, 124.6, 123.7, 123.6, 51.8, 39.4, 31.7, 24.7. HRMS (ESI) *m*/*z*: $[M+H]^+$ Calculated for $C_{18}H_{20}NO_3$ 298.1438 Found 298.1437.

18b

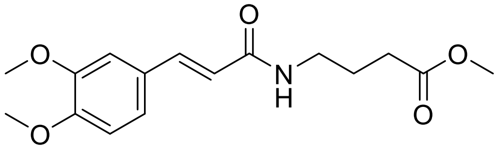

methyl (E)-4-(3-(3,4-dimethoxyphenyl) acrylamido) butanoate (18b): Starting from (E)-3-(3,4-dimethoxyphenyl) acrylic acid (18). White solid. Yield: 83%. m.p.: 100-102°C (PS). IR (KBr,cm^{-1}): 3294.5, 1737.0, 1651.5. ^{1}H – NMR (500 MHz, $CDCl_3$): δ 7.49 (d, *J* = 15.6 Hz, 1H), 6.99 (dd, *J* = 8.3, 1.4 Hz, 1H), 6.93 (d, *J* = 1.5 Hz, 1H), 6.74 (d, *J* = 8.3 Hz, 1H), 6.49 (t, *J* = 5.1 Hz, 1H), 6.31 (d, *J* = 15.6 Hz, 1H), 3.81 (s, 3H), 3.79 (s, 3H), 3.60 (s, 3H), 3.37 (q, *J* = 6.5 Hz, 2H), 2.35 (t, *J* = 7.2 Hz, 2H), 1.85 (p, *J* = 7.0 Hz, 2H). ^{13}C – NMR (125 MHz, $CDCl_3$): δ 173.9, 166.5, 150.4, 149.0, 140.5, 127.8, 121.7, 118.8, 111.1, 109.8, 55.8, 51.7, 39.1, 31.5, 24.7. HRMS (ESI) *m*/*z*: $[M+H]^+$ Calculated for $C_{16}H_{22}NO_5$ 308.1492 Found 308.1499.

19b

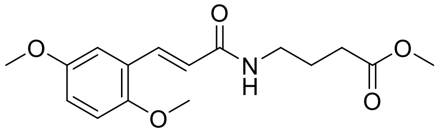

methyl (E)-4-(3-(2,5-dimethoxyphenyl) acrylamido) butanoate (19b): Starting from (E)-3-(2,5-dimethoxyphenyl) acrylic acid (19). Light yellow oil. Yield: 62%. IR (KBr, cm^{-1}): 3288.5, 1734.2, 1647.5. ^{1}H – NMR (500 MHz, $CDCl_3$): δ 7.80 (d, *J* = 15.8 Hz, 1H), 6.97 (d, *J* = 2.8 Hz, 1H), 6.83 (dd, *J* = 9.0, 2.9 Hz, 1H), 6.79 (d, *J* = 9.0 Hz, 1H), 6.50 (d, *J* = 15.8 Hz, 1H), 6.29 (brs, 1H), 3.77 (s, 3H), 3.73 (s, 3H), 3.64 (s, 3H), 3.40 (dd, *J* = 12.8, 6.7 Hz, 2H), 2.38 (t, *J* = 7.2 Hz, 2H), 1.88 (p, *J* = 7.0 Hz, 2H). ^{13}C – NMR (125 MHz, $CDCl_3$): δ 174.0, 166.7, 153.5, 152.7, 136.0, 124.5, 122.0, 116.0, 113.7, 112.40, 56.1, 55.8, 51.8, 39.2, 31.6, 24.8. HRMS (ESI) *m*/*z*: $[M+H]^+$ Calculated for $C_{16}H_{22}NO_5$ 308.1492 Found 308.1494.

20b

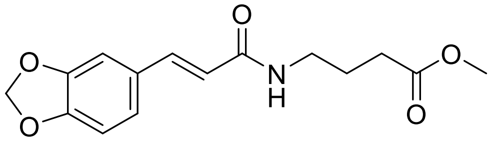

methyl (E) – 4 - (3 - (benzo[d][1,3]dioxol-5-yl) acrylamido) butanoate (20b): Starting from (E)-3-(benzo[d][1,3]dioxol-5-yl) acrylic acid (20). Off-white solid. Yield: 59%. m.p.: 70-72°C (1PS:2EA). IR (KBr, cm^{-1}): 3295.2, 1735.5, 1722.7. ^{1}H – NMR (250 MHz, $CDCl_3$): δ 7.51 (d, *J* = 15.5 Hz, 1H), 6.97 (d, *J* = 8.1 Hz, 2H), 6.78 (d, *J* = 7.8 Hz, 1H), 6.21 (d, *J* = 15.5 Hz, 1H), 5.98 (s, 2H), 3.67 (s, 3H), 3.42 (q, *J* = 6.6 Hz, 2H), 2.41 (t, *J* = 7.1 Hz, 2H), 1.90 (p, *J* = 6.9 Hz, 2H). ^{13}C – NMR (63 MHz, $CDCl_3$): δ 174.2, 166.3, 149.2, 148.4, 140.8, 129.4, 123.9, 118.8, 108.7, 106.5, 101.6, 51.9, 39.3, 31.7, 24.8. HRMS (ESI) *m*/*z*: $[M+H]^+$ Calculated for $C_{15}H_{18}NO_5$ 291.1180 Found 291.1189.

21b

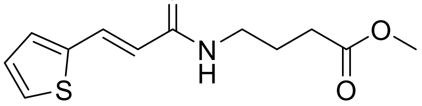

methyl (E) – 4 - (3-(thiophen-2-yl) acrylamido) butanoate (21b): Starting from (E)-3-(thiophen-2-yl)acrylic acid (21). Off-white solid. Yield: 79%. m.p.: 63-65°C (1PS:2EA). IR (KBr, cm^{-1}) : 3255.5, 1731.4, 1648.3. ^{1}H - NMR (500 MHz, $CDCl_3$): δ 7.70 (d, *J* = 15.3 Hz, 1H), 7.26 (d, *J* = 4.9 Hz, 1H), 7.16 (d, *J* = 3.1 Hz, 1H), 6.99 (t, *J* = 4.1 Hz, 1H), 6.30 (s, 1H), 6.22 (d, *J* = 15.3 Hz, 1H), 3.65 (s, 3H), 3.40 (dd, *J* = 12.7, 6.3 Hz, 2H), 2.39 (t, *J* = 7.1 Hz, 2H), 1.88 (p, *J* = 6.9 Hz, 2H). ^{13}C - NMR (125 MHz, $CDCl_3$): δ 174.0, 166.0, 140.1, 133.8, 130.4, 128.1, 127.4, 119.8, 51.9, 39.2, 31.6, 24.8. HRMS (ESI) *m/z*: $[M+H]^+$ Calculated for $C_{12}H_{16}NO_3S$ 254.0851 Found 254.0848

22b

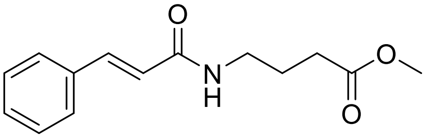

methyl 4-cinnamamidobutanoate (22b): Starting from trans-cinnamic acid (22). White solid. Yield: 71%. m.p.: 70-72°C (PS). IR (KBr, cm^{-1}): 3282.0, 1653.1. ^{1}H – NMR (500 MHz, $CDCl_3$): δ 7.62 (d, *J* = 15.6 Hz, 1H), 7.50 (dd, *J* = 7.5, 1.8 Hz, 2H), 7.37 (m, 3H), 6.38 (d, *J* = 15.6 Hz, 1H), 5.92 (s, 1H), 3.68 (s, 3H), 3.44 (dd, *J* = 12.8, 6.7 Hz, 2H), 2.43 (t, *J* = 7.1 Hz, 2H), 1.92 (p, *J* = 6.9 Hz, 2H). ^{13}C – NMR (125 MHz, $CDCl_3$): δ 174.2, 166.1, 141.2, 135.0, 129.9, 129.0, 127.9, 120.7, 51.9, 39.4, 31.8, 24.9. HRMS (ESI) *m/z*: $[M+H]^+$ Calculated for

23b

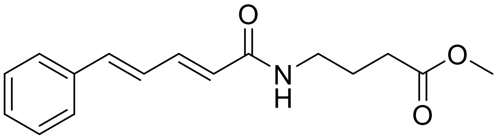

$C_{14}H_{18}NO_3$ 248.1286 Found 248.1285.

methyl 4 - ((2E,4E)-5-phenylpenta-2,4-dienamido) butanoate (23b): Starting from (2E,4E)-5-phenylpenta-2,4-dienoic acid (23). White solid. Yield: 66%. m.p.: 115-117°C (2PS:1EA). IR (KBr, cm^{-1}): 3270.7, 1728.3, 1645.9. ^{1}H – NMR (500 MHz, $CDCl_3$): δ 7.44 (d, *J* = 7.3 Hz, 2H), 7.40 – 7.36 (m, 1H), 7.34 (t, *J* = 7.4 Hz, 2H), 7.29 (d, *J* = 7.2 Hz, 1H), 6.85 (s, 1H), 6.84 (d, *J*=5.5 Hz, 1H), 5.95 (d, *J* = 14.9 Hz, 1H), 5.89 (brs, 1H), 3.68 (s, 3H), 3.41 (q, *J* = 6.7 Hz, 2H), 2.41 (t, *J* = 7.1 Hz, 2H), 1.90 (p, *J* = 6.9 Hz, 2H). ^{13}C – NMR (125 MHz, $CDCl_3$): δ 192.3, 174.2, 141.2, 139.4, 136.4, 128.9, 128.9, 127.1, 126.4, 123.9, 51.9, 39.3, 31.7, 24.8. HRMS *m/z*: $[M+H]^+$

24b

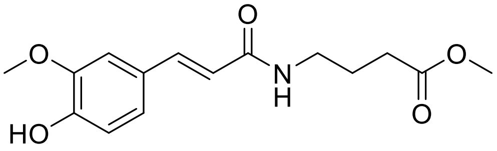

Calculated for $C_{16}H_{20}NO_3$ 274.1438 Found 274.1440.

methyl (E) – 4 - (3 - (4 – hydroxy – 3 - methoxyphenyl) acrylamido) butanoate (24b): [52] Starting from trans-ferulic acid (24). Oil. Yield: 65%. IR (KBr, cm^{-1}): 3596.6, 1742.6, 1706.2. ^{1}H – NMR (500 MHz, Acetone-d_6): δ 7.44 (d, *J* = 15.6 Hz, 1H), 7.28 (brs, 1H), 7.16 (d, *J* = 1.5 Hz, 1H), 7.04 (dd, *J* = 8.1, 1.7 Hz, 1H), 6.84 (d, *J* = 8.2 Hz, 1H), 6.49 (d, *J* = 15.6 Hz, 1H), 3.88 (s, 3H), 3.61 (s, 3H), 3.34 (dd, *J* = 12.5, 6.5 Hz, 2H), 2.38 (t, *J* = 7.4 Hz, 2H), 2.09 (s, 1H), 1.83 (p, *J* = 7.2 Hz, 2H). HRMS (ESI) *m/z*: $[M+H]^+$ Calculated for $C_{15}H_{19}NO_5$ 294.1336 Found 294.1340.

methyl (E) – 4 - (3 - (4-hydroxyphenyl) acrylamido) butanoate (25b): Starting from trans-coumaric acid

25b

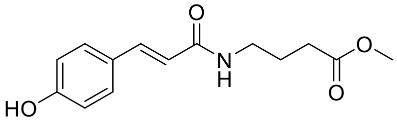

(25). Yellow solid. Yield: 59%. m.p.: 155-157°C (1PS:2EA). IR (KBr, cm^{-1}): 3375.2, 1732.6, 1654.9. ^{1}H – NMR (250 MHz, DMSO-d_6): δ 9.82 (s, 1H), 8.00 (t, J = 5.5 Hz, 1H), 7.38 (d, *J* = 8.5 Hz, 2H), 7.30 (d, *J* = 15.7 Hz, 1H), 6.78 (d, *J* = 8.5 Hz, 2H), 6.37 (d, *J* = 15.7 Hz, 1H), 3.59 (s, 3H), 3.16 (q, *J* = 6.6 Hz, 2H), 2.34 (t, *J* = 7.4 Hz, 2H), 1.70 (p, *J* = 7.1 Hz, 2H). ^{13}C-NMR (63 MHz, DMSO-d_6): δ 173.1, 165.4, 158.8, 138.6, 129.1, 125.9, 118.6, 115.7,

26b

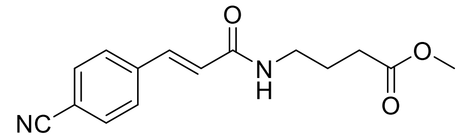

51.2, 37.9, 30.8, 24.6. HRMS (ESI) *m/z*: [M+H]$^+$ Calculated for $C_{14}H_{18}NO_4$ 264.1230 Found 264.1232.

methyl (E) – 4 - (3 - (4-cyanophenyl) acrylamido) butanoate (26b): Starting from (E)-3-(4-cyanophenyl)acrylic acid (26). White solid. Yield: 58%. m.p.: 123-125°C (PS). IR (KBr, cm^{-1}): 3267.9, 2230.0, 1732.1, 1651.5, 1607.9. ^{1}H – NMR (500 MHz, DMSO-d_6): δ 8.27 (t, *J* = 5.6 Hz, 1H), 7.87 (d, *J* = 8.3 Hz, 2H), 7.75 (d, *J* = 8.4 Hz, 2H), 7.47 (d, *J* = 15.8 Hz, 1H), 6.74 (d, *J* = 15.9 Hz, 1H), 3.59 (s 3H), 3.20 (q, *J* = 6.6 Hz, 2H)), 2.35 (t, *J* = 7.4 Hz, 2H), 1.72 (p, *J* = 7.2 Hz, 2H). ^{13}C – NMR (125 MHz, DMSO-d_6): δ 173.0, 164.4, 139.6, 136.8, 132.8, 128.2, 125.7, 118.7, 111.3, 51.3, 38.1, 30.7, 24.5. HRMS (ESI) *m/z:* [M+H]$^+$ Calculated for $C_{15}H_{17}N_2O_3$ 273.1239 Found 273.1233.

methyl (E) – 4 - (3 - (4-fluorophenyl) acrylamido) butanoate (27b): Starting from (E)-3-(4-fluorophenyl)acrylic acid (27). White solid, Yield: 63%. m.p.: 111-113°C (1PS:1EA). IR (KBr, cm^{-1}): 3253.3,

28b

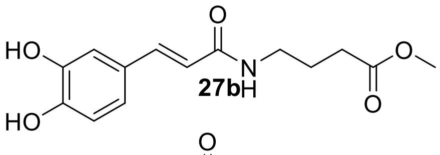

27b

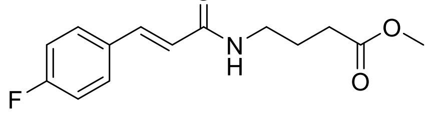

1736.3, 1657.9. ^{1}H – NMR (500 MHz, $CDCl_3$): δ 7.57 (d, *J* = 15.6 Hz, 1H), 7.46 (dd, *J* = 8.4, 5.5 Hz, 2H), 7.03 (t, *J* = 8.6 Hz, 2H), 6.31 (d, *J* = 15.6 Hz, 1H), 6.09 (brs, 1H), 3.67 (s, 3H), 3.43 (q, *J* = 6.5 Hz, 2H), 2.41 (t, *J* = 7.1 Hz, 2H), 1.91 (p, *J* = 7.1 Hz, 2H). ^{13}C – NMR (125 MHz, $CDCl_3$): δ 174.0, 166.0, 163.6 (d, *J*=248.8 Hz), 147.2, 139.9, 131.2 (d, *J*=3.8 Hz), 129.7 (d, *J*=7.5Hz), 120.5 (d, *J*=1.25 Hz), 116.0 (d, *J*=22.5 Hz), 51.9, 39.4, 31.7, 24.7. HRMS (ESI) *m/z:* [M+H]$^+$ Calculated for $C_{14}H_{17}FNO_3$ 266.1187 Found 266.1180.

methyl (E) – 4 - (3 - (3,4-dihydroxyphenyl) acrylamido) butanoate (28b): Starting from trans-caffeic acid (28). White solid. Yield: 35%. m.p.: 158-160°C (Et_2O). IR (KBr, cm^{-1}): 3460.2, 1740.7, 1663.4. ^{1}H – NMR (500 MHz, Acetone-d_6): δ 8.39 (s, 1H), 8.21 (s, 1H), 7.39 (d, *J* = 15.6 Hz, 1H), 7.25 (s, 1H), 7.06 (d, *J* = 1.8 Hz, 1H), 6.92 (dd, J_1 = 8.1 Hz, J_2 = 1.8 Hz, 1H), 6.83 (d, *J* = 8.1 Hz, 1H), 6.41 (d, *J* = 15.6 Hz, 1), 3.61 (s, 3H), 3.33 (dd, J_1 = 12.9 Hz, J_2 = 6.6 Hz, 2H), 2.37 (t, *J* = 7.5 Hz, 2H), 1.85 – 1.79 (p, *J* = 7.2 Hz, 2H). ^{13}C – NMR (125 MHz, Acetone-d_6) δ 173.9, 186.5, 147.8, 146.3, 140.5, 128.5, 121.5, 119.7, 116.4, 114.9, 51.6, 39.2, 31.8, 25.9. HRMS (ESI) *m/z*: [M+H]$^+$ Calculated for $C_{14}H_{18}NO_5$ 280.1180 Found 280.1182.

29b

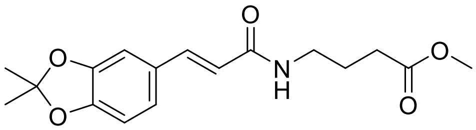

methyl (E)-4-(3-(2,2-dimethylbenzo[d][1,3]dioxol-5-yl)acrylamido)butanoate (29b): Starting from (E)-3-(2,2-dimethylbenzo[d][1,3]dioxol-5-yl)acrylic acid (29). Oil. Yield: 63%. IR (KBr, cm^{-1}): 3291.3, 1734.4, 1632.4. ^1H – NMR (500 MHz, $CDCl_3$): δ 7.47 (d, *J* = 15.5 Hz, 1H), 6.90 – 6.85 (m, 2H), 6.64 (d, *J* = 7.8 Hz, 1H), 6.37 (t, *J* = 5.3 Hz, 1H), 6.23 (d, *J* = 15.5 Hz, 1H), 3.63 (s, 3H), 3.41 – 3.36 (m, 2H), 2.38 (t, *J*=7.2 Hz, 2H), 1.87 (p, *J* = 7.0 Hz, 2H), 1.63 (s, 6H). ^{13}C – NMR (125 MHz, $CDCl_3$): δ 174.1, 166.6, 148.9, 148.0, 140.1, 128.7, 123.4, 118.7, 118.3, 108.4, 106.2, 51.8, 39.2, 31.6, 25.9, 24.8. HRMS (ESI) *m/z:* $[M+H]^+$ Calculated for $C_{17}H_{22}NO_5$ 320.1493 Found 320.1496**.**

30b

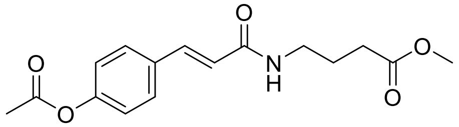

methyl (E) – 4 - (3-(4-acetoxyphenyl) acrylamido) butanoate (30b): Starting from (E)-3-(4-acetoxyphenyl)acrylic acid (30). White solid. Yield: 66%. m.p.: 100-102°C (PS). IR (KBr, cm^{-1}): 3286.6, 1763.2, 1733.0, 1652.2. ^1H – NMR (500 MHz, $CDCl_3$): δ 7.58 (d, *J* = 15.6 Hz, 1H), 7.49 (d, *J* = 8.2 Hz, 2H), 7.08 (d, *J* = 8.2 Hz, 2H), 6.33 (d, *J* = 15.6 Hz, 1H), 6.10 (brs, 1H), 3.67 (s, 3H), 3.42 (d, *J* = 6.0 Hz, 2H), 2.40 (t, *J* = 7.0 Hz, 2H), 2.30 (s, 3H), 1.90 (p, *J* = 7.0 Hz, 1H). ^{13}C – NMR (125 MHz, $CDCl_3$): δ 174.2, 169.4, 166.0, 151.7, 140.0, 132.7, 129.0, 122.1, 120.9, 51.9, 39.4, 31.7, 24.7, 21.3. HRMS (ESI) *m/z*: $[M+H]^+$ Calculated for $C_{16}H_{20}NO_5$ 306.1341 Found 306.1339

11c

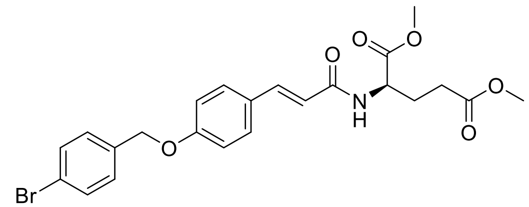

dimethyl (E) - (3 - (4-((4-bromobenzyl) oxy) phenyl) acryloyl)-L-glutamate (11c): Starting from (E)-3-(4-((4-bromobenzyl)oxy)phenyl) acrylic acid (11). White solid. Yield: 84%. m.p.: 120-122°C (1PS:1EA). IR (KBr, cm^{-1}): 3274.0, 1739.2, 1653.8, 1623.2. ^1H – NMR (500 MHz, $CDCl_3$): δ 7.58 (d, *J* = 15.6 Hz, 1H), 7.52 (d, *J* = 8.3 Hz, 2H), 7.45 (d, *J* = 8.7 Hz, 2H), 7.30 (d, *J* = 8.2 Hz, 2H), 6.94 (d, *J* = 8.6 Hz, 2H), 6.33 (d, *J*=5.0 Hz, 1H), 6.31 (d, *J*=15.0 Hz, 1H), 5.04 (s, 2H), 4.77 (td, J_1 = 7.7 Hz, J_2 = 5.2 Hz, 1H), 3.77 (s, 3H), 3.67 (s, 3H), 2.51-2.41 (m, 2H), 2.32-2.25 (m, 1H), 2.11-2.05 (m, 1H). ^{13}C – NMR (125 MHz, $CDCl_3$): δ 173.4, 172.5, 165.9, 159.9, 141.4, 135.5, 131.8, 129.5, 129.0, 127.8, 122.0, 117.8, 115.1, 69.3, 53.0, 51.9, 51.8, 30.1, 27.5. HRMS (ESI) *m/z:* $[M+H]^+$ Calculated for $C_{23}H_{25}BrNO_6$ 490.0860 / 492.0843 Found 490.0859 / 492.0842 (1:1).

12c

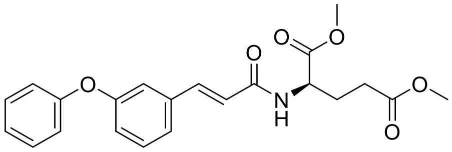

dimethyl (E) - (3 - (3-phenoxyphenyl) acryloyl) - L-glutamate (12c) [53]: Starting from (E)-3-(3-phenoxyohenyl)acrylic acid (12). Oil. Yield: 88%. IR (KBr, cm^{-1}): 3320.9, 1724.1, 1665.4. ^1H – NMR (500 MHz, $CDCl_3$): δ 7.57 (d, J = 15.6 Hz, 1H), 7.37 – 7.29 (m, 3H), 7.22 (d, *J* = 7.5 Hz, 1H), 7.13 (d, *J* = 9.8 Hz, 2H), 7.03 – 6.97 (m, 2H), 6.46 (d, *J* = 7.4 Hz, 1H), 6.38 (d, *J* = 15.6 Hz, 1H), 4.76 (td, *J* = 5.1, 2.1 Hz, 1H), 3.76 (s, 3H), 3.66 (s, 3H), 2.50-2.36 (m, 2H), 2.30-2.23 (m, 1H), 2.09-2.02 (m, 1H). HRMS (ESI) *m/z:* $[M+Na]^+$ Calculated for $C_{22}H_{23}NO_6Na$ 420.1418 Found 420.1410.

13c

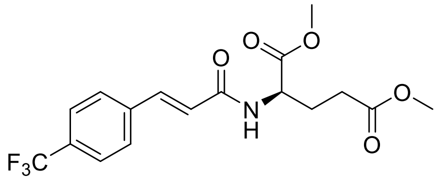

(-) - dimethyl (E) - (3 - (4 - (trifluoromethyl) phenyl) acryloyl)-L-glutamate (13c): Starting from (E)-3-(4-(trifluoromethyl)phenyl)acrylic acid (13). Off-white solid. Yield: 51%. $[\alpha]_{589}^{25}$= -6° (10^{-1} g/mL in MeOH). IR (KBr, cm^{-1}): 3321.4, 1720.5, 1661.6, 1631.4. ^{1}H-NMR (500 MHz, $CDCl_3$) δ 7.64 (d, *J* = 15.0 Hz, 1H), 7.60 (dd, *J* = 6.3 Hz, 4H), 6.59 (d, *J* = 7.6 Hz, 1H), 6.52 (d, *J* = 15.7 Hz, 1H), 4.77 (td, J_1 = 7.8 Hz J_2 = 5.1 Hz, 1H), 3.78 (s, 3H), 3.68 (s, 3H), 2.52-2.39 (m, 2H), 2.33-2.26 (m, 1H), 2.13-2.06 (m, 1H). ^{13}C-NMR (125 MHz, $CDCl_3$) δ 173.6, 172.5, 166.2, 140.3, 138.1 (d, *J*=1.25 Hz), 131.5 (d, *J*=32.5 Hz), 128.1, 126.3 (q, *J*=271.4 Hz), 125.8 (q, *J*=3.7 Hz), 122.5, 52.8, 52.1, 52.0, 30.2, 27.5. HRMS (ESI) *m/z:* $[M+H]^+$ Calculated for $C_{17}H_{18}F_3NO_5$ 374.1210 Found 374.1212.

14c

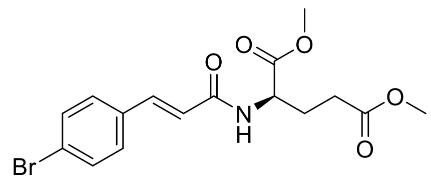

(+) - dimethyl (E)-(3-(4-bromophenyl)acryloyl)-L-glutamate (14c): Starting from (E)-3-(4-bromophenyl) acrylic acid (14). Off-white solid. Yield: 85%. m.p.: 145-147°C. $[\alpha]_{589}^{25}$= +30° (10^{-2} g/mL in MeOH). IR (KBr, cm^{-1}): 3280.1, 1726.1, 1748.8, 1654.0. ^{1}H – NMR (500 MHz, $CDCl_3$): δ 7.57 (d, *J* = 15.6 Hz, 1H), 7.51 (d, *J* = 8.4 Hz, 2H), 7.37 (d, J = 8.5 Hz, 2H), 6.82 (d, J = 8.3 Hz, 1H), 6.42 (d, *J*=15.6 Hz, 1H), 4.77 (td, J = 7.7, 5.1 Hz, 1H), 3.78 (s, 3H), 3.68 (s, 3H), 2.52 – 2.38 (m, 2H), 2.29 (m, 1H), 2.09 (m, 1H). ^{13}C – NMR (125 MHz, $CDCl_3$): δ 174.0, 172.5, 165.5, 140.8, 133.7, 132.2, 129.4, 124.2, 120.7, 52.8, 52.1, 52.0, 30.2, 27.5. HRMS (ESI) m/z: $[M+H]^+$

15c

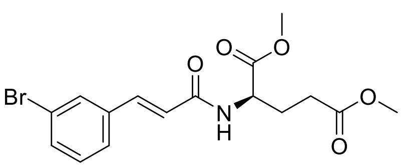

Calculated for $C_{16}H_{19}BrNO_5$ 384.0441/386.0415 Found 384.0443/386.0411 (1:1).

(-) - dimethyl (E)-(3-(3-bromophenyl)acryloyl)-L-glutamate (15c): Starting from (E)-3-(3-bromophenyl) acrylic acid (15). White solid. Yield: 88%. $[\alpha]_{589}^{25}$. = -12° (10^{-2} g/mL in MeOH). IR (KBr, cm^{-1}): 3310.6, 1745.7, 1733.1, 1654.6. ^{1}H-NMR (500 MHz, $CDCl_3$): δ 7.68 (s, 1H), 7.59 (d, *J* = 15.6 Hz, 1H), 7.51 (d, *J* = 7.9 Hz, 1H), 7.44 (d, *J* = 7.7 Hz, 1H), 7.28 (d, *J* = 8.6 Hz, 1H), 6.53 (d, *J* = 7.5 Hz, 1H), 6.47 (d, *J* = 15.6 Hz, 1H), 4.80 (td, J_1 = 7.6Hz, J_2= 5.3 Hz, 1H), 3.81 (s, 3H), 3.71 (s, 3H), 2.56 – 2.41 (m, 2H), 2.36 – 2.28 (m, 1H), 2.12 (m, 1H). ^{13}C - NMR (125 MHz, $CDCl_3$): δ 173.5, 172.5, 165.3, 140.4, 136.9, 132.8, 131.0, 130.5, 126.7, 123.1, 121.5, 52.8, 52.0, 30.2, 27.5. HRMS (ESI) *m/z*: $[M+H]^+$ Calculated for $C_{16}H_{19}BrNO_5$ 384.0441/386.0415 Found 384.0437/386.0411.

16c

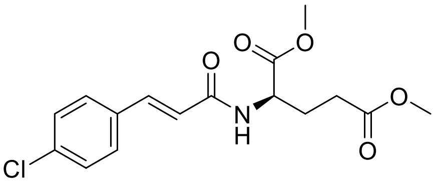

(-)-dimethyl (E) - (3 - (4-chlorophenyl)acryloyl)-L-glutamate (16c): Starting from (E)-3-(4-chlorophenyl) acrylic acid (16). White solid. Yield: 89%. m.p.: 157-158°C (1PS:1EA). $[\alpha]_{589}^{25}$= -16° (10^{-2} g/mL in MeOH). IR

17c

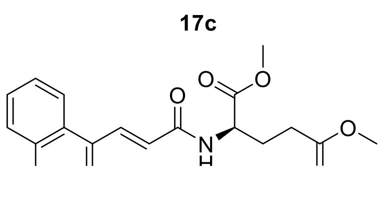

(KBr, cm^{-1}): 3305.8, 1748.9, 1724.7, 1655.7. ^{1}H – NMR (500 MHz, $CDCl_3$): δ 7.58 (d, *J* = 15.6 Hz, 1H), 7.43 (d, *J* = 8.5 Hz, 2H), 7.35 (d, *J* = 8.5 Hz, 2H), 6.40 (d, *J*=15.6 Hz, 1H), 6.42 (d, *J* = 5.2 Hz, 1H), 4.77 (td, *J* = 7.7, 5.1 Hz, 1H), 3.78 (s, 3H), 3.68 (s, 3H), 2.52 – 2.37 (m, 2H), 2.32 – 2.25 (m, 1H), 2.11-2.04 (m, 1H). ^{13}C – NMR (125 MHz, $CDCl_3$): δ 173.2, 171.8, 167.8, 160.7, 153.7, 149.3, 143.0, 120.0, 119.4, 67.9, 52.8, 52.0, 30.1, 27.0. HRMS (ESI) *m/z*: $[M+H]^+$ Calculated for $C_{16}H_{19}ClNO_5$ 340.0946 / 342.0940 Found 342.0936 / 342.0936 (3:1).

(-) - dimethyl (E)-(3-(naphthalen-1-yl)acryloyl)-L-glutamate (17c): Starting from (E)-3-(naphthalen-1-yl)acrylic acid (17). White solid. Yield: 44%. $[\alpha]^{25}_{589}$= -28° (10^{-2} g/mL in MeOH). m.p.: 149-150°C (1PS:2EA). IR (KBr, cm^{-1}): 3303.7, 1748.5, 1727.0, 1652.8. ^{1}H-NMR (500 MHz, $CDCl_3$) δ 8.47 (d, *J* = 15.4 Hz, 1H), 8.20 (d, *J* = 8.1 Hz, 1H), 7.86 (dd, *J* = 7.8, 1.9 Hz, 2H), 7.70 (d, *J* = 7.1 Hz, 1H), 7.56 – 7.48 (m, 2H), 7.45 (t, *J* = 7.7 Hz, 1H), 6.64 (d, *J* = 7.3 Hz, 1H), 6.53 (d, *J* = 15.4 Hz, 1H), 4.82 (dd, *J* = 12.8, 7.7 Hz, 1H), 3.78 (s, 3H), 3.68 (s, 3H), 2.55-2.42 (m, 2H), 2.35-2.28 (m, 1H), 2.14-2.08 (m, 1H). ^{13}C-NMR (125 MHz, $CDCl_3$) δ: 173.5, 172.6, 165.8, 139.1, 133.7, 132.2, 131.5, 130.1, 128.7, 126.8, 126.2, 125.4, 124.7, 123.6, 122.8, 52.7, 52.0, 30.3, 27.5. HRMS (ESI) *m/z:* $[M+H]^+$ Calculated for $C_{20}H_{22}NO_5$ 356.1493 Found 356.1487.

18c

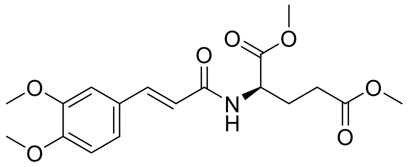

(-) - dimethyl (E) - (3 - (3,4 - dimethoxyphenyl) acryloyl)-L-glutamate (18c): Starting from (E)-3-(3,4-dimethoxyphenyl) acrylic acid (18). Yellow oil. Yield: 99%. $[\alpha]^{25}_{589}$= -15° (8×10^{-3} g/mL in MeOH). IR (KBr, cm^{-1}): 3361.3, 1759.4, 1681.1. ^{1}H – NMR (500 MHz, $CDCl_3$): δ 7.45 (d, *J* = 15.6 Hz, 1H), 6.93 (d, *J* = 8.1 Hz, 2H), 6.89 (s, 1H), 6.70 (d, *J* = 8.3 Hz, 1H), 6.33 (d, *J* = 15.6 Hz, 1H), 4.68 (td, *J* = 8.0, 5.4 Hz, 1H), 3.77 (s, 3H), 3.75 (s, 3H), 3.64 (s, 3H), 3.55 (s, 3H), 242-2.31 (m, 2H), 2.21-2.14 (m, 1H), 2.00-1.93 (m, 1H). ^{13}C - NMR (125 MHz, $CDCl_3$): δ 173.1, 172.5, 166.1, 150.4, 148.9, 141.4, 127.5, 121.8, 117.8, 110.9, 109.7, 55.7, 52.4, 51.6, 30.0, 27.1. HRMS (ESI) *m/z:* $[M+Na]^+$ Calculated for $C_{18}H_{23}NO_7Na$ 388.1367 Found 388.1360.

19c

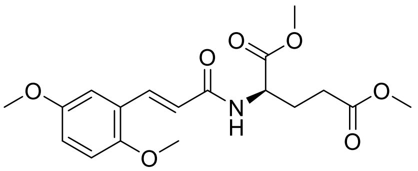

(-) - dimethyl (E) - (3 - (2,5-dimethoxyphenyl) acryloyl)-L-glutamate (19c): Starting from (E)-3-(2,5-dimethoxyphenyl) acrylic acid (19). Semi-solid. Yield: 55% m.p.: 65-67°C (2PS:1EA). $[\alpha]^{25}_{589}$= -16° (10^{-2} g/mL in MeOH). IR (KBr, cm^{-1}): 3264.7, 1744.6, 1672.1. ^{1}H – NMR (500 MHz, $CDCl_3$): δ 7.83 (d, *J* = 15.8 Hz, 1H), 7.00 (d, *J* = 2.9 Hz, 1), 6.87 (dd, *J* = 9.0, 2.9 Hz, 1H), 6.83 (d, *J* = 9.0 Hz, 1H), 6.55 (d, *J* = 15.8 Hz, 1H), 6.40 (d, *J* = 7.7 Hz, 1H), 4.78 (td, J_1 = 7.9Hz, J_2= 5.1 Hz, 1H), 3.82 (s, 3H), 3.77 (s, 3H), 3.76 (s, 3H), 3.66 (s, 3H), 2.51 – 2.37 (m, 2H), 2.30-2.25 (m, 1H), 2.10-2.03 (m, 1H). ^{13}C – NMR (125 MHz, $CDCl_3$): δ 173.5, 172.8, 166.3, 153.6, 153.0, 137.4, 124.3, 121.2, 116.6, 114.0, 112.5, 56.0, 52.7, 52.0, 51.9, 30.2, 27.7. HRMS (ESI) *m/z*: $[M+H]^+$ Calculated for $C_{18}H_{24}NO_7$ 366.1547 Found 366.1546.

20c

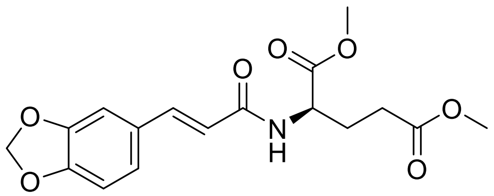

(-) - dimethyl (E) - (3 - (benzo[d][1,3]dioxol-5-yl)acryloyl)-L-glutamate (20c): Starting from (E)-3-(benzo[d][1,3]dioxol-5-yl)acrylic acid (20). Off-white solid. Yield: 45%. m.p.: 87-89°C (1PS:1EA). $[\alpha]^{25}_{589}$= -16.4° (11×10^{-3} g/mL in MeOH). IR (KBr, cm^{-1}): 3273.8, 1744.7, 1654.0, 1610.3. ^{1}H – NMR (250 MHz, $CDCl_3$): δ 7.53 (d, *J* = 15.5 Hz, 1H), 7.00 (s, 1H), 6.96 (d, *J* = 8.1 Hz, 1H), 6.79 (d, *J* = 8.0 Hz, 1H), 6.38 (d, *J* = 7.6 Hz, 1H), 6.26 (d, *J* = 15.5 Hz, 1H), 5.99 (s, 2H), 4.77 (td, J_1 = 7.8 Hz, J_2 = 5.2 Hz, 1H), 3.77 (s, 3H), 3.67 (s, 3H), 2.50-2.40

21c

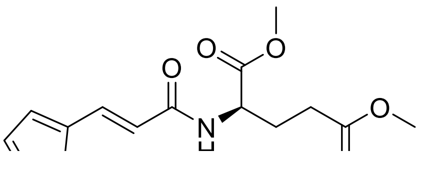

(m, 2H), 2.36-2.21 (m, 1), 2.14-1.99 (m, 1H). ^{13}C – NMR (63 MHz, $CDCl_3$): δ 173.6, 172.6, 166.0, 149.4, 148.4, 141.8, 129.2, 124.2, 118.0, 108.7, 106.6, 52.7, 52.0, 30.3, 27.6. HRMS (ESI) *m/z*: $[M+H]^+$ Calculated for $C_{17}H_{20}NO_7$ 350.1234 Found 350.1235.

(-) - dimethyl (E)-(3-(thiophen-2-yl)acryloyl)-L-glutamate (21c): Starting from 3-(thiophen-2-yl)-acrylic acid (21). Oil. Yield: 79%. $[\alpha]^{25}_{589}$= -25.3° (15×10^{-3} g/mL in MeOH). IR (KBr, cm^{-1}): 3284.4, 1762.2, 1643.2. ^1H – NMR (500 MHz, $CDCl_3$): δ 7.66 (d, *J* = 15.4 Hz, 1H), 7.22 (d, *J* = 5.0 Hz, 1H), 7.09 (d, *J* = 3.3 Hz, 1H), 7.01 (d, *J* = 7.9 Hz, 1H), 6.91 (dd, J_1 = 5.0 Hz, J_2 = 3.7 Hz, 1H), 6.28 (d, *J* = 15.4 Hz, 1H), 4.70 (td, *J* = 8.1, 5.2 Hz, 1H), 3.66 (s, 3H), 3.58 (s, 3H), 2.45 – 2.32 (m, 2), 2.23 – 2.15 (m, 1H), 2.03-1.96 (m, 1H). ^{13}C – NMR (125 MHz, $CDCl_3$): δ 173.2, 172.4, 165.7, 139.7, 134.3, 130.4, 127.9, 127.6, 118.9, 52.5, 51.8, 51.6, 30.1, 27.2. HRMS (ESI) *m/z*: $[M+H]^+$ Calculated for $C_{14}H_{18}NO_5S$ 312.0900 Found 312.0908.

22c

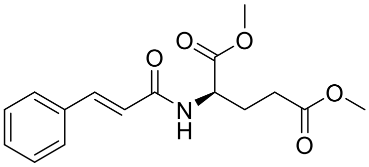

(-)-dimethyl cinnamoyl-L-glutamate (22c): Starting from trans-cinnamic acid (22). White solid. Yield: 68%. m.p.: 105-106°C (1PS:1EA) [68]. $[\alpha]^{25}_{589}$ = -25° ($6.3x10^{-3}$ g/mL in MeOH). IR (KBr, cm^{-1}): 3286.4, 1740.5, 1650.1. ^1H – NMR (400 MHz, $CDCl_3$): δ 7.59 (d, *J* = 15.7 Hz, 1H), 7.47 – 7.40 (m, 2H), 7.32 – 7.27 (m, 3H), 6.87 (d, *J* = 7.8 Hz, 1H), 6.48 (d, *J* = 15.7 Hz, 1H), 4.76 (td, J_1 = 8.0 Hz, J_2 = 5.1 Hz, 1H), 3.71 (s, 3H), 3.62 (s, 3H), 2.51 – 2.35 (m, 2H), 2.30 – 2.20 (m, 1H), 2.09-1.99 (m, 1H). ^{13}C-NMR (100 MHz, $CDCl_3$): δ 173.4, 172.5, 165.9, 141.8, 134.6, 129.8, 128.8, 127.9, 120.0, 68.5, 52.6, 51.8, 30.2, 27.3. HRMS (ESI) *m/z*: $[M+H]^+$ Calculated for $C_{15}H_{20}NO_6$ 306.1341 Found 306.1338.

23c

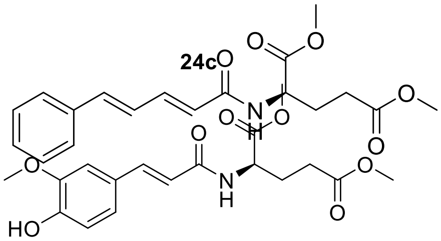

(-) - dimethyl ((2E,4E)-5-phenylpenta-2,4-dienoyl)-L-glutamate (23c): Starting from (2E, 4E)-5-phenylpenta-2,4-dienoic acid (23). White solid. Yield: 78%. m.p.: 122-124°C (2PS:1EA). $[\alpha]^{25}_{589}$= -30° (8×10^{-3} g/mL in MeOH). IR (KBr, cm^{-1}): 3286.3, 1741.3, 1646.5. ^1H – NMR (500 MHz, $CDCl_3$): δ 7.44 (d, *J* = 7.3 Hz, 2H), 7.39 (ddd, J_1 = 14.9 Hz, J_2 = 7.4 Hz, J_3 = 2.7 Hz, 1H), 7.34 (t, *J* = 7.5 Hz, 1H), 7.28 (t, *J* = 7.2 Hz, 1H), 6.86 (s, 1H), 6.85 (d, *J* = 4.8 Hz, 2H), 6.38 (d, *J* = 7.7 Hz, 1H), 6.01 (d, *J* = 14.9 Hz, 1H), 4.75 (td, *J* = 7.8, 5.2 Hz, 1H), 3.76 (s, 3H), 3.67 (s, 3H), 2.50-2.37 (m, 2H), 2.30-2.23 (m, 1H), 2.10-2.02 (m, 1H). ^{13}C – NMR (125 MHz, $CDCl_3$): δ 173.6, 172.6, 166.0, 142.0, 139.9, 136.3, 129.0, 128.9, 127.2, 126.3, 123.2, 52.7, 52.0, 51.9, 30.3, 27.6. HRMS (ESI) *m/z*: $[M+H]^+$ Calculated for $C_{16}H_{22}NO_5$ 332.1493 Found 332.1498.

dimethyl (E) - (3 - (4 - hydroxy-3-methoxyphenyl)acryloyl)-L-glutamate (24c): [54] Starting from trans-ferulic acid (24). Oil. Yield: 58%. IR (KBr, cm^{-1}): 3340.7, 1740.1, 1662.8. ^1H – NMR (500 MHz, $CDCl_3$): δ 7.48 (d, *J* = 15.6 Hz, 1H), 6.95 (d, *J* = 8.2 Hz, 1H), 6.91 (s, 1H), 6.83 (d, *J* = 8.1 Hz, 1H), 6.77 (d, *J* = 7.8 Hz, 1H), 6.30 (d, *J* = 15.6 Hz, 1H), 4.74 (dd, J_1 = 13.0 Hz, J_2 = 7.9 Hz, 1H), 3.82 (s, 3H), 3.71 (s, 3H), 3.62 (s, 3H), 2.49 – 2.35 (m, 2H), 2.29 – 2.20 (m, 1H), 2.07-2.00 (m, 1H), 1.22 (brs, 1H). HRMS (ESI) *m/z:* $[M+Na]^+$ Calculated for $C_{17}H_{21}NO_7Na$ 374.1210 Found 372.1212.

(-) - dimethyl (E)-(3-(4-hydroxyphenyl)acryloyl)-L-glutamate (25c): Starting from trans-coumaric acid (25). White solid. Yield: 60% m.p.: 157-160°C (1PS:2EA). $[\alpha]^{25}_{589}$= -20° (8×10^{-3} g/mL in MeOH). IR (KBr, cm^{-1}): 3433.6, 3310.1, 1737.0, 1689.8. ^1H – NMR (500 MHz, $CDCl_3$): δ 7.52 (d, *J* = 15.6 Hz, 1H), 7.31 (d, *J* = 8.6 Hz, 2H), 6.83 (d, J = 8.6 Hz, 2H), 6.55 (d, *J* = 7.7 Hz, 1H), 6.23 (d, *J* = 15.6 Hz, 1H), 4.76 (td, J_1 = 7.9 Hz, J_2 = 5.0 Hz, 1H), 2.53 – 2.39 (m, 2H), 2.32-2.25 (m, 1H), 2.11-204 (m, 1H), 1.25 (brs, 1H). ^{13}C – NMR (125 MHz, $CDCl_3$): δ 173.8, 172.8, 167.0, 158.5, 142.4, 129.9, 126.7, 116.7, 116.1, 52.9, 52.1, 30.3, 27.4. HRMS (ESI) *m/z*: $[M+H]^+$

25c

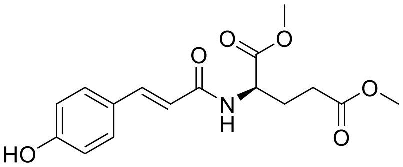

Calculated for $C_{16}H_{20}NO_6$ 322.1286 Found 322.1285.

26c

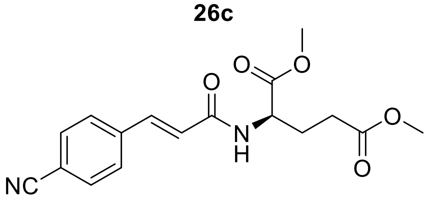

(-) - dimethyl (E)-(3-(4-cyanophenyl)acryloyl)-L-glutamate (26c): Starting from (E)-3-(4-cyanophenyl)acrylic acid (26). White solid. Yield: 69%. m.p.: 99-101°C (1PS:2EA). $[\alpha]_{589}^{25}$= -20° (8×10^{-3} g/mL in MeOH). IR (KBr, cm^{-1}): 3313.9, 1745.4, 1655.7, 1625.9. ^{1}H – NMR (500 MHz, $CDCl_3$): δ 7.65 (d, *J* = 8.3 Hz, 2H), 7.60 (d, *J* = 15.7 Hz, 1H), 7.56 (d, *J* = 8.4 Hz, 2H), 6.70 (d, *J* = 7.6 Hz, 1H), 6.53 (d, *J* = 15.6 Hz, 1H), 4.76 (td, J_1 = 7.8 Hz, J_2 = 5.1 Hz, 1H), 3.77 (s, 3H), 3.67 (s, 3H), 2.51 – 2.37 (m, 2H), 2.30-2.25 (m, 1H), 2.12-2.04 (m, 1H). ^{13}C – NMR (125 MHz, $CDCl_3$): δ 173.5, 172.4, 164.9, 139.7, 139.1, 132.7, 128.4, 123.5, 118.5, 113.1, 52.8, 52.1, 52.0, 30.2, 27.3. HRMS (ESI) *m/z*: [M+H]$^+$ Calculated for $C_{17}H_{19}N_2O_5$ 331.1289 Found 331.1296.

27c

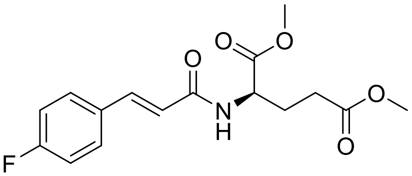

(-) - dimethyl (E)-(3-(4-fluorophenyl)acryloyl)-L-glutamate (27c): Starting from (E)-3-(4-fluorophenyl)acrylic acid (27). White solid. Yield: 44%. m.p.: 145-146°C (1PS:2EA). $[\alpha]_{589}^{25}$= -18.2° (11×10^{-3} g/mL in MeOH). IR (KBr, cm^{-1}): 3306.2, 1748.2, 1728.6, 1636.2. ^{1}H – NMR (500 MHz, $CDCl_3$): δ 7.60 (d, *J* = 15.6 Hz, 1H), 7.49 (dd, J_1 = 8.6 Hz, J_2 = 5.4 Hz, 2H), 7.06 (t, *J* = 8.6 Hz, 2H), 6.42 (d, *J* = 7.4 Hz, 1H), 6.36 (d, *J* = 15.6 Hz, 1H4.77 (td, J_1 = 7.8 Hz, J_2 = 5.1 Hz, 1H), 3.78 (s, 3H), 3.68 (s, 3H), 2.52-2.38 (m, 2H), 2.32-2.25 (m, 1H), 2.12-2.05 (m, 1H). ^{13}C-NMR (125 MHz, $CDCl_3$): δ 173.4, 172.5, 165.7, 163.6 (d, *J*=248.8 Hz), 140.7, 131.0 (d, *J* =3.75 Hz), 129.8 (d, *J* =8.8 Hz), 119.8 (d, *J* =2.5 Hz), 116.0 (d, *J* =21.3 Hz), 52.7, 52.0, 51.9, 30.2, 27.5. HRMS (ESI) *m/z:* [M+H]$^+$ Calculated for $C_{16}H_{19}FNO_5$ 324.1244 Found 324.1244.

28c

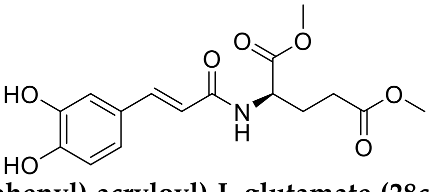

(-) - dimethyl (E) - (3 - (3,4-dihydroxyphenyl) acryloyl)-L-glutamate (28c): Starting from trans-caffeic acid (28). Oil. Yield: 71%. $[\alpha]_{589}^{25}$ = -16° (10^{-2} g/mL in MeOH). IR (KBr, cm^{-1}): 3462.0, 3174.4, 1745.3, 1657.4. ^{1}H – NMR (500 MHz, Acetone-d$_6$): δ 8.37 (s, 2H), 7.60 (d, *J* = 7.9 Hz, 1H), 7.45 (d, *J* = 15.6 Hz, 1H), 7.09 (d, *J* = 1.8 Hz, 1H), 6.95 (dd, J_1 = 8.1 Hz, J_2 = 1.8 Hz, 1H), 6.84 (d, *J* = 8.1 Hz, 1H), 6.55 (d, *J* = 15.6 Hz, 1H), 4.65 (td, J_1 = 8.4 Hz, J_2 = 5.4 Hz, 1H), 3.69 (s, 3H), 3.61 (s, 3H), 2.52 – 2.40 (m, 2H), 2.24 – 2.14 (m, 1H), 2.05-1.97 (m, 1H). ^{13}C – NMR (125 MHz, Acetone-d$_6$): δ 173.4, 173.0, 166.9, 148.1, 146.2, 141.8, 128.1, 121.8, 118.6, 116.3, 114.9, 52.5, 52.4, 51.8, 30.6, 27.8. HRMS (ESI) *m/z:* [M+H]$^+$ Calculated for $C_{16}H_{20}NO_7$ 338.1234 Found 338.1234.

29c

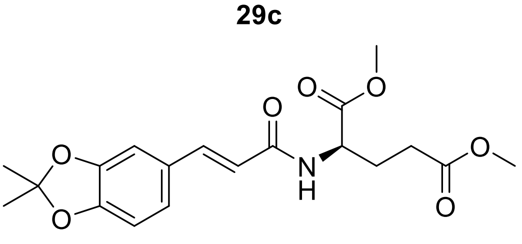

(-) - dimethyl (E) - (3 - (2,2-dimethylbenzo[d][1,3]dioxol-5-yl) acryloyl) – L -glutamate (29c): Starting from (E)-3-(2,2-dimethylbenzo[d][1,3]dioxol-5-yl)acrylic acid (29). Oil. Yield: 64%. $[\alpha]^{25}_{589}$ = -10° (6×10^{-3} g/mL in MeOH). IR (KBr, cm^{-1}): 3276.5, 1740.6, 1657.3. ^{1}H – NMR (500 MHz, $CDCl_3$): δ 7.49 (d, *J* = 15.5 Hz, 1H), 6.89 (m, 2H), 6.65 (d, *J* = 8.0 Hz, 1H), 6.70 (d, *J*=15.5 Hz, 1H), 6.62 (brs, 1H), 4.74 (td, *J* = 8.0, 5.1 Hz, 1H), 3.72 (s, 3H), 3.63 (s, 3H), 2.48 – 2.35 (m, 2H), 22.27-2.20 (m, 1H), 2.07-2.00 (m, 1H), 1.64 (s, 6H). ^{13}C – NMR (125 MHz, $CDCl_3$): δ 179.5, 172.6, 166.2, 149.2, 148.1, 141.9, 128.9, 123.7, 118.8, 117.4, 108.4, 106.3, 52.6, 51.9, 51.8, 30.2, 27.5, 25.9. HRMS (ESI) *m/z:* [M+H]$^+$ Calculated for $C_{19}H_{23}NO_7$ 378.1547 Found 378.1546.

(-) - dimethyl (E)-(3-(4-acetoxyphenyl)acryloyl)-L-glutamate (30c): Starting from (E)-3-(4-acetoxyphenyl)acrylic acid (30). Yellow solid. Yield: 81%. m.p.: 100-102°C (1PS:1EA). $[\alpha]^{25}_{589}$ = -18° (10^{-2} g/mL in MeOH). IR (KBr, cm^{-1}): 3309.4, 1751.6, 1655.9, 1624.7. ^{1}H – NMR (500 MHz, $CDCl_3$): δ 7.59 (d, *J* = 15.6 Hz, 1H), 7.49 (d, *J* = 8.6 Hz, 2H), 7.09 (d, *J* = 8.6 Hz, 2H), 6.53 (d, *J* = 7.7 Hz), 6.37 (d, *J* = 15.6 Hz, 1H), 4.76 (td, J_1 = 7.9 Hz, J_2 = 5.1 Hz, 1H), 3.76 (s, 3H), 3.66 (s, 3H), 2.51 – 2.37 (m, 2H), 2.30 (s, 3H), 2.29 – 2.23 (m, 1H), 2.11-2.03 (m, 1H). ^{13}C – NMR (125 MHz, $CDCl_3$): δ 174.0, 172.5, 169.4, 165.7, 151.9, 141.0, 132.5, 129.1, 122.2, 120.1, 52.8, 52.0, 30.2, 27.5, 21.3. HRMS (ESI) *m/z:* [M+H]$^+$ Calculated for $C_{18}H_{22}NO_7$ 364.1391 Found 364.1403.

30c

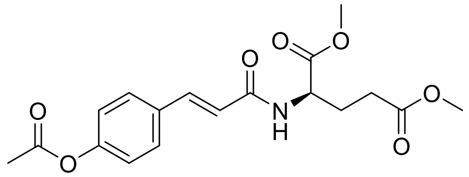

References – ***The references used in the Supplementary section are numbered according to their position in the main manuscript. The reader is advised to read the main manuscript's reference list.***
